# Microwave-assisted Cu(I)-catalyzed, three-component synthesis of 2-(4-((1-phenyl-1*H*-1,2,3-triazol-4-yl)methoxy)phenyl)-1*H*-benzo[*d*]imidazoles

**DOI:** 10.3762/bjoc.10.145

**Published:** 2014-06-24

**Authors:** Yogesh Kumar, Vijay Bahadur, Anil Kumar Singh, Virinder Singh Parmar, Erik V Van der Eycken, Brajendra Kumar Singh

**Affiliations:** 1 Bioorganic Laboratory, Department of Chemistry, University of Delhi, Delhi 110 007, India; 2 Laboratory for Organic & Microwave-Assisted Chemistry (LOMAC), Department of Chemistry, University of Leuven (KU Leuven), Celestijnenlaan 200F, B-3001 Leuven, Belgium

**Keywords:** benzimidazole, Cu(I) catalysis, microwave-assisted synthesis, multicomponent, three component synthesis

## Abstract

A microwave-assisted synthesis of 2-(4-((1-phenyl-1*H*-1,2,3-triazol-4-yl)methoxy)phenyl)-1*H*-benzo[*d*]imidazoles from a phenylazide, propargyloxybenzaldehyde and a 1,2-diaminobenzene is proposed.

## Introduction

Due to their structural range and biological importance nitrogen-containing heterocycles have been striking targets for many years. They are found in a variety of natural products and are characterized by an appreciable chemical and biological importance. The synthesis of nitrogen-containing heterocyclic compounds and their derivatives plays an important role in organic chemistry as they frequently exhibit therapeutic and pharmacological properties. They have emerged as an integral backbone of several existing drugs. Various medicinal agents are composed of several heterocyclic rings in which the benzimidazole and the 1,2,3-triazole constitute an important position. Benzimidazole derivatives have been shown to posses anticancer [1–2], antihypertensive [3], antibacterial [4] and enzyme inhibition activity [5–6]. They have also been used to synthesize dyes [7], chemosensitizers [8] and fluorophores [9]. Triazole derivatives have shown antifungal [10], anticancer [11] antituberculosis [12] and antimicrobial [13] activities. Recently, hybrid molecules, connecting two or more distinct drug entities in one molecule, have drawn the attention of medicinal chemists [14–18]. This logical approach is a promising path for those drug molecules which can effectively and selectively target multifunctional diseases. It has also been found that hybrid molecules are sometimes much more effective than the sum of their individual components.

The therapeutic application of 2-(3-ﬂuoro-phenyl)-1-[1-(substituted-phenyl)-1*H*-[1,2,3]-triazol-4-yl-methyl)-1*H*-benzo*[d]*imidazoles has been demonstrated by treating tuberculosis[19]. However, there has been little progress in the development of such hybrid molecules to date. An extensive literature survey revealed the existence of a multistep synthesis with low yields and long reaction times. This encouraged us to develop a new methodology for this synthesis.

## Results and Discussion

**Scheme 1:**
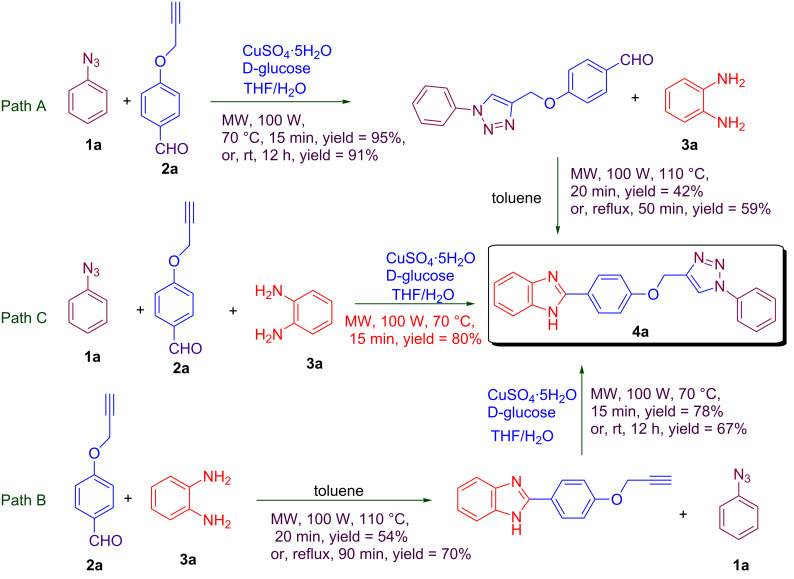
Synthesis of 2-(4-((1-phenyl-1*H*-1,2,3-triazol-4-yl)methoxy)phenyl)-1*H*-benzo[*d*]imidazoles.

Three different approaches for the construction of the proposed 2-(4-((1-phenyl-1*H*-1,2,3-triazol-4-yl)methoxy)phenyl)-1*H*-benzo[*d*]imidazole are illustrated in Scheme 1. In a two-step process the triazole and imidazole ring are synthesized consecutively (Scheme 1, path A and B). However, we reasoned that the desired adduct could also be formed in a one-pot fashion (Scheme 1, path C) as a multicomponent reaction (MCR). The utility and importance of MCRs have been recognized by chemists [20–23]. Several MCRs are now well-established reactions, such as Ugi [24], Passerini [25], Van Leusen [26], Strecker [27], Hantzsch [28], and Biginelli [29–31].

However, when path A and path B were explored, the desired product was afforded in different yields (Scheme 1). The treatment of acetylene **2a** with phenylazide (**1a**) in the presence of copper sulfate and D-glucose as a reductant [32–33] in THF/H_2_O (2:1) as a solvent under stirring at rt as well as under microwave irradiation resulted in the obtainment of the desired product in excellent yields of 91% and 95% in 12 h and 15 min, respectively. However, when the manufactured 4-((1-phenyl-1*H*-1,2,3-triazol-4-yl)methoxy)benzaldehyde was treated with 1,2-diaminobenzene, the desired product was obtained in an inferior yield of 59% and 42% under conventional heating and microwave irradiation in 50 and 20 min, respectively (path A). On the other hand, when 4-(prop-2-yn-1-yloxy)benzaldehyde (**2a**) was first treated with 1,2-diaminobenzene in the presence of copper sulfate and D-glucose in a THF/H_2_O (2:1) mixture under conventional heating as well as microwave irradiation, the desired product was obtained in a better yield (70%) under conventional heating compared to microwave irradiation (54% yield). The compound was subsequently coupled with phenylazide (**1a**), which afforded the desired product in 67% and 78% yield upon stirring at rt and microwave irradiation, respectively (path B). However, in the MCR approach (Scheme 1, path C) the desired product was obtained in a good yield. The reaction proceeded smoothly in the presence of CuSO_4_^.^5H_2_O and D-glucose under microwave irradiation for 15 min and gave the desired compound in 80% yield. Surprisingly, under conventional heating with this MCR approach no product formation was observed, even after an extended period of time (24 h) with heating under reflux .

In order to optimize the reaction conditions for this protocol, we screened several organic solvents. We explored the reaction between phenylazide (**1a)**, 4-(prop-2-yn-1-yloxy)benzaldehyde (**2a**) and 1,2-diaminobenzene (**3a**). It was found that when the reactions were carried out in polar solvents, such as acetonitrile, *N*,*N*-dimethylformamide (DMF), dimethylsulfoxide (DMSO) or 1,4-dioxane, no product formation was observed (Table 1, entries 1–4). However, upon microwave irridiation the reaction went to completion in a non-polar solvent, such as tetrahydrofuran (THF) or toulene, and the desired product was isolated in 20% and 25% yields (Table 1, entry 5 and entry 6) in THF and toluene, respectively. Moreover, when the reaction was carried out in an aqueous solvent system, decent improvements of the yields were observed (Table 1, entries 7–11). The best result was obtained with THF/H_2_O 2:1 (Table 1, entry 8). It is believed that the higher solubility of CuSO_4_ in this aqueous solvent system is responsible for the enhanced product formation. On the contrary, the formation of aggregates of the copper acetylide intermediate in polar solvents results in a failure of the reaction [34].

**Table 1:** Optimization of the solvent system.^a^

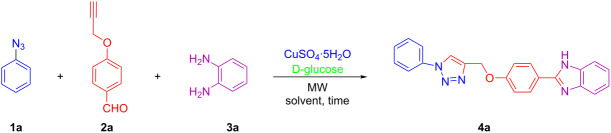			

Entry	Solvent	Time (min)/temperature (^o^C)	Yield (%)^b^

1	Acetonitrile	30/80	0
2	DMF	15/100	0
3	DMSO	15/100	0
4	1,4-Dioxane	15/110	0
5	THF	20/70	20
6	Toluene	20/100	25
7	Toluene/H_2_O 2:1	20/100	56
**8**	**THF/H_2_O 2:1**	**15/70**	**80**
9	DMF/H_2_O 2:1	15/100	30
10	DMSO/H_2_O 2:1	15/100	25
11	1,4-Dioxane/H_2_O 2:1	15/100	40

^a^Phenylazide (**1a**, 1.0 mmol), 4-(prop-2-yn-1-yloxy)benzaldehyde (**2a**, 1.2 mmol), 1,2-diaminobenzene (**3a**, 2 mmol), CuSO_4_·5H_2_O (0.2 equiv), D-glucose (0.4 equiv) in different solvents were irradiated for the indicated time and temperature at 100 W maximum power; ^b^isolated yields.

Various 1,2-diaminobenzenes **3a**,**b** and phenylazides **1a–j** were explored in order to estabilish the applicability of this protocol and the results are summarized in Table 2. Different azides **1a–j** with electron-donating groups (Table 2, entries 2–8, 12–17, 20–23), electron-withdrawing groups (Table 2, entries 9, 10, 18 and 19), two different 4-(prop-2-yn-1-yloxy)benzaldehydes **2a**,**b**, and two different 1,2-diaminobenzenes **3a**,**b** were used. In general, good to excellent yields were obtained for the desired cyclized products.

**Table 2:** Scope and limitations of the protocol employing different 4-(prop-2-yn-1-yloxy)benzaldehydes (**2**), phenylazides (**1**) and 1,2-diaminobenzenes (**3**)^a^.

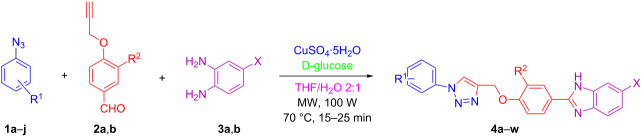					

Entry	R^1^	R^2^	X	Product	Yield (%)^b^

1	H	H	H	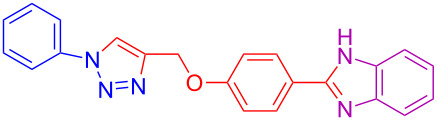 **4a**	80
2	4-OCH_3_	H	H	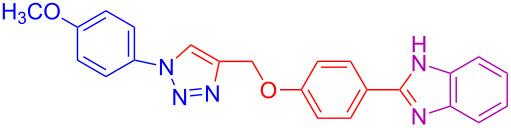 **4b**	92
3	3-OCH_3_	H	H	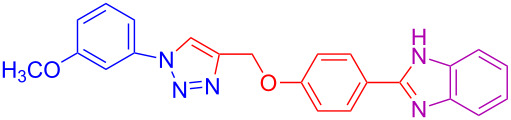 **4c**	83
4	2-OCH_3_	H	H	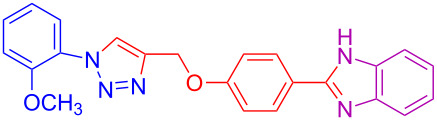 **4d**	75
5	4-CH_3_	H	H	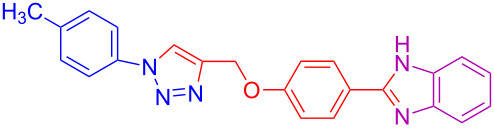 **4e**	79
6	3-CH_3_	H	H	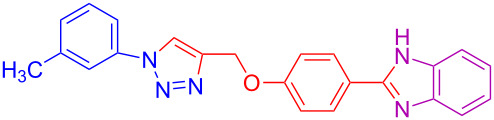 **4f**	68^c^
7	2-CH_3_	H	H	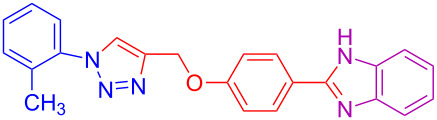 **4g**	60
8	4-Br	H	H	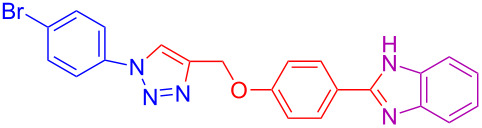 **4h**	75
9	3-Cl	H	H	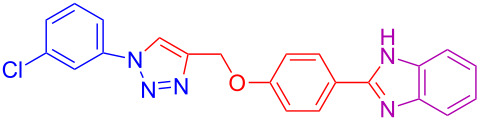 **4i**	73
10	2-F	H	H	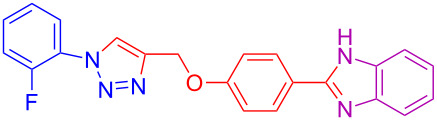 **4j**	60^c^
11	H	H	Cl	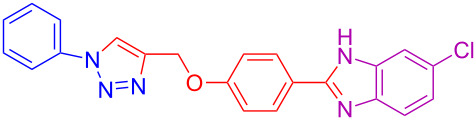 **4k**	90
12	4-OCH_3_	H	Cl	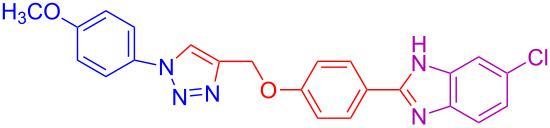 **4l**	91
13	3-OCH_3_	H	Cl	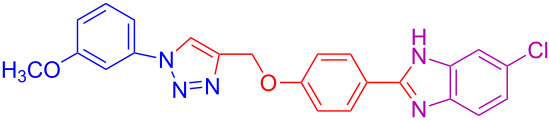 **4m**	73
14	2-OCH_3_	H	Cl	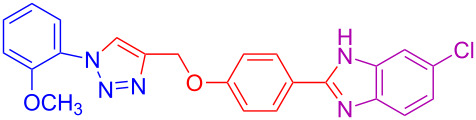 **4n**	76^c^
15	4-CH_3_	H	Cl	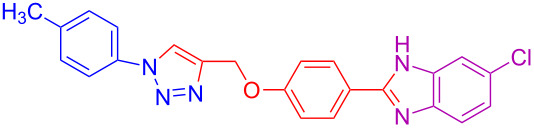 **4o**	82
16	3- CH_3_	H	Cl	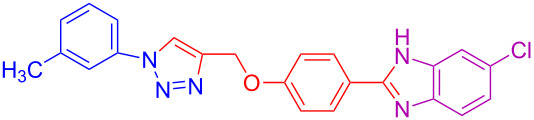 **4p**	69
17	4-Br	H	Cl	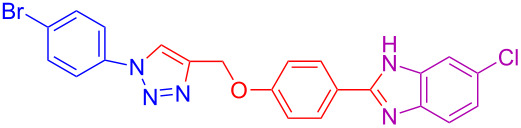 **4q**	78
18	3-Cl	H	Cl	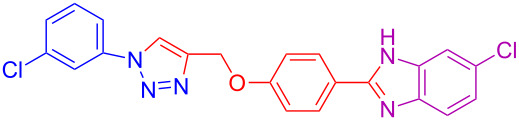 **4r**	77
19	2-F	H	Cl	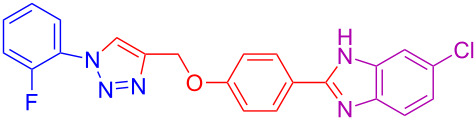 **4s**	67^c^
20	4-CH_3_	OCH_3_	H	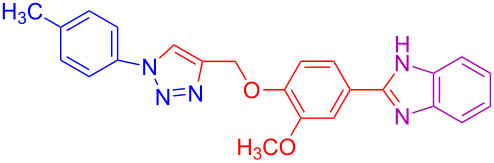 **4t**	69
21	4-OCH_3_	OCH_3_	H	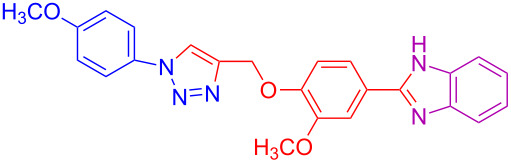 **4u**	83^c^
22	4-CH_3_	OCH_3_	Cl	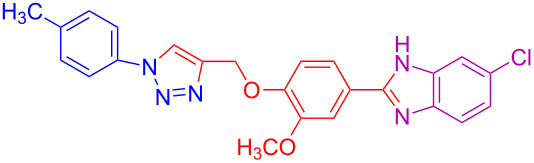 **4v**	68
23	4-OCH_3_	OCH_3_	Cl	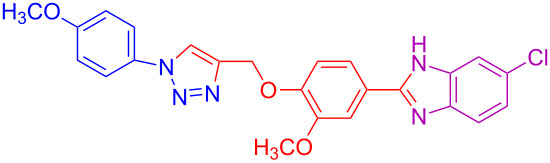 **4w**	85

^a^Phenylazide **1** (1.0 mmol), propargyloxybenzaldehyde **2** (1.2 mmol), 1,2-diaminobenzene **3** (2 mmol), CuSO_4_·5H_2_O (0.2 equiv), D-glucose (0.4 equiv) were irradiated at 70 °C and 100 W maximum power; ^b^isolated yields after work-up, no further purification was required; ^c^Isolated yields after column chromatography.

### Plausible mechanism

**Scheme 2:**
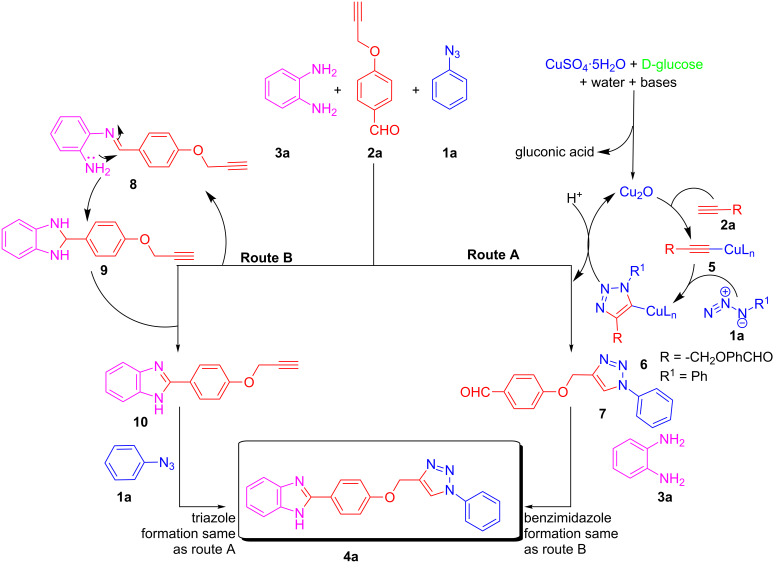
Plausible mechanism for the synthesis of 2-(4-((1-phenyl-1*H*-1,2,3-triazol-4-yl)methoxy)phenyl)-1*H*-benzo[*d*]imidazole.

The desired product could be obtained by the two mechanistic pathways A and B as described in Scheme 2. The CuAAC could take place prior to or after benzimidazole formation and we do not have a clear mechanistic proof. However, we believe that if the reaction proceed via route A in situ generation of Cu(I) [32–33] from Cu(II) takes place first upon reduction with D-glucose. Then, this Cu(I) reacts with 4-(prop-2-yn-1-yloxy)benzaldehyde **2a** to form the copper acetylide [35–36] **5**, which reacts with azidobenzene **1a** affording intermediate **6** by a [3 + 2] cycloaddition reaction. The intermediate **6** yields 4-((1-phenyl-1*H*-1,2,3-triazol-4-yl)methoxy)benzaldehyde intermediate **7** after protonolysis of the C–Cu bond. This intermediate reacts with 1,2-diaminobenzene (**3a**) under the formation of the corresponding Schiff’ base, which further cyclizes to dihyrobenzimidazole. Finally, D-glucose [37] oxidizes the dihyrobenzimidazole to the benzimidazole. Moreover, if the reaction proceeds via route B the benzimidazole formation from 4-(prop-2-yn-1-yloxy)benzaldehyde **2a** and 1,2-diaminobenzene **3a** takes place first, followed by the formation of triazole by CuAAC reaction to give the desired product **4a**.

## Conclusion

We developed a novel microwave-assisted, Cu(I)-catalyzed, three-component reaction for the synthesis of 2-(4-((1-phenyl-1*H*-1,2,3-triazol-4-yl)methoxy)phenyl)-1*H*-benzo[*d*]imidazoles in good to excellent yields. This protocol is applicable to various phenylazides, propargyloxybenzaldehydes and 1,2-diaminobenzenes.

## Supporting Information

File 1 Experimental procedures and analytical data.
