# Supplementary material for: Microwave-assisted Cu(I)-catalyzed, three-component synthesis of 2-(4-((1-phenyl-1H-1,2,3-triazol-4-yl)methoxy)phenyl)-1H-benzo[d]imidazoles
Source: Beilstein J Org Chem. 2014 Jun 24;10:1413–20. doi: 10.3762/bjoc.10.145 (PMC4077363; doi:10.3762/bjoc.10.145)
Supplement: File 1 — Experimental procedures and analytical data. [file Beilstein_J_Org_Chem-10-1413-s001.pdf]

Supporting Information

Microwave-Assisted Cu(I)-Catalyzed, Three-Component Synthesis of 2-(4-((1-phenyl-1*H*-1,2,3-triazol-4-yl)methoxy)phenyl)-1*H*-benzo[*d*]imidazoles

Yogesh Kumar,[1] Vijay Bahadur,[1] Anil K. Singh,[1] Virinder S. Parmar,[1] Erik V. Van der Eycken[2] and Brajendra K. Singh*,[1]

[1]*Bioorganic Laboratory, Department of Chemistry, University of Delhi, Delhi 110 007, India*

[2]*Laboratory for Organic & Microwave-Assisted Chemistry (LOMAC), Department of Chemistry, University of Leuven (KU Leuven), Celestijnenlaan 200F, B-3001 Leuven, Belgium*

* *Corresponding authors: Email id*

singhbk@chemistry.du.ac.in

Experimental procedures and analytical data

Contents:

1. General considerations 2

2. Experimental procedures 2

3. References 3

3. Analytical data 4

4. Copies of NMR spectra for new compound 13

1. General considerations

Analytical TLCs were performed on Merck silica gel 60_{F254} plates. IR spectra were recorded on a Perkin-Elmer 2000 FT-IR spectrometer at Department of Chemistry, University of Delhi. The ^{1}H and ^{13}C NMR spectra (in DMSO-d_6) were recorded on a JEOL ECX-400P NMR at 400 MHz and 100 MHz respectively at USIC, University of Delhi, TMS was used as internal standard. The NMR specta were processed by JEOL Delta™ NMR data processing softwares. The chemical shift values are on a δ scale and coupling constant (*J*) are in Hz. Abbreviations used are: s (singlet), d (doublet), t (triplet), dd (double doublet) and m (multiplet). The high-resolution mass spectra analysis was obtained JEOL JMS-SX-102A spectrometer at Institut fur Chemie und Biochemie, Free University Berlin, Takustr. 3, 14195, Berlin, Germany. Melting points were recorded on a Buchi M-560 melting point apparatus. All the chemical reagents were purchased from commercial sources and used as received unless otherwise indicated.

2. Experimental procedures:

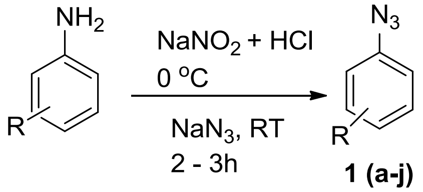

R = H, 4-OCH_3, 3-OCH_3, 2-OCH_3, 4-CH_3, 3-CH_3, 2-CH_3, 4-Br, 3-Cl, 2-F

[2.1] ***General procedure for the synthesis of Azidobenzenes*[1] 1 (a-j):** A mixture of appropriate aniline (1 mmol) in 17 % HCl was stirred at 0 °C, aqueous sodium nitrite (1.5 mmol) was added dropwise and stirring continued at 0 °C. After 15 min, aqueous sodium azide (1.5 mmol) was added dropwise at 0 °C and contents stirred for 3 - 4 hours. The progress of the reaction was monitored by TLC [ethyl acetate / petroleum ether (1 : 5)]. After completion of reaction, it was extracted with ethyl acetate (3 x 50 mL). The combined ethyl acetate layer was dried over Na_2SO_4, concentrated under reduced pressure and used directly in the next step without any further purification.

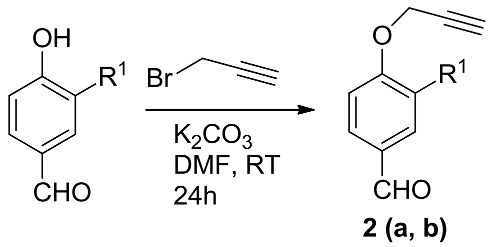

R = H, 2-OCH_3 if R_1 = 4-CHO

R = 2-CHO if R_1 = H

[2.2] ***General procedure for the synthesis of 4-(prop-2-yn-1-yloxy)benzaldehydes*[2] 2 (a, b):** A mixture of appropriate hydroxy benzaldehyde (1 mmol) and propargyl bromide (1.2 mmol) in DMF as a solvent was stirred with K_2CO_3 at room temperature for 20 – 24 hours. The progress of the reaction was monitored by TLC [ethyl acetate / petroleum ether (1 : 4)]. After completion of reaction, the mixture was poured into the ice/cold water & the solid product is separated, filtered out and dried. It was used directly in the next step without any further purification.

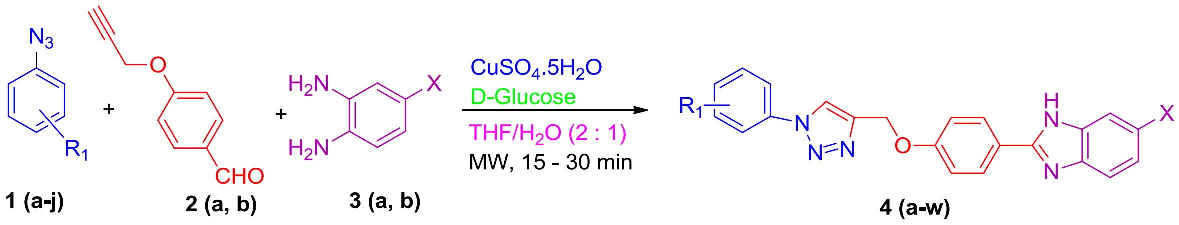

[2.3] ***A typical procedure for the synthesis of 2-(4-((1-phenyl-1H-1,2,3-triazol-4-yl)methoxy)phenyl)-1H-benzo[d]imidazoles* {4 (a-w)}:** A mixture of appropriate alkyne aldehyde (500 mg, 3.1 mmol), appropriate azide (410 mg, 3.4 mmol), appropriate 1,2-diamino arene (405 mg, 3.7 mmol) in presence of $CuSO_4$ (156 mg, 0.6 mmol), D-Glucose (225 mg, 1.2 mmol), in THF/H_2O (2 : 1) as a solvent was irradiated in microwave 70 ^oC, 100 watt/maximum power for 10 - 15 min. After the completion of reaction [monitored by TLC, methanol / chloroform (5 %)], and solvent was evaporated under reduced pressure. Residue was poured into water and the precipitate obtained was filtered. Precipitate was washed with chloroform (3 times) and pure product was obtained 4(a-w).

Refrences:

[1] Haridas, V.; Sahu, S.; Kumar, P. P. P. *Tetrahedron Lett.*, 2011, **52**, 6930-6934.

[2] (a) Pal, M.; Parasuraman, K.; Yeleswarapu, K. R. *Org. Lett.*, 2003, **5**, 349-352 (b) Bandaya, A. H.; Shameema, S. A.; Gupta, B. D.; Kumar, H. M. S. *Steroids*, 2010, **75**, 801-

804 (c) Beena.; Kumar, N.; Rohilla, R. K.; Roy, N.; Rawat, D. S. *Bio. Med. Chem. Lett.* **2009**, *19*, 1396-1398.

3. Analytical data:

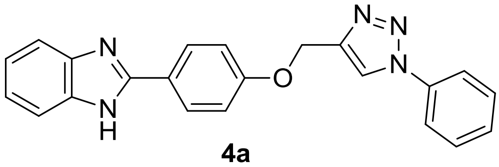

4a

[3.1] **2-(4-((1-phenyl-1*H*-1,2,3-triazol-4-yl)methoxy)phenyl)-1*H*-benzo[*d*]imidazole (4a) :** It was obtained as light brown solid having m. p. 198.5 – 200.5 ^{o}C in 80 % yield, **IR** (KBr) ν_{max} (cm^{-1}) = 3338, 1609 (C=C), 1263, 1044. **^{1}H NMR (400 MHz, DMSO-*d$_6$*)** δ 12.77 (s, 1H, -NH), 8.97 (s, 1H), 8.13 (d, *J* = 8.8 Hz, 2H), 7.91 (d, *J* = 8.8 Hz, 2H), 7.62-7.47 (m, 5H), 7.26 (d, *J* = 8.4 Hz, 2H), 7.16 (d, *J* = 1.4 Hz, 2H), 5.32 (s, 2H); **^{13}C NMR (100 MHz, DMSO-*d$_6$*)** δ 159.31, 151.24, 143.63, 136.55, 130.56, 129.90, 128.77, 128.04, 123.13, 123.01, 121.79, 120.18, 115.15, 61.16; **HRMS** calcd for $C_{22}H_{17}N_5OH$: 368.1511; found [M+H]$^+$: 368.1519.

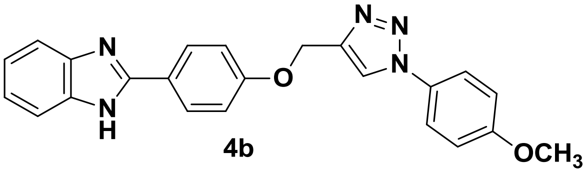

4b

[3.2] **2-(4-((1-(4-methoxyphenyl)-1*H*-1,2,3-triazol-4-yl)methoxy)phenyl)-1*H*-benzo[*d*]imidazole (4b) :** It was obtained as light yellow solid having m. p. 237.0 - 239.0 ^{o}C in 92 % yield, **IR** (KBr) ν_{max} (cm^{-1}) = 3221, 1612 (C=C), 1242, 1012. **^{1}H NMR (400 MHz, DMSO-*d$_6$*)** δ 12.74 (s, 1H, -NH), 8.87 (s, 1H), 8.13 (d, *J* = 8.8 Hz, 2H), 7.81 (d, *J* = 8.8 Hz, 2H), 7.26 (d, *J* = 7.32 Hz, 1H), 7.49 (d, *J* = 7.32 Hz, 1H), 7.25 (d, *J* = 8.8 Hz, 2H),7.16-7.11 (m,4H), 5.30 (s, 2H), 3.81 (s, 3H, -OCH_3); **^{13}C NMR (100 MHz, DMSO-*d$_6$*)** δ 162.30, 159.34, 151.30, 143.39, 141.56, 131.99, 129.98, 128.07, 126.54, 125.20, 123.14, 122.97, 121.85, 119.22, 115.16, 114.88, 61.21, 55.53; **HRMS** calcd for $C_{23}H_{19}N_5O_2H$: 398.1617; found [M+H]$^+$: 398.1621.

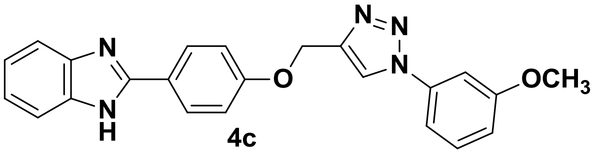

4c

[3.3] 2-(4-((1-(3-methoxyphenyl)-1*H*-1,2,3-triazol-4-yl)methoxy)phenyl)-1*H*-benzo[*d*]imidazole (4c) : It was obtained as light brown solid having m. p. 196.5-198.5 °C in 83 % yield, **IR** (KBr) ν_{max} (cm^{-1}) = 3257, 1609 (C=C), 1244, 1006. **^{1}H NMR (400 MHz, DMSO-*d*$_6$)** δ 9.91 (s, 1H), 8.14 (d, *J* = 8.7 Hz, 2H), 7.60-7.48 (m, 5H), 7.25 (d, *J* = 8.7 Hz, 2H), 7.16 (d, *J* = 4.90 Hz, 2H), 7.06-7.03 (m, 1H), 7.49 (d, *J* = 7.32 Hz, 1H), 5.32 (s, 2H), 3.84 (s, 3H, $-OCH_3$); **^{13}C NMR (100 MHz, DMSO-*d*$_6$)** δ 160.20, 159.31, 151.25, 143.59, 137.60, 130.87, 128.05, 123.14, 122.10, 121.50, 118.53, 115.16, 114.52, 112.12, 111.07, 105.77, 61.18, 55.62; **HRMS** calcd for $C_{23}H_{19}N_5O_2H$: 398.1617; found $[M+H]^+$: 398.1627.

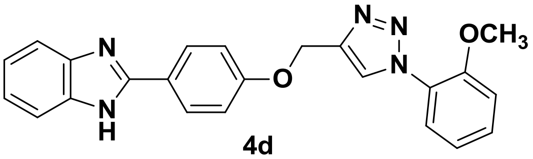

4d

[3.4] 2-(4-((1-(2-methoxyphenyl)-1*H*-1,2,3-triazol-4-yl)methoxy)phenyl)-1*H*-benzo[*d*]imidazole (4d) : It was obtained as brown solid having m. p. 234.0-236.0 °C in 75 % yield, **IR** (KBr) ν_{max} (cm^{-1}) = 3197, 1609 (C=C), 1255, 1048. **^{1}H NMR (400 MHz, DMSO-*d*$_6$)** δ 12.82 (s, 1H, -NH), 8.63 (s, 1H), 8.14 (d, *J* = 8.8 Hz, 2H), 7.65-7.48 (m, 4H), 7.33 (d, *J* = 7.32 Hz, 1H), 7.27 (d, *J* = 8.8 Hz, 2H), 7.18-7.12 (m, 3H), 5.31 (s, 2H), 3.85 (s, 3H, $-OCH_3$); **^{13}C NMR (100 MHz, DMSO-*d*$_6$)** δ 159.38, 151.66, 143.87, 130.85, 128.01, 126.82, 125.84, 125.59, 123.06, 122.12, 121.47, 120.89, 118.53, 115.15, 113.01, 111.05, 61.04, 56.12; **HRMS** calcd for $C_{23}H_{19}N_5O_2H$: 398.1617; found $[M+H]^+$: 398.1598.

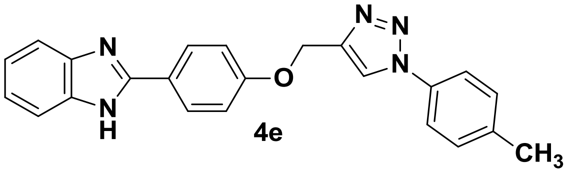

4e

[3.5] 2-(4-((1-(*p*-tolyl)-1*H*-1,2,3-triazol-4-yl)methoxy)phenyl)-1*H*-benzo[*d*]imidazole (4e) : It was obtained as yellow solid having m. p. 190.0-192.0 °C in 79 % yield, **IR** (KBr) ν_{max} (cm^{-1}) = 3309, 1609 (C=C), 1245, 1013. **^{1}H NMR (400 MHz, DMSO-*d*$_6$)** δ 8.94 (s, 1H), 8.17 (br. s, 2H), 7.79 (d, *J* = 8.08 Hz, 2H), 7.66 (br. s, 2H), 7.40 (d, *J* = 8.08 Hz, 2H), 7.29 (m, 4H), 5.33 (s, 2H), 2.36 (s, 3H, $-CH_3$); **^{13}C NMR (100 MHz, DMSO-*d*$_6$)** δ 160.08, 143.33, 134.24, 130.23, 128.59, 123.04, 120.82, 115.37, 114.60, 61.20, 20.53; **HRMS** calcd for $C_{23}H_{19}N_5OH$: 382.1667; found $[M+H]^+$: 382.1672.

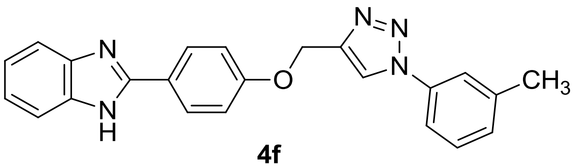

4f

[3.6] **2-(4-((1-(*m*-tolyl)-1*H*-1,2,3-triazol-4-yl)methoxy)phenyl)-1*H*-benzo[d]imidazole (4f) :** It was obtained as brown solid having m. p. 176.5.0-178.5 °C in 71 % yield, **IR** (KBr) ν_{max} (cm^{-1}) = 3104, 1609 (C=C), 1263, 1046. **^{1}H NMR (400 MHz, DMSO-*d*$_6$)** δ 8.96 (s, 1H), 8.18 (s, 2H), 7.76-7.48 (m, 5H), 7.31-7.21 (m, 5H), 5.34 (s, 2H), 2.42 (s, 3H, -CH_3); **^{13}C NMR (100 MHz, DMSO-*d*$_6$)** δ 160.02, 143.44, 139.67, 136.47, 129.69, 129.37, 128.58, 123.51, 122.92, 120.57, 117.23, 116.11, 115.37, 114.59, 61.22, 20.88; **HRMS** calcd for $C_{23}H_{19}N_5OH$: 382.1667; found [M+H]$^+$: 382.1672.

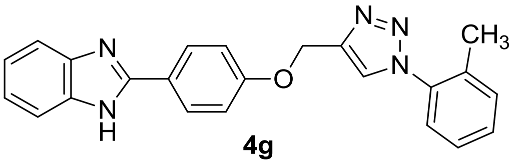

4g

[3.7] **2-(4-((1-(*o*-tolyl)-1*H*-1,2,3-triazol-4-yl)methoxy)phenyl)-1*H*-benzo[*d*]imidazole (4g) :** It was obtained as yellow solid having m. p. 260.0-262.0 °C in 60 % yield, **IR** (KBr) ν_{max} (cm^{-1}) = 3295, 1607 (C=C), 1247, 1050. **^{1}H NMR (400 MHz, DMSO-*d*$_6$)** δ 13.91 (br s, 1H, -NH), 8.65 (s, 1H), 8.17 (br s, 2H), 7.64-7.11 (m, 10H), 5.31 (s, 2H), 2.13 (s, 3H, -CH_3); **^{13}C NMR (100 MHz, DMSO-*d*$_6$)** δ 159.91, 153.60, 145.59, 142.42, 140.34, 136.18, 133.06, 131.37, 129.89, 128.69, 127.05, 126.49, 126.01, 122.72, 115.28, 113.51, 61.18, 17.29; **HRMS** calcd for $C_{23}H_{19}N_5OH$: 382.1667; found [M+H]$^+$: 382.1659.

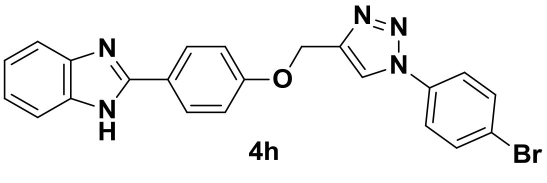

4h

[3.8] **2-(4-((1-(4-bromophenyl)-1*H*-1,2,3-triazol-4-yl)methoxy)phenyl)-1*H*-benzo[*d*]imidazole (4h) :** It was obtained as brown solid having m. p. 176.5-178.5 °C in 75 % yield, **IR** (KBr) ν_{max} (cm^{-1}) = 3316, 1608 (C=C), 1245, 1014. **^{1}H NMR (400 MHz, DMSO-*d*$_6$)** δ 9.04 (s, 1H), 8.16 (d, *J* = 8.8 Hz, 2H), 7.91-7.80 (m, 4H), 7.65 (m, 2H), 7.33-7.31 (m, 4H), 5.36 (s, 2H); **^{13}C NMR (100 MHz, DMSO-*d*$_6$)** δ 160.27, 143.67, 135.75,

132.87, 128.81, 123.35, 123.20, 122.15, 121.53, 115.51, 114.47, 61.23; **HRMS** calcd for $C_{22}H_{16}BrN_5OH$: 446.0616; found $[M+H]^+$: 346.0622.

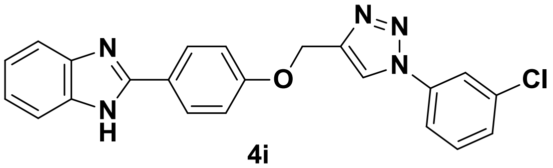

4i

[3.9] 2-(4-((1-(3-chlorophenyl)-1*H*-1,2,3-triazol-4-yl)methoxy)phenyl)-1*H*-benzo[*d*]imidazole (4i) : It was obtained as light brown solid having m. p. 172.0-174.0 °C in 73 % yield, **IR** (KBr) ν_{max} (cm^{-1}) = 3245, 1596 (C=C), 1254, 1047. **^{1}H NMR (400 MHz, DMSO-*d*$_6$)** δ 9.07 (s, 1H), 8.16 (d, *J* = 8.08 Hz, 2H), 8.06 (d, *J* = 8.08 Hz, 1H), 7.94 (d, *J* = 8.04 Hz, 2H), 7.65-7.56 (m, 4H), 7.29-7.24 (m, 4H), 5.35 (s, 2H); **^{13}C NMR (100 MHz, DMSO-*d*$_6$)** δ 159.73, 143.72, 137.55, 134.20, 131.67, 128.62, 128.33, 123.26, 122.54, 119.98, 118.78, 115.31, 114.67, 61.15; **HRMS** calcd for $C_{22}H_{16}ClN_5OH$: 402.1121; found $[M+H]^+$: 402.1119.

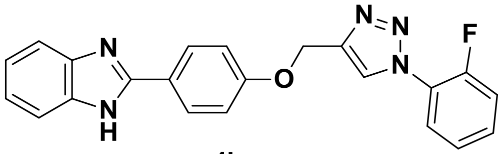

4j

[3.10] 2-(4-((1-(2-fluorophenyl)-1*H*-1,2,3-triazol-4-yl)methoxy)phenyl)-1*H*-benzo[*d*]imidazole (4j) : It was obtained as dark brown solid having m. p. 160.0-162.0 °C in 65 % yield, **IR** (KBr) ν_{max} (cm^{-1}) = 3403, 1611 (C=C), 1262, 1044. **^{1}H NMR (400 MHz, DMSO-*d*$_6$)** δ 8.79 (d, *J* = 1.3 Hz, 1H), 8.17 (br. s, 2H), 7.87 (m, 1H), 7.62-7.55 (m, 4H), 7.46 (t, 1H), 7.28-7.24 (m, 4H), 5.35 (s, 2H); **^{13}C NMR (100 MHz, DMSO-*d*$_6$)** δ 162.56, 159.46, 151.98, 143.47, 140.91, 137.34, 133.98, 131.44, 128.81, 128.12, 123.26, 122.26, 119.76, 118.39, 115.20, 113.75, 60.87 ; **HRMS** calcd for $C_{22}H_{16}FN_5OH$: 386.1617; found $[M+H]^+$: 386.1421.

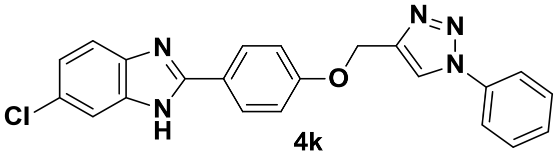

4k

[3.11] 6-chloro-2-(4-((1-phenyl-1*H*-1,2,3-triazol-4-yl)methoxy)phenyl)-1*H*-benzo[*d*]imidazole (4k) : It was obtained as light brown solid having m. p. 261.0-263.0 °C in

90 % yield, **IR** (KBr) ν_{max} (cm^{-1}) = 3231, 1608 (C=C), 1251, 1060. **^{1}H NMR (400 MHz, DMSO-*d*$_6$)** δ 8.99 (s, 1H), 8.13 (d, *J* = 8.79 Hz, 2H), 7.91 (d, *J* = 7.32 Hz, 2H), 7.61-7.55 (m, 4H), 7.51-7.47 (m, 1H), 7.27 (d, *J* = 8.79 Hz, 2H), 7.21-7.19 (dd, *J* = 8.79 Hz & 2.20 Hz, 1H), 5.33 (s, 2H); **^{13}C NMR (100 MHz, DMSO-*d*$_6$)** δ 159.78, 152.54, 143.58, 136.57, 129.98, 128.86, 128.40, 126.41, 123.11, 122.35, 122.09, 122.07, 120.22, 115.32, 114.65, 61.21; **HRMS** calcd for $C_{22}H_{16}ClN_5OH$: 402.1122; found $[M+H]^+$: 402.1129.

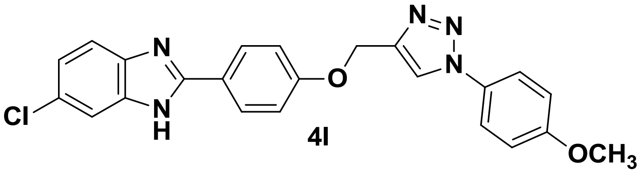

4l

[3.12] 6-chloro-2-(4-((1-(4-methoxyphenyl)-1*H*-1,2,3-triazol-4-yl)methoxy)phenyl)-1*H*-benzo[*d*]imidazole (4l) : It was obtained as green solid having m. p. 227 – 229 °C in 91 % yield, **IR** (KBr) ν_{max} (cm^{-1}) = 3228, 1615 (C=C), 1246, 1055. **^{1}H NMR (400 MHz, DMSO-*d*$_6$)** δ 8.86 (s, 1H), 8.12 (d, *J* = 8.70 Hz, 2H), 7.81-7.78 (m, 2H), 7.60 (d, *J* = 1.37 Hz, 1H), 7.456 (d, *J* = 8.24 Hz, 1H), 7.13-7.11 (m, 2H), 5.30 (s, 2H); 3.81 (s, 3H, -OCH_3); **^{13}C NMR (100 MHz, DMSO-*d*$_6$)** δ 159.73, 159.35, 143.31, 129.96, 128.32, 126.29, 123.05, 122.23, 121.87, 115.27, 114.48, 61.21, 55.58; **HRMS** calcd for $C_{23}H_{18}ClN_5O_2H$: 432.1227; found $[M+H]^+$: 432.1219.

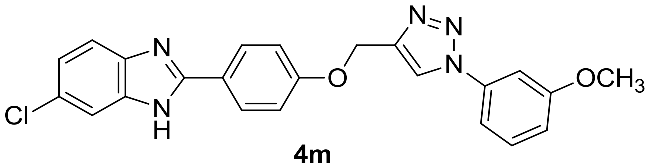

4m

[3.13] 6-chloro-2-(4-((1-(3-methoxyphenyl)-1*H*-1,2,3-triazol-4-yl)methoxy)phenyl)-1*H*-benzo[*d*]imidazole (4m): It was obtained as dark brown solid having m. p. 195.0-196.0 °C in 73 % yield, **IR** (KBr) ν_{max} (cm^{-1}) = 3310, 1609 (C=C), 1275, 1027. **^{1}H NMR (400 MHz, DMSO-*d*$_6$)** δ 9.02 (s, 1H), 8.13 (d, *J* = 8.79 Hz, 2H), 7.61-7.56 (m, 3H), 7.50-7.48 (m, 3H), 7.28 (d, *J* = 8.79 Hz, 2H), 7.22-7.20 (dd, J = 8.05 Hz & 1.46 Hz, 1H), 5.33 (s, 2H), 3.84 (s, 3H, -OCH_3); **^{13}C NMR (100 MHz, DMSO-*d*$_6$)** δ 160.20, 159.79, 143.50, 137.58, 130.89, 128.40, 126.43, 123.19, 122.38, 115.31, 114.46, 112.12, 105.78, 61.21, 55.63; **HRMS** calcd for $C_{23}H_{18}ClN_5O_2H$: 432.1227; found $[M+H]^+$: 432.1221.

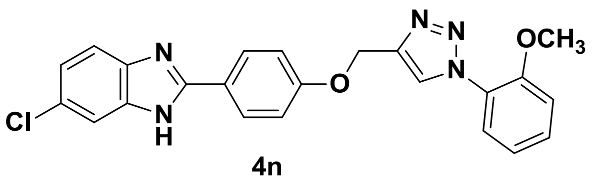

4n

[3.14] **6-chloro-2-(4-((1-(2-methoxyphenyl)-1*H*-1,2,3-triazol-4-yl)methoxy)phenyl)-1*H*-benzo[*d*]imidazole (4n) :** It was obtained as green solid, having m. p. 168.0-170.0 °C in 81 % yield, **IR** (KBr) ν_{max} (cm^{-1}) = 3245, 1608 (C=C), 1266, 1049. **^{1}H NMR (400 MHz, DMSO-*d$_6$*)** δ 8.63 (s, 1H), 8.15 (br. s, 2H), 7.66-7.52 (m, 3H), 7.32-7.22 (m, 5H), 7.18 (m, 1H), 5.32 (s, 2H); 3.84 (s, 3H, -OCH_3); **^{13}C NMR (100 MHz, DMSO-*d$_6$*)** δ 159.67, 151.54, 130.75, 128.22, 125.72, 120.78, 115.19, 112.89, 60.80, 56.01; **HRMS** calcd for $C_{23}H_{18}ClN_5O_2H$: 432.1227; found [M+H]$^+$: 432.1221.

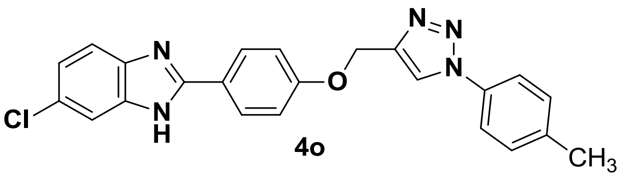

4o

[3.15] **6-chloro-2-(4-((1-(*p*-tolyl)-1*H*-1,2,3-triazol-4-yl)methoxy)phenyl)-1*H*-benzo[*d*]imidazole (4o) :** It was obtained as green solid having m. p. 196.5-198.5 °C in 82 % yield, **IR** (KBr) ν_{max} (cm^{-1}) = 3231, 1608 (C=C), 1250, 1059. **^{1}H NMR (400 MHz, DMSO-*d$_6$*)** δ 8.93 (s, 1H), 8.13 (d, *J* = 8.05 Hz, 2H), 7.78 (d, *J* = 8.05 Hz, 2H), 7.63 (s, 1H), 7.59 (d, *J* = 8.05 Hz, 1H), 7.39 (d, *J* = 8.05 Hz, 2H), 7.28 (d, *J* = 8.05 Hz, 2H), 7.23 (d, *J* = 8.05 Hz, 1H), 5.32 (s, 2H), 2.36 (s, 3H, -CH_3); **^{13}C NMR (100 MHz, DMSO-*d$_6$*)** δ 159.84, 143.63, 138.43, 134.27, 130.24, 128.39, 126.51, 122.94, 122.46, 120.02, 115.28, 61.18, 20.54; **HRMS** calcd for $C_{23}H_{18}ClN_5OH$: 416.1278; found [M+H]$^+$: 416.1269.

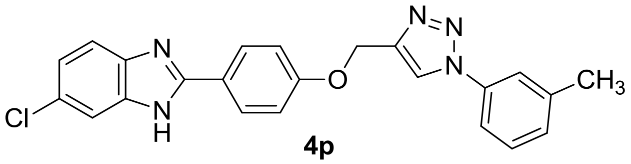

4p

[3.16] **6-chloro-2-(4-((1-(*m*-tolyl)-1*H*-1,2,3-triazol-4-yl)methoxy)phenyl)-1*H*-benzo[*d*]imidazole (4p) :** It was obtained as green solid having m. p. 179.5-181.5 °C in 69 % yield, **IR** (KBr) ν_{max} (cm^{-1}) = 3222, 1611 (C=C), 1250, 1058. **^{1}H NMR (400 MHz, DMSO-*d$_6$*)** δ 8.96 (s, 1H), 8.14 (d, *J* = 8.8 Hz, 2H), 7.75 (s, 1H), 7.70 (d, *J* = 8.04 Hz, 1H), 7.62-7.57 (m, 2H), 7.49 (t, *J* = 8.76 Hz, 1H), 7.31-7.19 (m, 4H), 5.33 (s, 2H), 2.38 (s, 3H, -CH_3); **^{13}C**

NMR (100 MHz, DMSO-d_6)** δ 159.74, 143.50, 139.73, 136.51, 129.75, 129.43, 128.40, 126.40, 123.03, 122.33, 120.62, 117.28, 115.86, 115.2961.21, 20.93; **HRMS** calcd for $C_{23}H_{18}ClN_5OH$: 416.1278; found $[M+H]^+$: 416.1270.

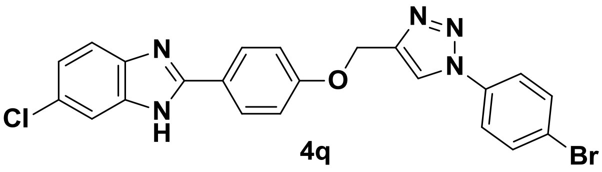

4q

[3.17] 2-(4-((1-(4-bromophenyl)-1*H*-1,2,3-triazol-4-yl)methoxy)phenyl)-6-chloro-1*H*-benzo[*d*]imidazole (4q) : It was obtained as brown solid having m. p. 246.5-248.5 °C in 78 % yield, **IR** (KBr) ν_{max} (cm^{-1}) = 3220, 1610 (C=C), 1249, 1057. **^{1}H NMR (400 MHz, DMSO-d_6)** δ 9.01 (s, 1H), 8.13 (d, *J* = 8.79 Hz, 2H), 7.90 (d, *J* = 8.79 Hz, 2H), 7.81 (d, *J* = 8.79 Hz, 2H), 7.65 (m, 1H), 7.58 (d, *J* = 8.05 Hz, 1H), 7.27 (d, *J* = 8.79 Hz, 2H), 7.23 (dd, *J* = 8.79 Hz & 1.46 Hz, 2H), 5.33 (s, 2H); **^{13}C NMR (100 MHz, DMSO-d_6)** δ 159.80, 143.75, 135.75, 132.84, 128.42, 126.50, 123.13, 122.44, 122.12, 121.94, 121.50, 115.31, 61.16; **HRMS** calcd for $C_{22}H_{15}BrClN_5OH$: 480.0227; found $[M+H]^+$: 480.0213.

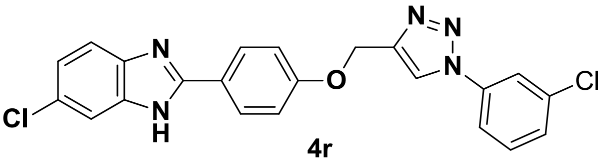

4r

[3.18] 6-chloro-2-(4-((1-(3-chlorophenyl)-1H-1,2,3-triazol-4-yl)methoxy)phenyl)-1H-benzo[d]imidazole (4r) : It was obtained as dark brown solid having m. p. 209.0-211.0 °C in 77 % yield, **IR** (KBr) ν_{max} (cm^{-1}) = 3234, 1596 (C=C), 1252, 1061. **^{1}H NMR (400 MHz, DMSO-d_6)** δ 9.06 (s, 1H), 8.13 (d, *J* = 8.05 Hz, 2H), 8.06 (m, 1H), 7.94 (d, *J* = 8.79 Hz, 1H), 7.64-7.55 (m, 4H), 7.27 (d, *J* = 8.05 Hz, 2H), 7.21 (d, *J* = 8.79 Hz, 1H), 5.33 (s, 2H); **^{13}C NMR (100 MHz, DMSO-d_6)** δ 159.73, 143.72, 137.53, 134.23, 131.67, 128.62, 128.34, 126.35, 123.25, 122.29, 119.98, 118.78, 115.29, 61.15; **HRMS** calcd for $C_{22}H_{15}Cl_2N_5OH$: 436.0732; found $[M+H]^+$: 436.0729.

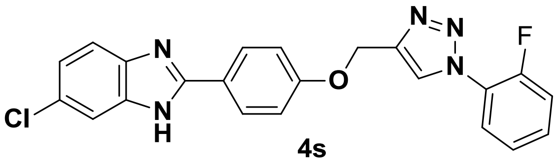

4s

[3.19] 6-chloro-2-(4-((1-(2-fluorophenyl)-1H-1,2,3-triazol-4-yl)methoxy)phenyl)-1H-benzo[d]imidazole (4s) : It was obtained as green solid having m. p. 179.0-181.0 ^{o}C in 69 % yield, **IR** (KBr) ν_{max} (cm^{-1}) = 3219, 1607 (C=C), 1259, 1057. **^{1}H NMR (400 MHz, DMSO-*d$_6$*)** δ 8.79 (d, *J* = 2.2 Hz, 1H), 8.14 (d, *J* = 8.79 Hz, 2H), 7.87-7.83 (m, 1H), 7.64-7.56 (m, 4H), 7.46-7.42 (m, 1H), 7.30 (d, *J* = 8.79 Hz, 2H), 7.25-7.22 (dd, *J* = 8.79 Hz & 1.46 Hz, 1H), 5.35 (s, 2H); **^{13}C NMR (100 MHz, DMSO-*d$_6$***) δ 159.90, 155.09, 152.60, 142.97, 131.39, 128.44, 126.60, 126.44, 126.04, 125.58, 122.55, 117.24, 117.05, 1115.30, 60.96; **HRMS** calcd for $C_{22}H_{15}ClFN_5OH$: 420.1027; found [M+H]$^{+}$: 420.1021.

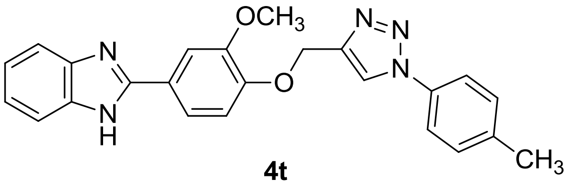

4t

[3.20] 2-(3-methoxy-4-((1-(*p*-tolyl)-1*H*-1,2,3-triazol-4-yl)methoxy)phenyl)-1*H*-benzo[*d*]imidazole (4t) : It was obtained as dark brown solid having m. p. 193.5-195.5 ^{o}C in 69 % yield, **IR** (KBr) ν_{max} (cm^{-1}) = 3386, 1605 (C=C), 1273, 1045. **^{1}H NMR (400 MHz, DMSO-*d$_6$*)** δ 8.93 (s, 1H), 7.79 (d, *J* = 8.05 Hz, 4H), 7.64 (s, 2H), 7.40 (d, *J* = 8.79 Hz, 3H), 7.25 (s, 2H), 5.30 (s, 2H), 3.86 (s, 3H, -OCH$_3$), 2.37 (s, 3H, -CH$_3$); **^{13}C NMR (100 MHz, DMSO-*d$_6$***) δ 149.20, 143.36, 138.49, 134.32, 130.29, 123.16, 122.65, 120.08, 119.68, 113.52, 110.17, 61.58, 55.63, 20.60; **HRMS** calcd for $C_{24}H_{21}N_5O_2H$: 412.1773; found [M+H]$^{+}$: 412.1767.

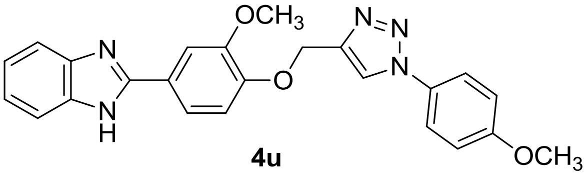

4u

[3.21] 2-(3-methoxy-4-((1-(4-methoxyphenyl)-1*H*-1,2,3-triazol-4-yl)methoxy)phenyl)-1*H*-benzo[*d*]imidazole (4u) : It was obtained as dark brown solid having m. p. 191.0-193.0 ^{o}C in 89 % yield, **IR** (KBr) ν_{max} (cm^{-1}) = 3385, 1616 (C=C), 1262, 1050. **^{1}H NMR (400 MHz, DMSO-*d$_6$*)** δ 8.81 (s, 1H), 7.79-7.76 (m, 3H), 7.52 (s, 2H), 7.32 (d, *J* = 8.54 Hz, 2H), 7.14-7.09 (m, 4H), 5.25 (s, 2H), 3.84 (s, 3H, -OCH$_3$), 3.79 (s, 3H, -OCH$_3$), 2.37; **^{13}C NMR (100 MHz, DMSO-*d$_6$***) δ 159.35, 149.20, 129.94, 123.21, 122.67, 121.87, 114.91, 114.65, 113.53,61.58, 55.57; **HRMS** calcd for $C_{24}H_{21}N_5O_3H$: 428.1722; found [M+H]$^{+}$: 428.1728.

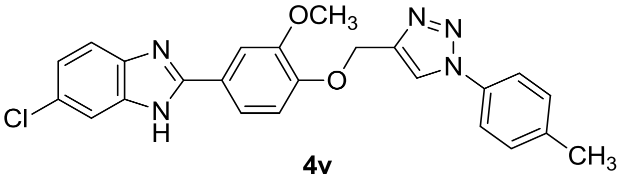

4v

[3.22] **6-chloro-2-(3-methoxy-4-((1-(*p*-tolyl)-1*H*-1,2,3-triazol-4-yl)methoxy)phenyl)-1*H*-benzo[*d*]imidazole (4v) :** It was obtained as green solid having m. p. 173.5-175.5 °C in 68 % yield, **IR** (KBr) ν_{max} (cm^{-1}) = 3148, 1608 (C=C), 1272, 1059. **^{1}H NMR (400 MHz, DMSO-*d*$_6$)** δ 8.92 (s, 1H), 7.79 (d, *J* = 8.05 Hz, 4H), 7.65 (s, 1H), 7.61 (d, *J* = 7.32 Hz, 1H), 7.40 (d, *J* = 8.05 Hz, 3H), 7.24 (d, *J* = 8.05 Hz, 1H), 5.29 (s, 2H), 3.87 (s, 3H, -OCH_3), 2.36 (s, 3H, -CH_3); **^{13}C NMR (100 MHz, DMSO-*d*$_6$)** δ 149.09, 138.39, 134.20, 130.18, 126.43, 123.08, 119.98, 113.40, 109.79, 61.45, 55.53, 20.48; **HRMS** calcd for $C_{24}H_{20}ClN_5O_2H$: 446.1383; found $[M+H]^+$: 446.1370.

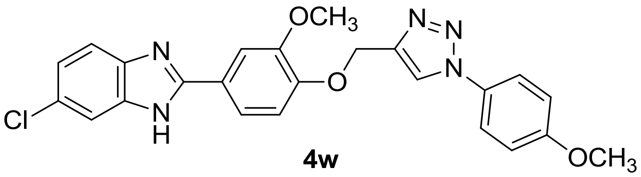

4w

[3.23] **6-chloro-2-(3-methoxy-4-((1-(4-methoxyphenyl)-1*H*-1,2,3-triazol-4-yl)methoxy)phenyl)-1*H*-benzo[*d*]imidazole (4w) :** It was obtained as dark brown solid having m. p. 187.6-189.6 °C in 85 % yield, **IR** (KBr) ν_{max} (cm^{-1}) = 3199, 1611 (C=C), 1259, 1059. **^{1}H NMR (400 MHz, DMSO-*d*$_6$)** δ 8.86 (s, 1H), 7.82-7.73 (m, 4H), 7.26 (s, 1H), 7.59 (d, *J* = 8.05 Hz, 1H), 7.39 (d, *J* = 8.05 Hz, 1H), 7.21 (d, *J* = 8.05 Hz, 1H), 7.14 (d, *J* = 8.76 Hz, 2H), 5.28 (s, 2H), 3.86 (s, 3H, -OCH_3), 3.82 (s, 3H, -OCH_3); **^{13}C NMR (100 MHz, DMSO-*d*$_6$)** δ 159.33, 149.30, 149.15, 129.94, 126.33, 123.20, 122.26, 121.85, 119.49, 114.88, 113.46,110.02, 61.57, 55.55; **HRMS** calcd for $C_{24}H_{20}ClN_5O_3H$: 462.1332; found $[M+H]^+$: 462.1356.

4. Copies of NMR spectra for new compound:

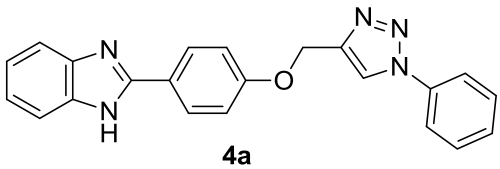

4a

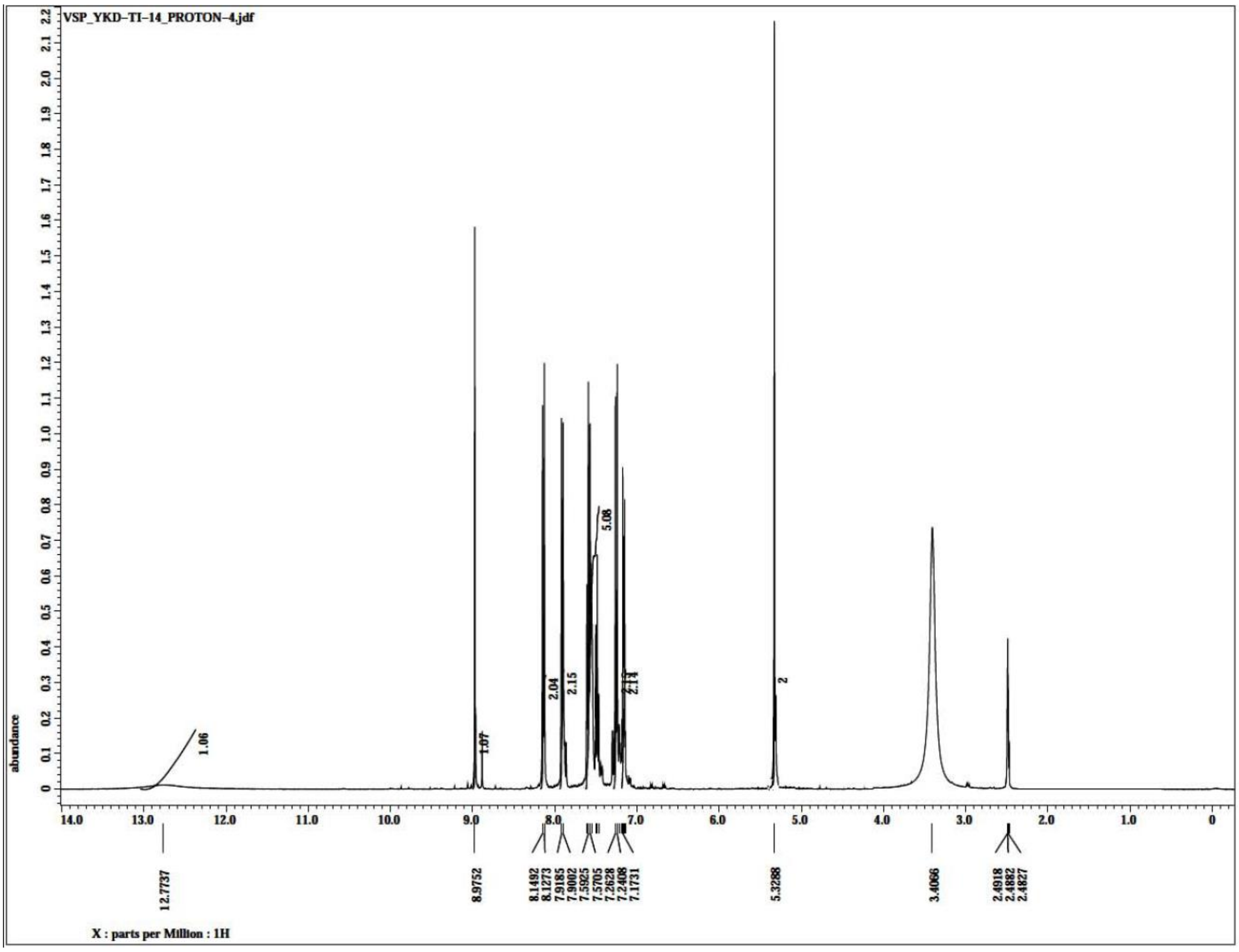

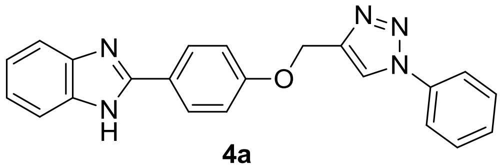

4a

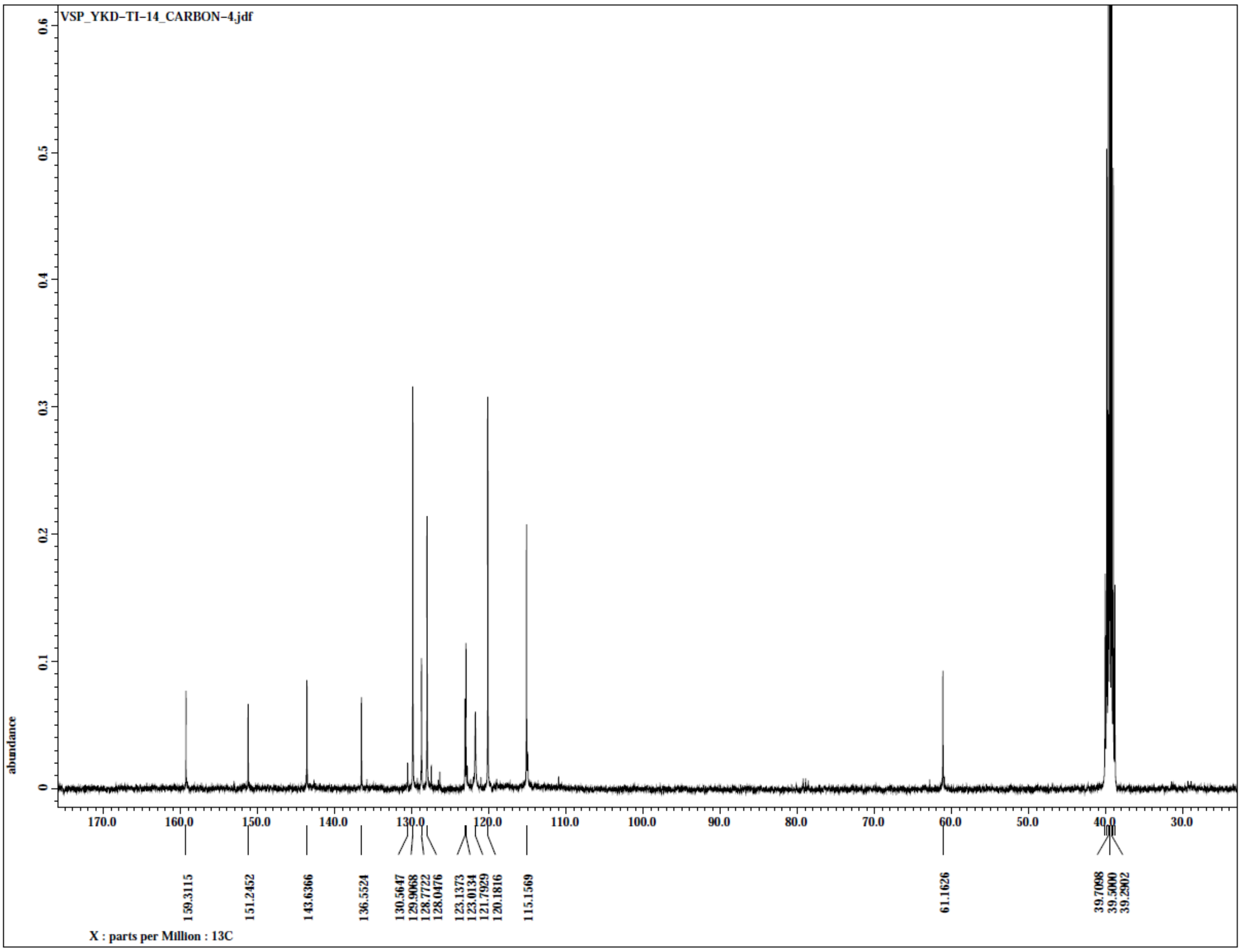

VSP_YKD-TI-14_CARBON-4.jdf
abundance
X : parts per Million : 13C
159.3115
151.2452
143.6366
136.5524
130.5647
129.9068
128.7722
128.0476
123.1373
123.0134
121.7929
120.1816
115.1569
61.1626
39.7098
39.5000
39.2902

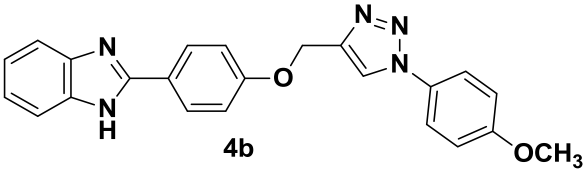
N
N
H
O
N
N
N
OCH3

4b

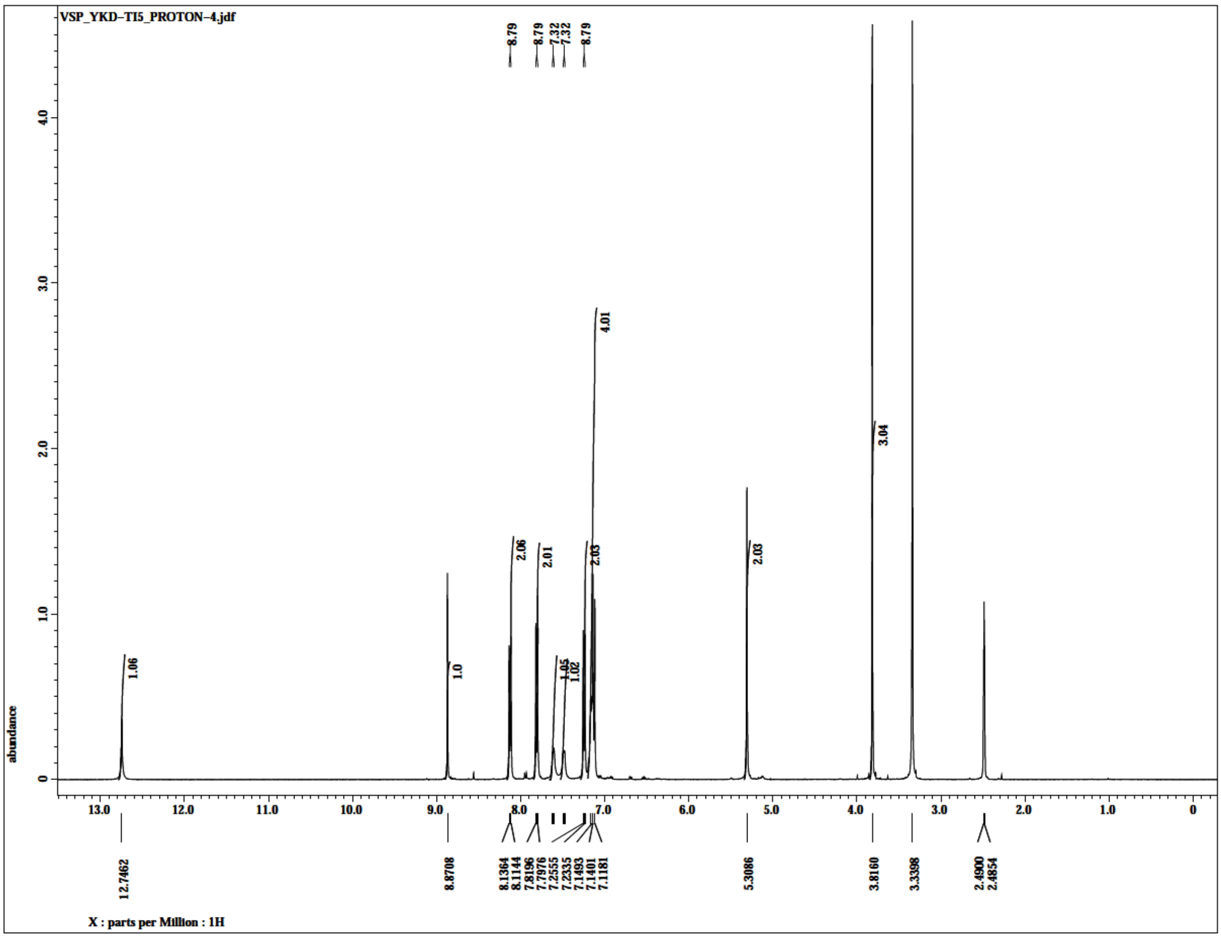
VSP_YKD-TI5_PROTON-4.jdf
abundance
X : parts per Million : 1H
1.06
1.0
2.06
2.01
1.05
1.02
2.03
4.01
2.03
3.04
12.7462
8.8708
8.1364
8.1144
7.8196
7.7976
7.2555
7.2335
7.1493
7.1401
7.1181
5.3086
3.8160
3.3398
2.4900
2.4854

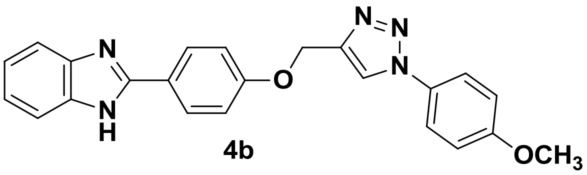

4b

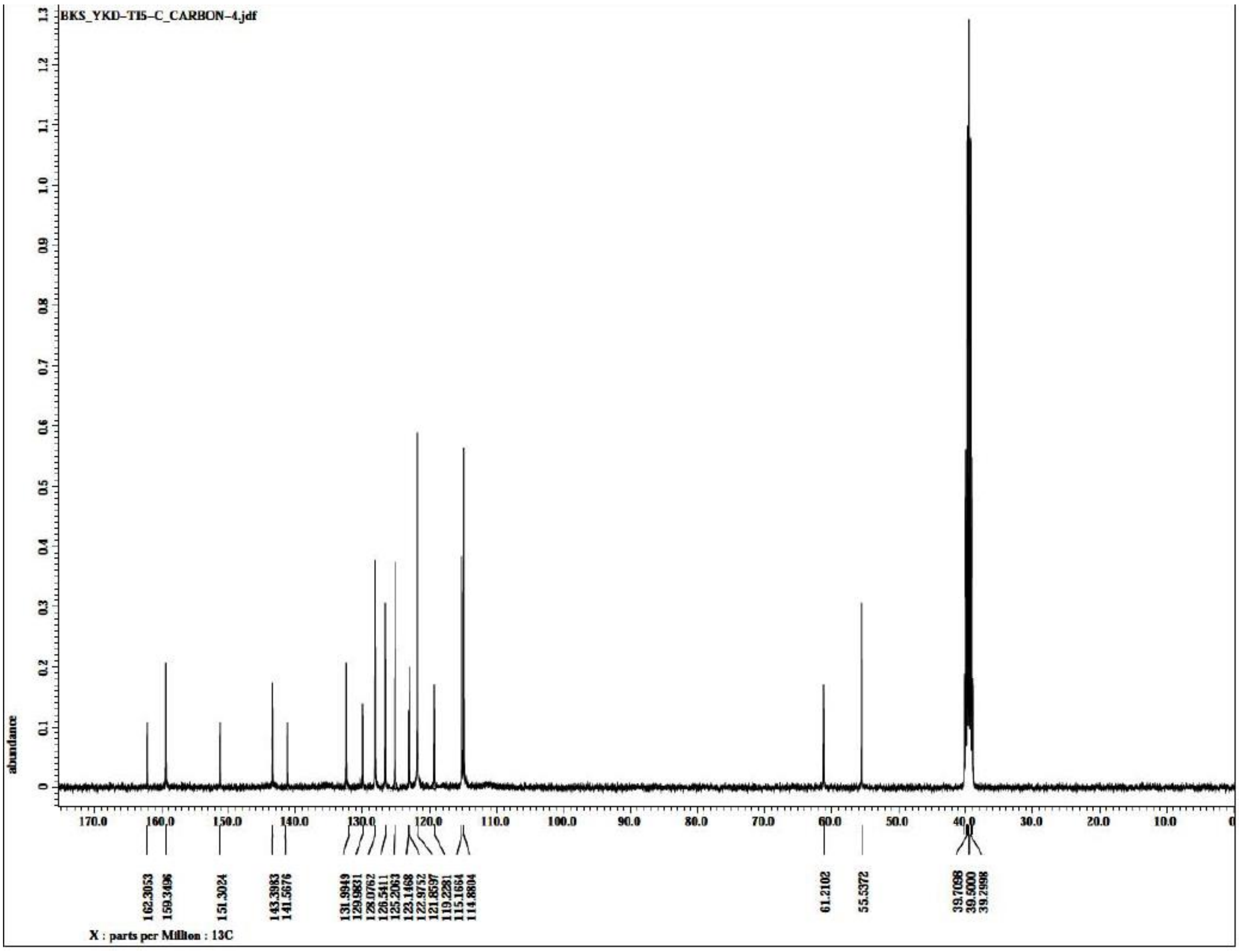
BKS_YKD-T15-C_CARBON-4.jdf
abundance
X : parts per Million : 13C
162.3053
159.3496
151.3024
143.3983
141.5676
131.9949
129.9831
128.0762
126.5411
125.2063
123.1468
122.9752
121.8597
119.2281
115.1664
114.8804
61.2102
55.5372
39.7098
39.5000
39.2998

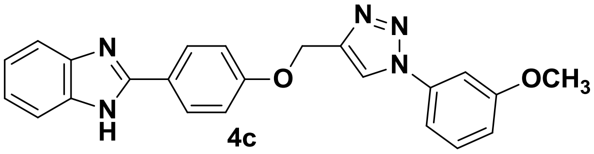
N
N
H
O
N
N
N
OCH3

4c

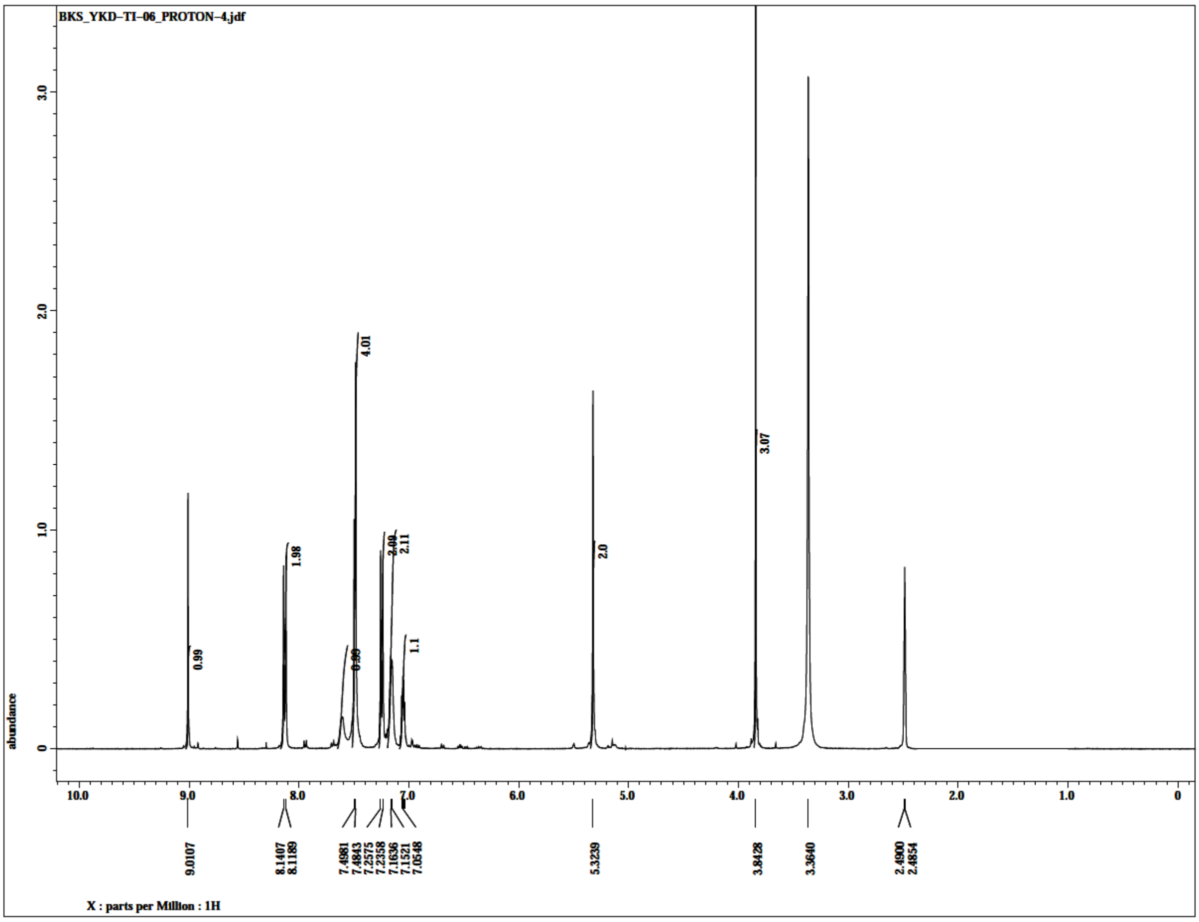
BKS_YKD–TI–06_PROTON–4.jdf
abundance
X : parts per Million : 1H
0.99
1.98
0.90
4.01
2.09
2.11
1.1
2.0
3.07
9.0107
8.1407
8.1189
7.4981
7.4843
7.2575
7.2358
7.1636
7.1521
7.0548
5.3239
3.8428
3.3640
2.4900
2.4854

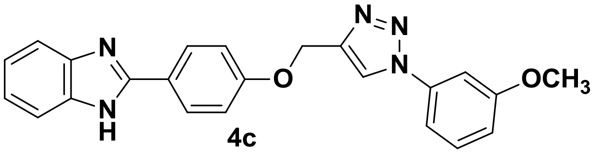

4c

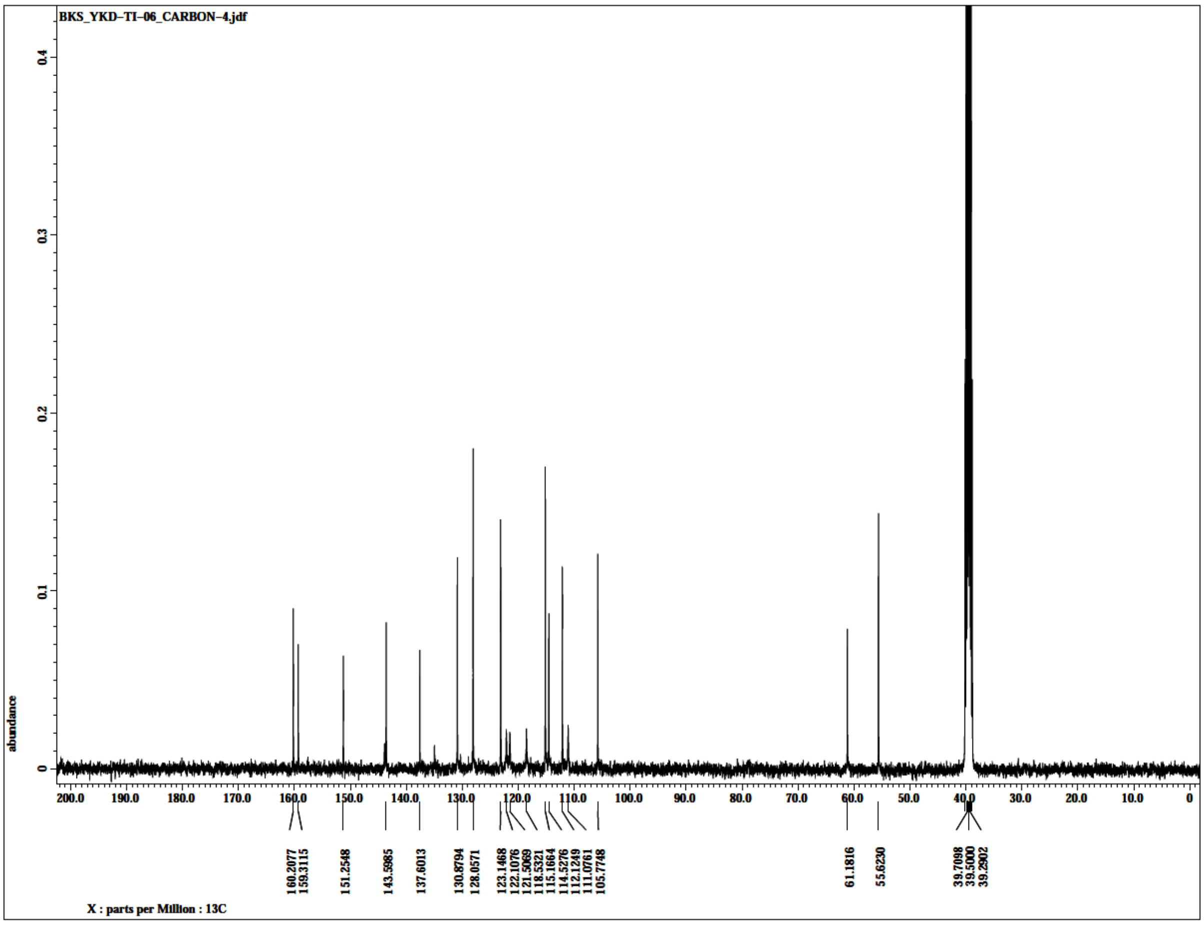

BKS_YKD–TI–06_CARBON–4.jdf
abundance
0
0.1
0.2
0.3
0.4
200.0 190.0 180.0 170.0 160.0 150.0 140.0 130.0 120.0 110.0 100.0 90.0 80.0 70.0 60.0 50.0 40.0 30.0 20.0 10.0 0
160.2077
159.3115
151.2548
143.5985
137.6013
130.8794
128.0571
123.1468
122.1076
121.5069
118.5321
115.1664
114.5276
112.1249
111.0761
105.7748
61.1816
55.6230
39.7098
39.5000
39.2902
X : parts per Million : 13C

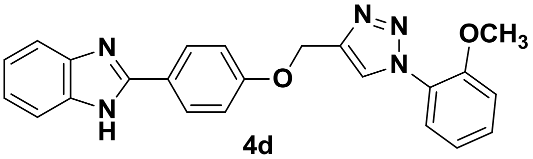
N
N
H
O
N
N
N
OCH3

4d

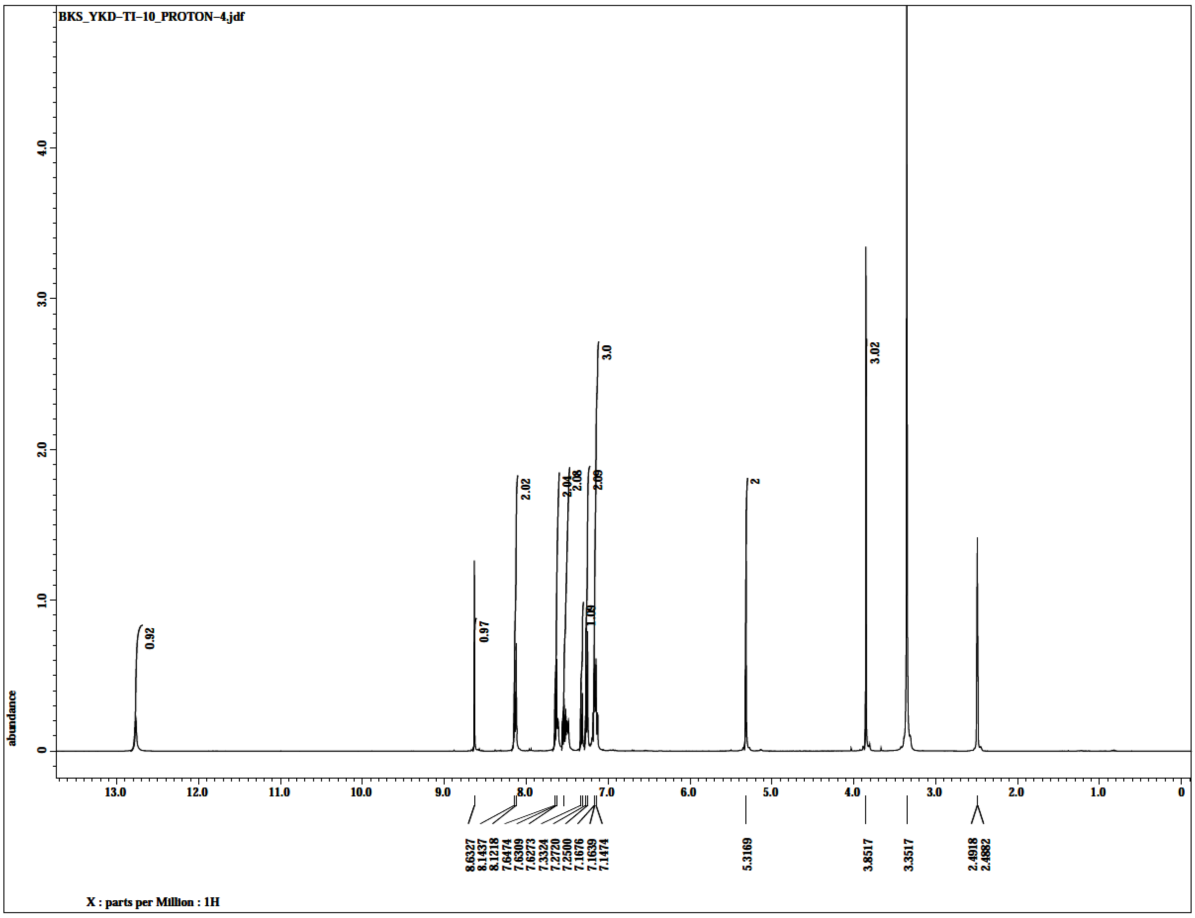
BKS_YKD–TI–10_PROTON–4.jdf
abundance
0.92
0.97
2.02
2.04
2.08
2.09
3.0
2
3.02
8.6327
8.1437
8.1218
7.6474
7.6309
7.6273
7.3324
7.2720
7.2500
7.1676
7.1639
7.1474
5.3169
3.8517
3.3517
2.4918
2.4882
X : parts per Million : 1H

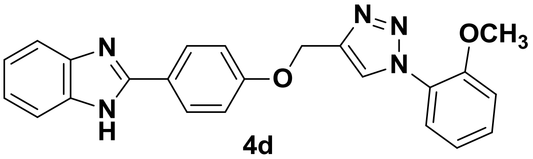

4d

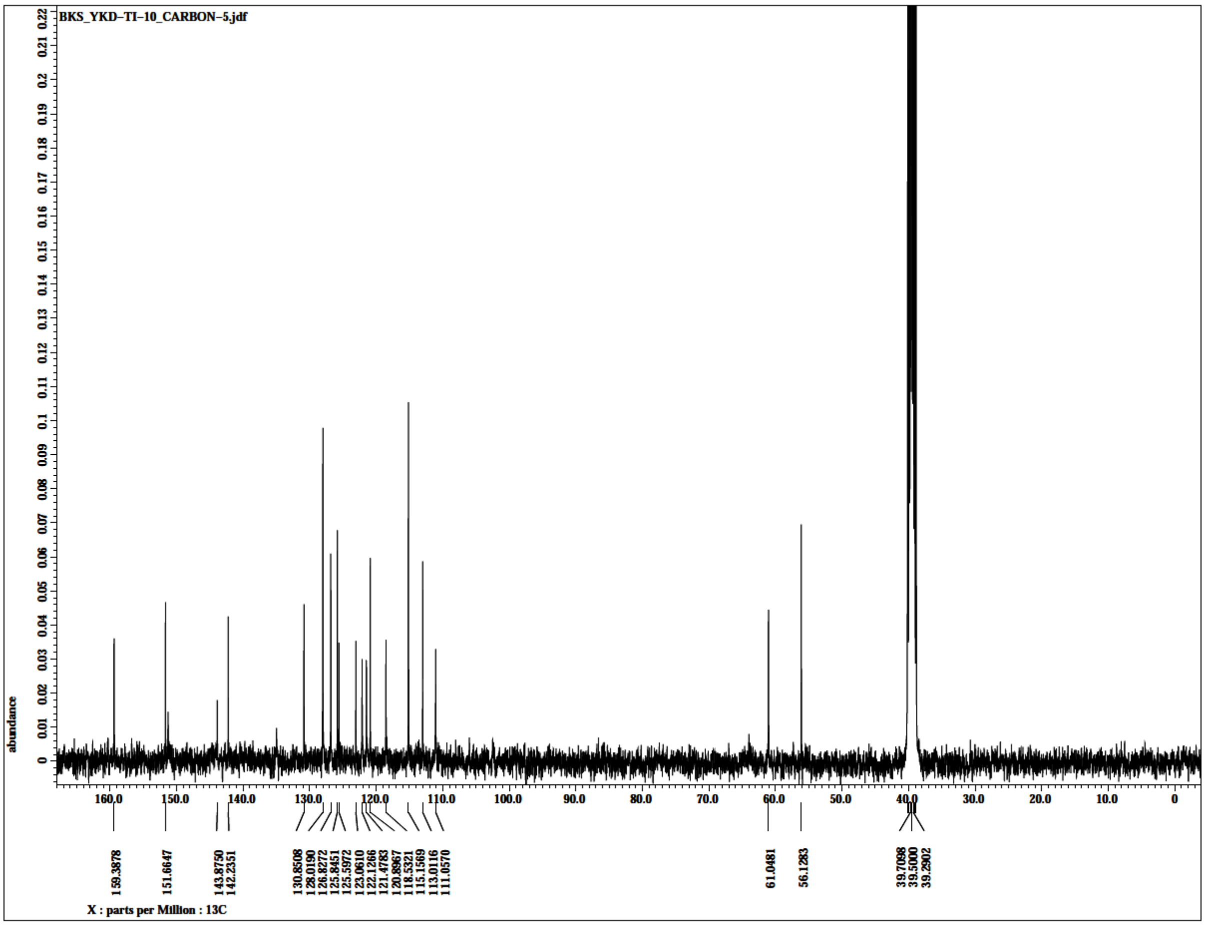
BKS_YKD-TI-10_CARBON-5.jdf
abundance
X : parts per Million : 13C
159.3878
151.6647
143.8750
142.2351
130.8508
128.0190
126.8272
125.8451
125.5972
123.0610
122.1266
121.4783
120.8967
118.5321
115.1569
113.0116
111.0570
61.0481
56.1283
39.7098
39.5000
39.2902

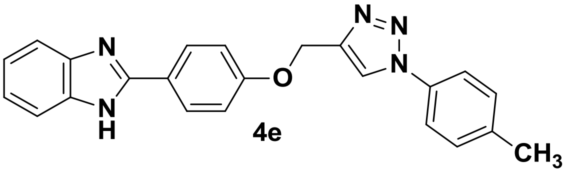

4e

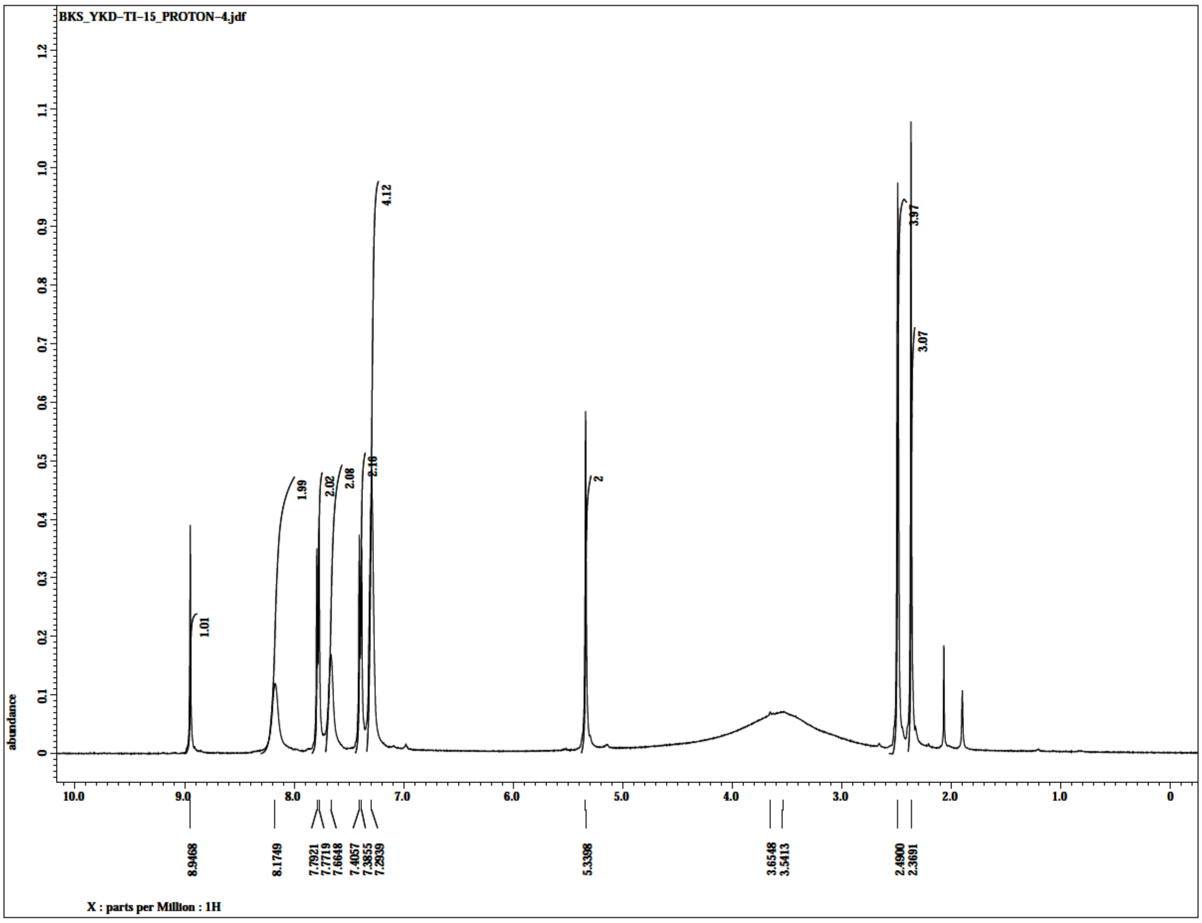
BKS_YKD–TI–15_PROTON–4.jdf
abundance
X : parts per Million : 1H
1.01
1.99
2.02
2.08
2.10
4.12
2
3.97
3.07
8.9468
8.1749
7.7921
7.7719
7.6648
7.4057
7.3855
7.2939
5.3398
3.6548
3.5413
2.4900
2.3691

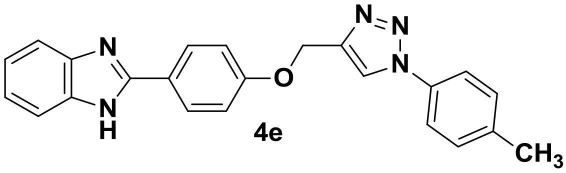

4e

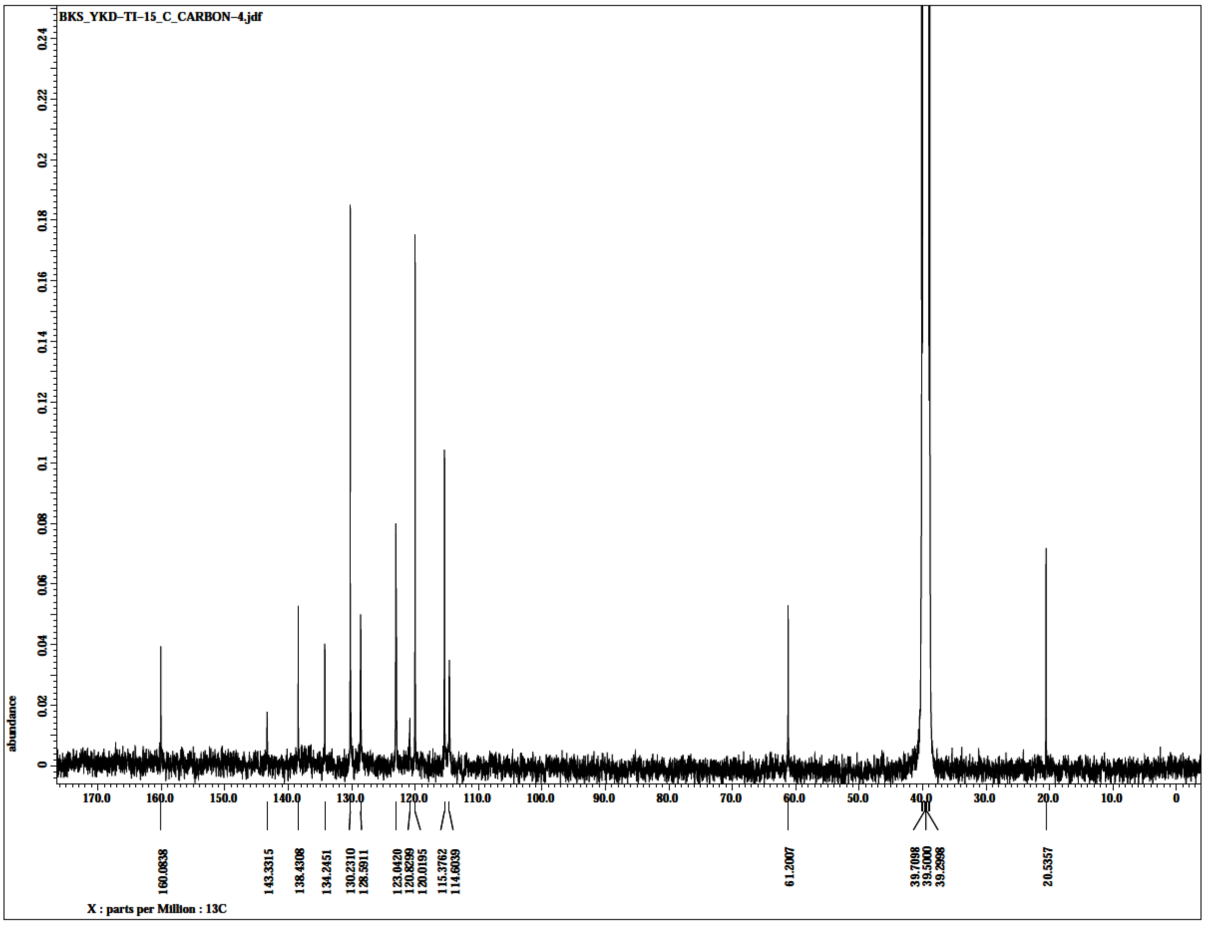

BKS_YKD-TI-15_C_CARBON-4.jdf
abundance
X : parts per Million : 13C
160.0838
143.3315
138.4308
134.2451
130.2310
128.5911
123.0420
120.8299
120.0195
115.3762
114.6039
61.2007
39.7098
39.5000
39.2998
20.5357

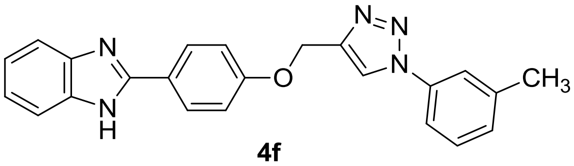
N
N
H
O
N
N
N
CH3

4f

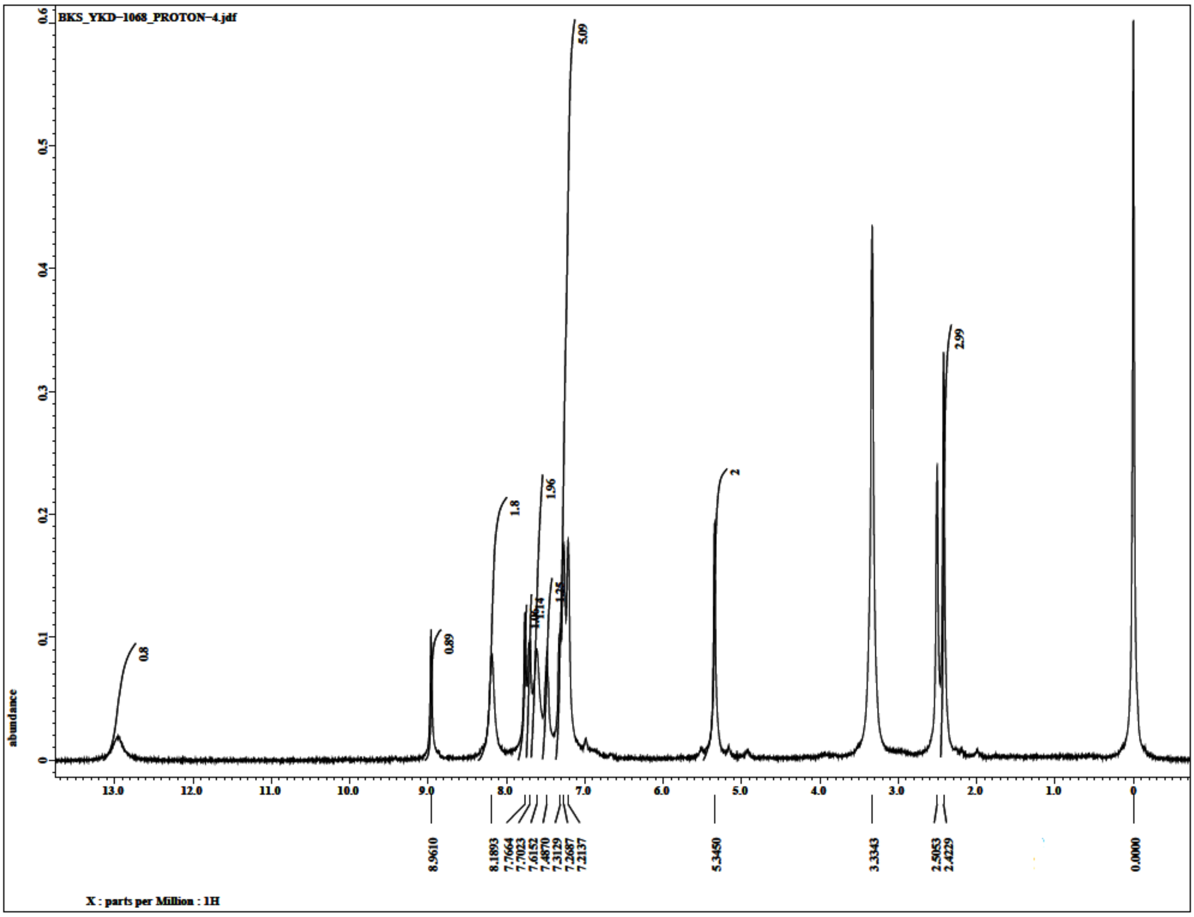
BKS_YKD-1068_PROTON-4.jdf
abundance
X : parts per Million : 1H
0.8
0.89
1.8
1.96
5.09
2
2.99
8.9610
8.1893
7.7664
7.7023
7.6152
7.4870
7.3129
7.2687
7.2137
5.3450
3.3343
2.5053
2.4229
0.0000

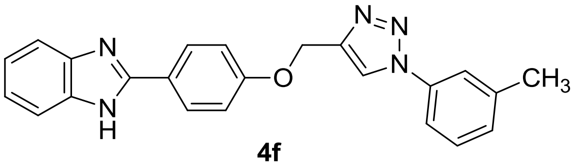

4f

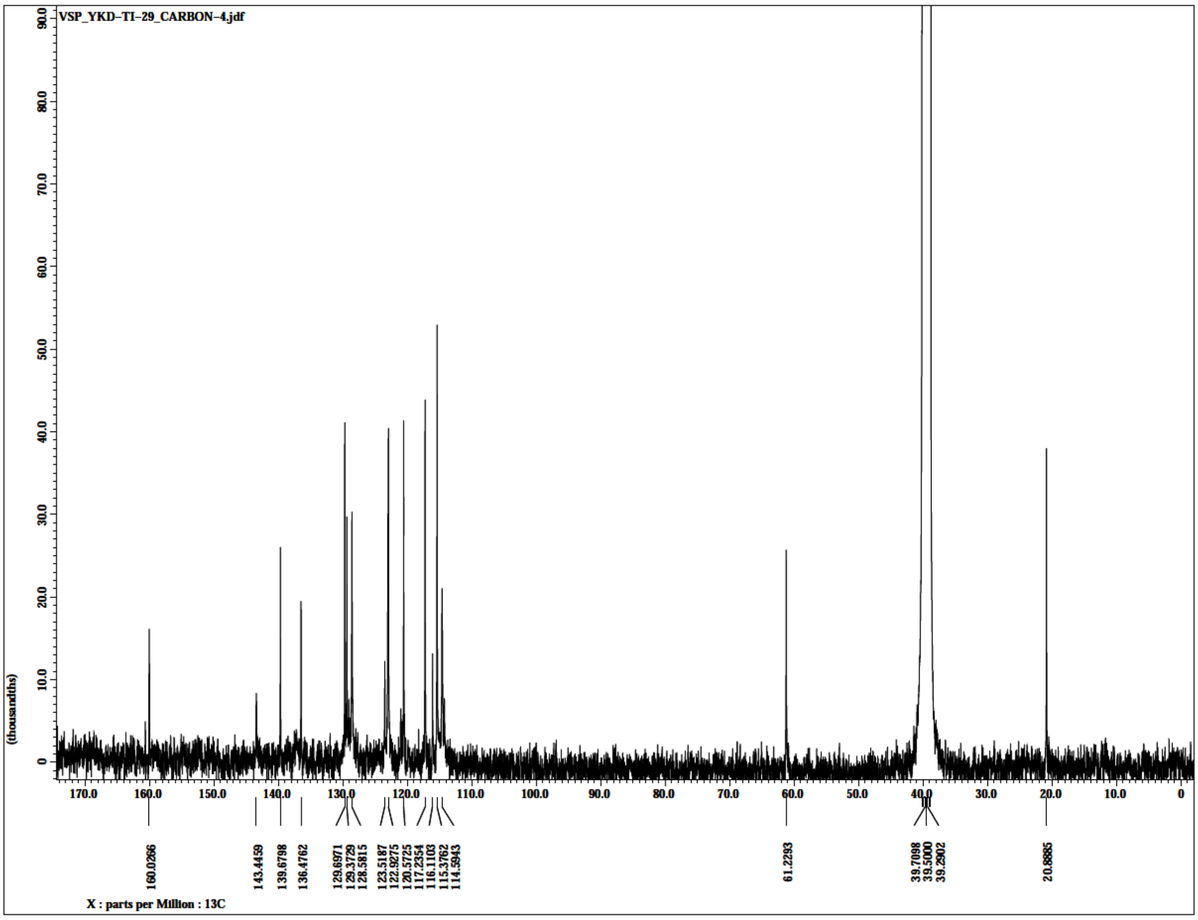
VSP_YKD-TI-29_CARBON-4.jdf
(thousandths)
X : parts per Million : 13C
160.0266
143.4459
139.6798
136.4762
129.6971
129.3729
128.5815
123.5187
122.9275
120.5725
117.2354
116.1103
115.3762
114.5943
61.2293
39.7098
39.5000
39.2902
20.8885

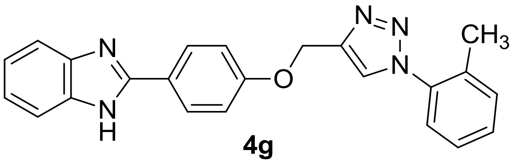

4g

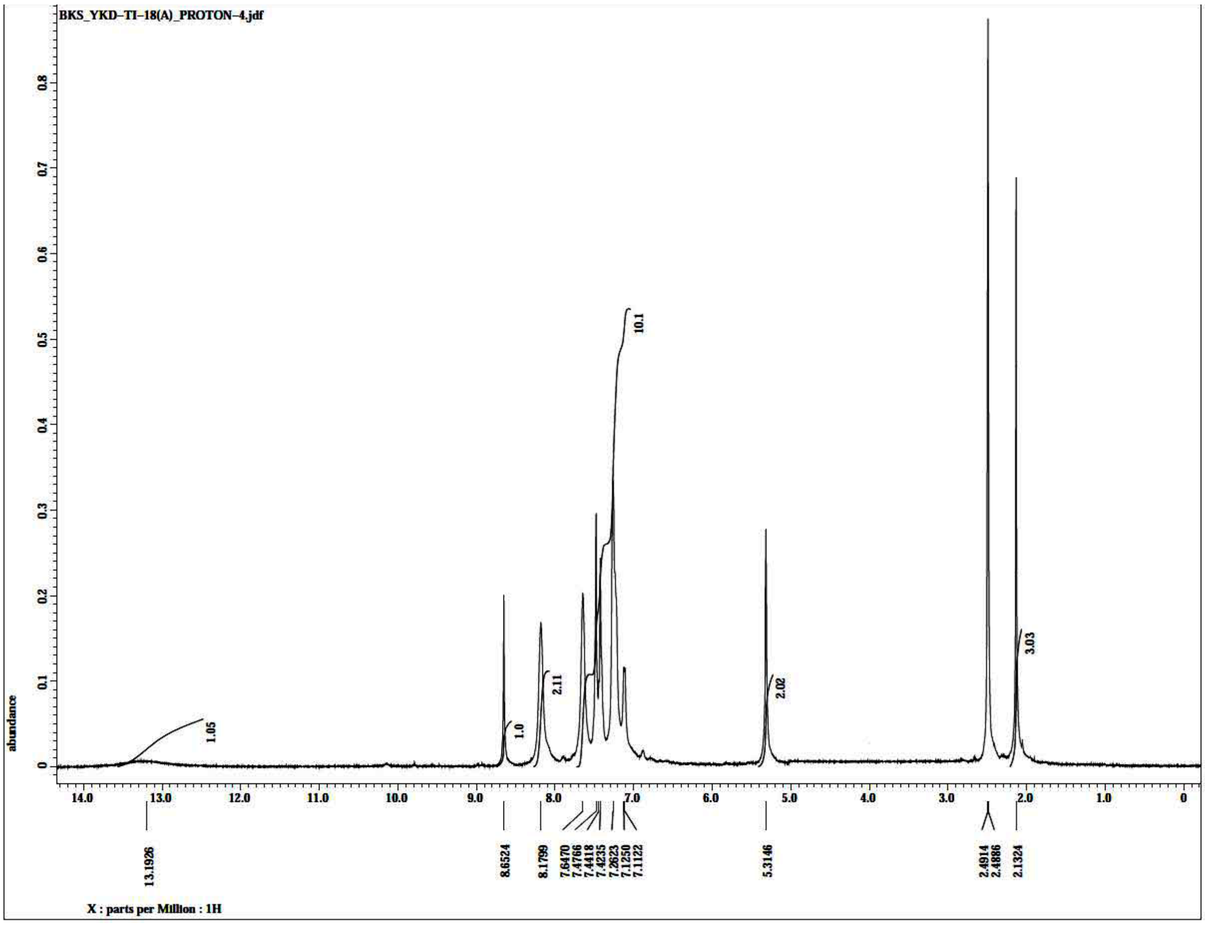

BKS_YKD-TI-18(A)_PROTON-4.jdf
abundance
X : parts per Million : 1H
1.05
1.0
2.11
10.1
2.02
3.03
13.1926
8.6524
8.1799
7.6470
7.4766
7.4418
7.4235
7.2623
7.1250
7.1122
5.3146
2.4914
2.4886
2.1324

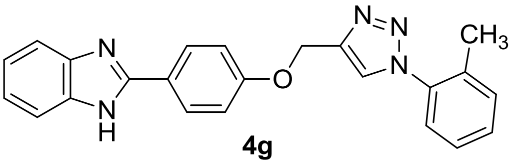

4g

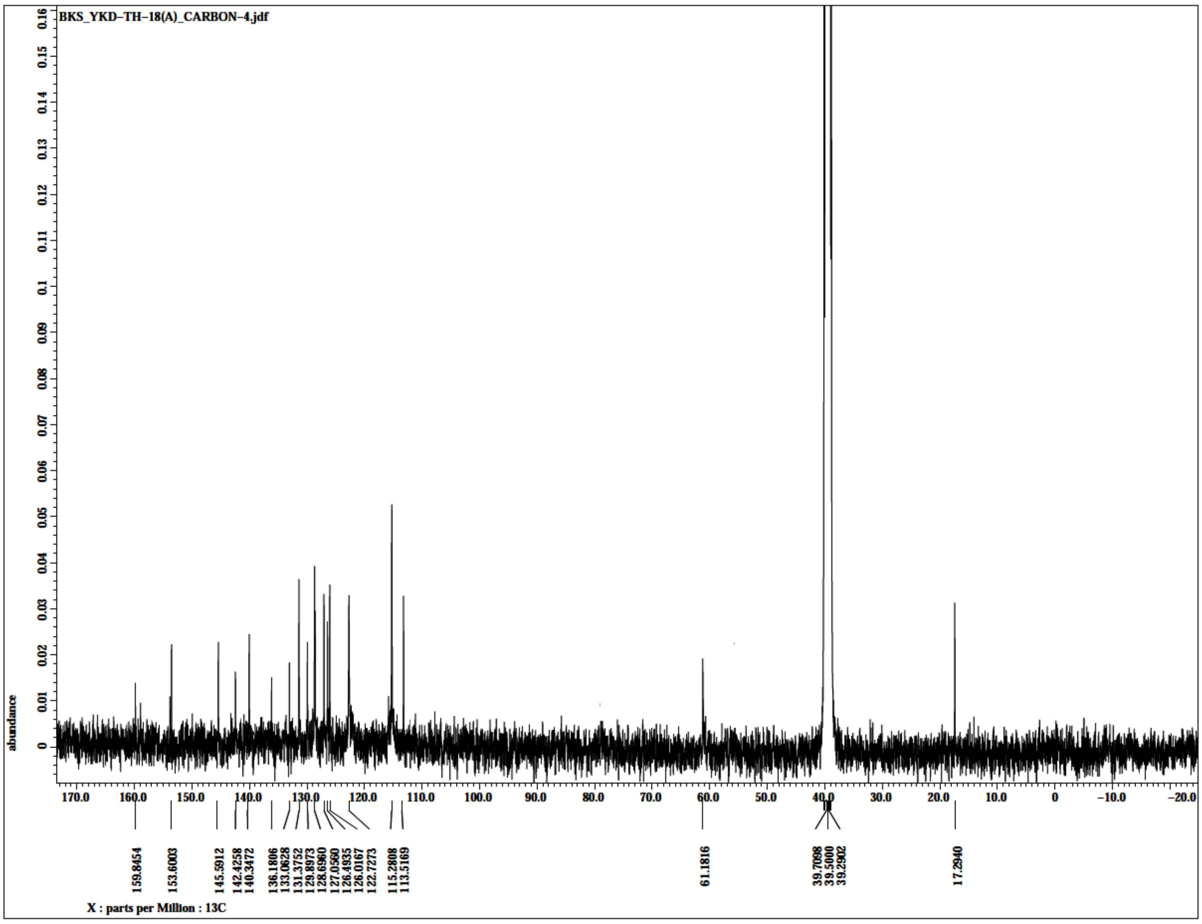

BKS_YKD–TH–18(A)_CARBON–4.jdf
abundance
X : parts per Million : 13C
159.8454
153.6003
145.5912
142.4258
140.3472
136.1806
133.0628
131.3752
129.8973
128.6960
127.0560
126.4935
126.0167
122.7273
115.2808
113.5169
61.1816
39.7098
39.5000
39.2902
17.2940

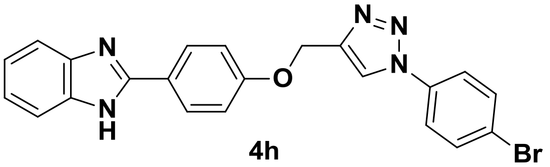

4h

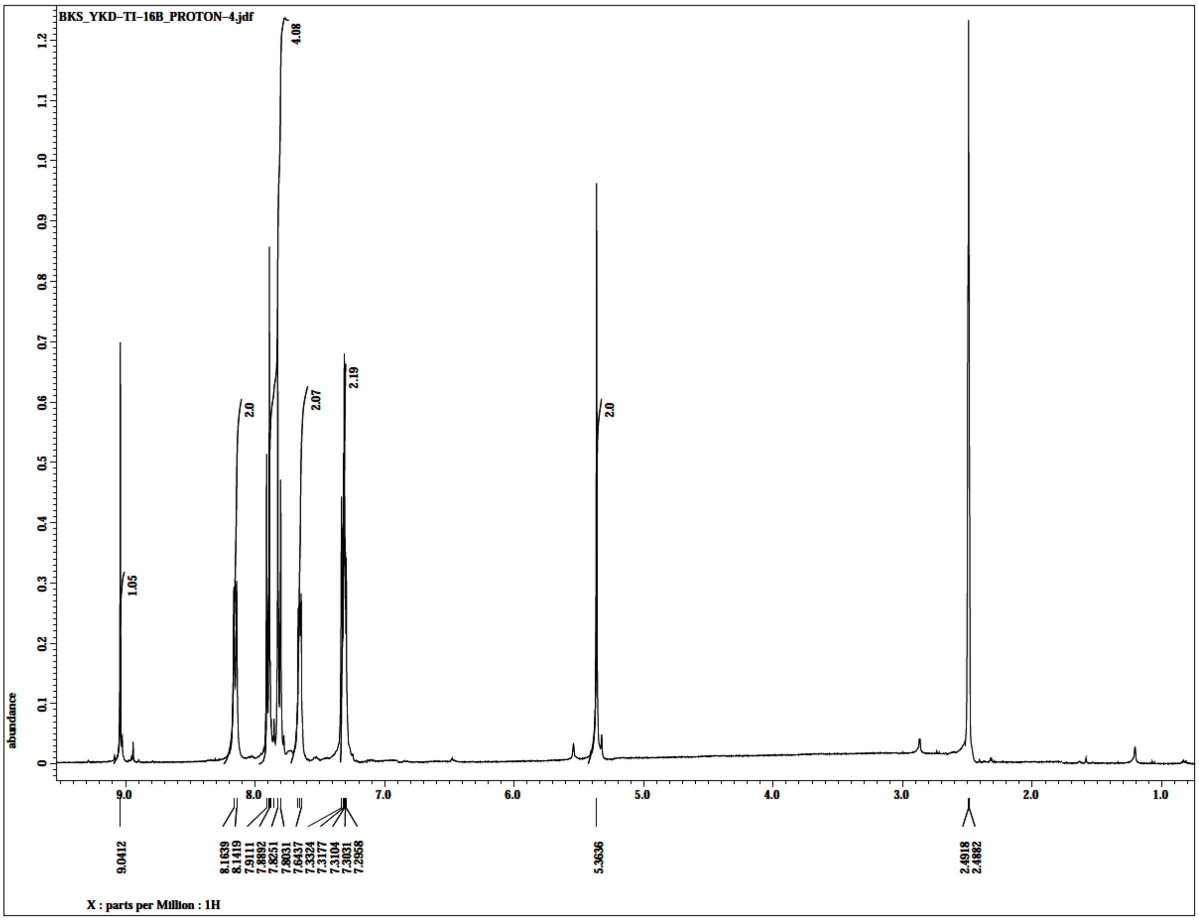

BKS_YKD–TI–16B_PROTON–4.jdf
abundance
X : parts per Million : 1H
1.05
2.0
4.08
2.07
2.19
2.0
9.0412
8.1639
8.1419
7.9111
7.8892
7.8251
7.8031
7.6437
7.3324
7.3177
7.3104
7.3031
7.2958
5.3636
2.4918
2.4882

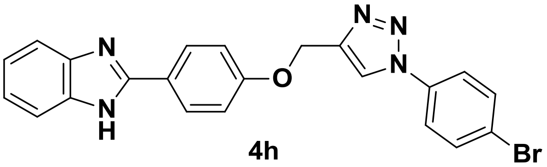

4h

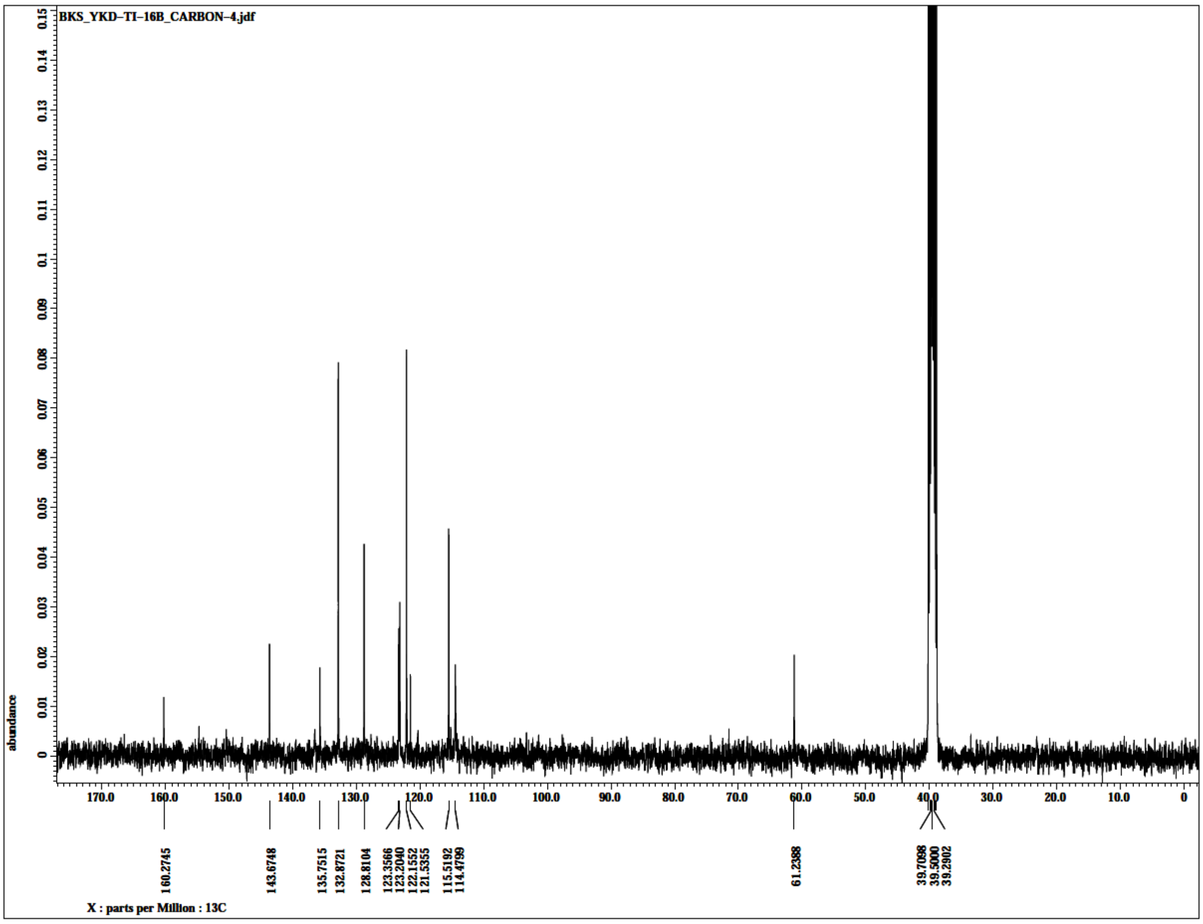

BKS_YKD-TI-16B_CARBON-4.jdf
abundance
0.15
0.14
0.13
0.12
0.11
0.1
0.09
0.08
0.07
0.06
0.05
0.04
0.03
0.02
0.01
0
170.0
160.0
150.0
140.0
130.0
120.0
110.0
100.0
90.0
80.0
70.0
60.0
50.0
40.0
30.0
20.0
10.0
0
160.2745
143.6748
135.7515
132.8721
128.8104
123.3566
123.2040
122.1552
121.5355
115.5192
114.4799
61.2388
39.7098
39.5000
39.2902
X : parts per Million : 13C

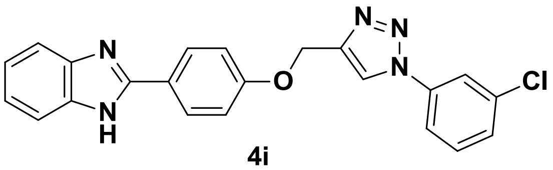
N
H
O
N
N
N
Cl

4i

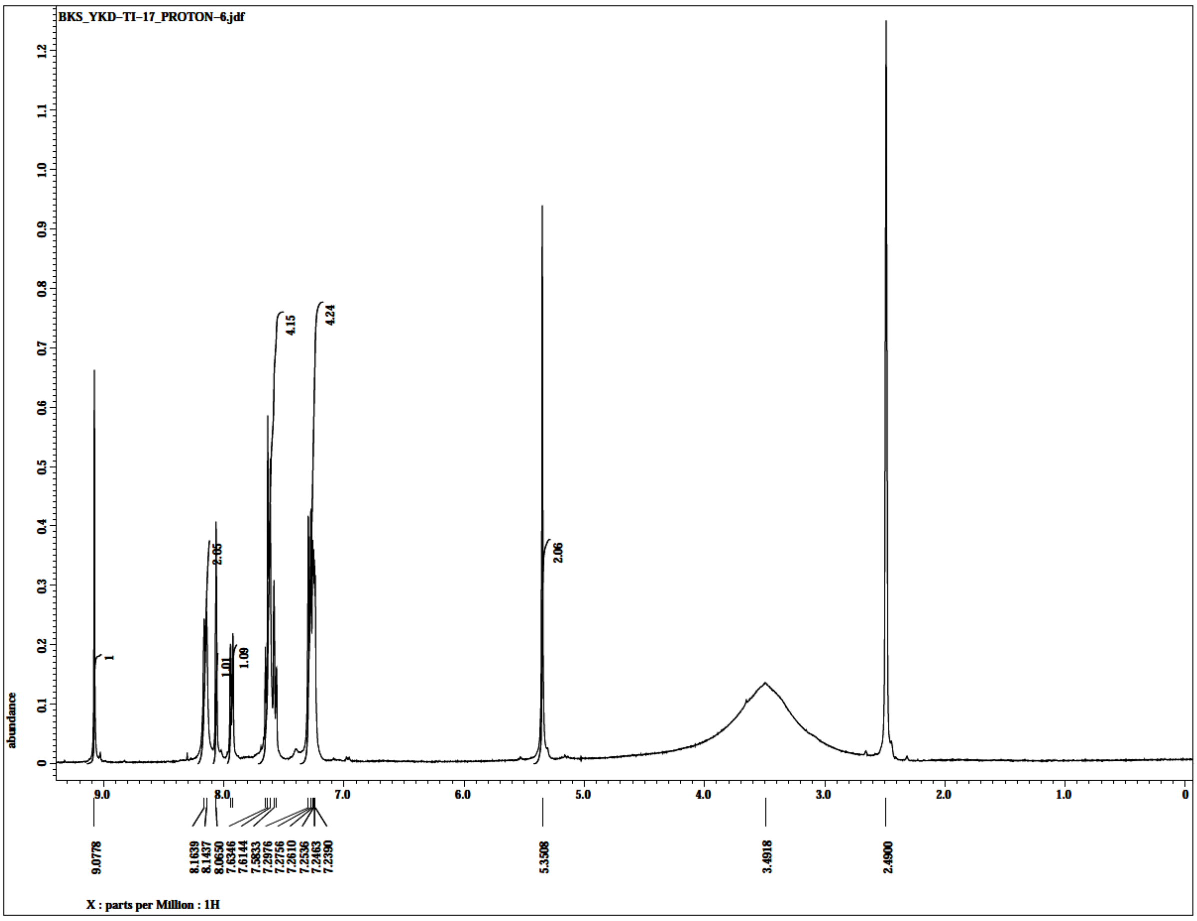
BKS_YKD-TI-17_PROTON-6.jdf
abundance
0
0.1
0.2
0.3
0.4
0.5
0.6
0.7
0.8
0.9
1.0
1.1
1.2
1
2.05
1.01
1.09
4.15
4.24
2.06
9.0
8.0
7.0
6.0
5.0
4.0
3.0
2.0
1.0
0
9.0778
8.1639
8.1437
8.0650
7.6346
7.6144
7.5833
7.2976
7.2756
7.2610
7.2536
7.2463
7.2390
5.3508
3.4918
2.4900
X : parts per Million : 1H

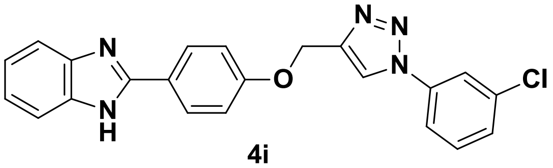

4i

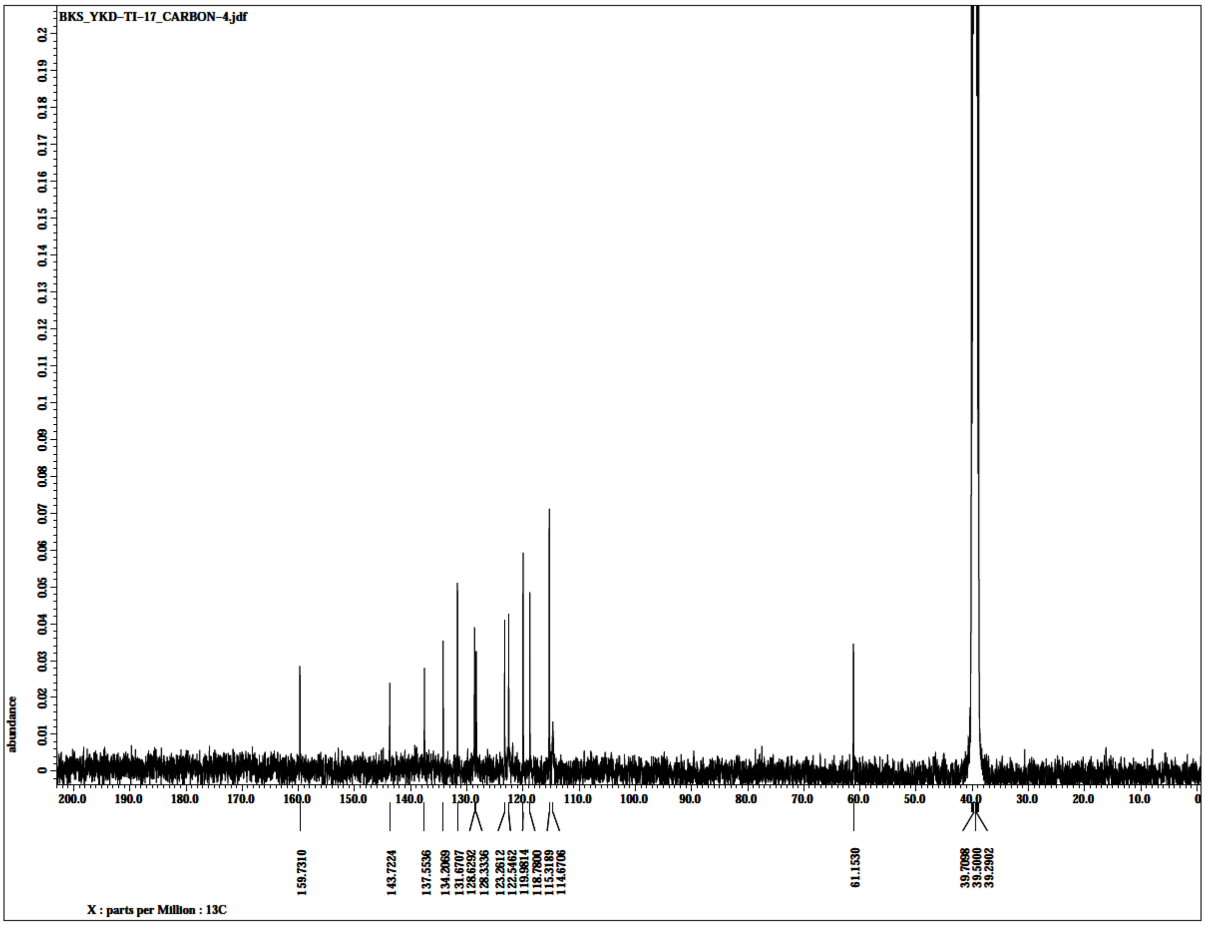

BKS_YKD–TI–17_CARBON–4.jdf
abundance
0
0.01
0.02
0.03
0.04
0.05
0.06
0.07
0.08
0.09
0.1
0.11
0.12
0.13
0.14
0.15
0.16
0.17
0.18
0.19
0.2
200.0
190.0
180.0
170.0
160.0
150.0
140.0
130.0
120.0
110.0
100.0
90.0
80.0
70.0
60.0
50.0
40.0
30.0
20.0
10.0
0
159.7310
143.7224
137.5536
134.2069
131.6707
128.6292
128.3336
123.2612
122.5462
119.9814
118.7800
115.3189
114.6706
61.1530
39.7098
39.5000
39.2902
X : parts per Million : 13C

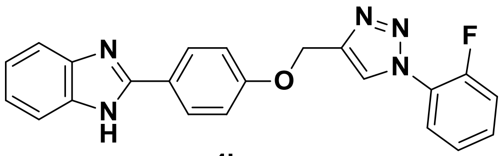
N
N
H
O
N
N
N
F

4j

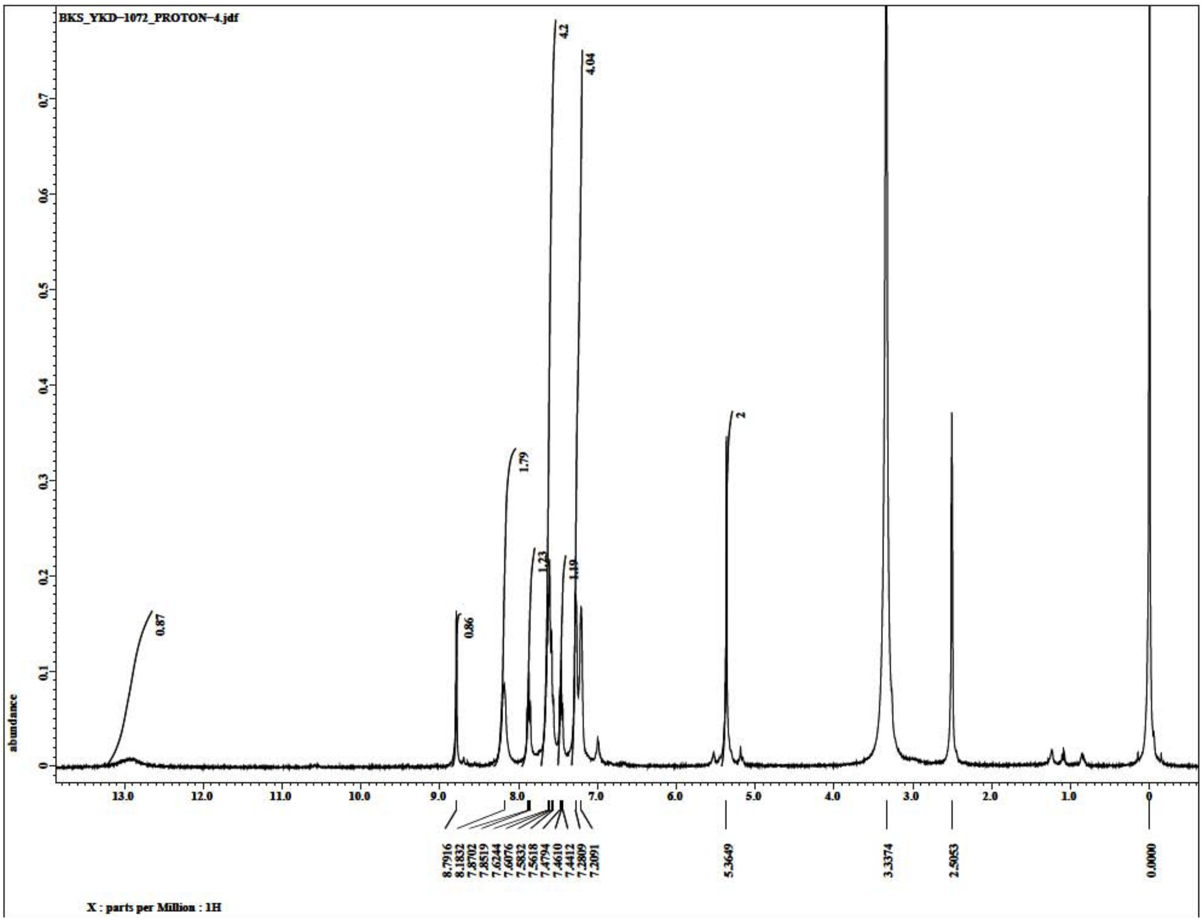
BKS_YKD-1072_PROTON-4.jdf
abundance
0.7
0.6
0.5
0.4
0.3
0.2
0.1
0
0.87
0.86
1.79
1.23
4.2
4.04
1.19
2
13.0
12.0
11.0
10.0
9.0
8.0
7.0
6.0
5.0
4.0
3.0
2.0
1.0
0
8.7916
8.1832
7.8702
7.8519
7.6244
7.6076
7.5832
7.5618
7.4794
7.4610
7.4412
7.2809
7.2091
5.3649
3.3374
2.5053
0.0000
X : parts per Million : 1H

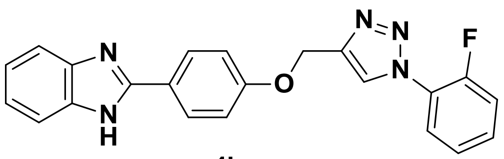

4j

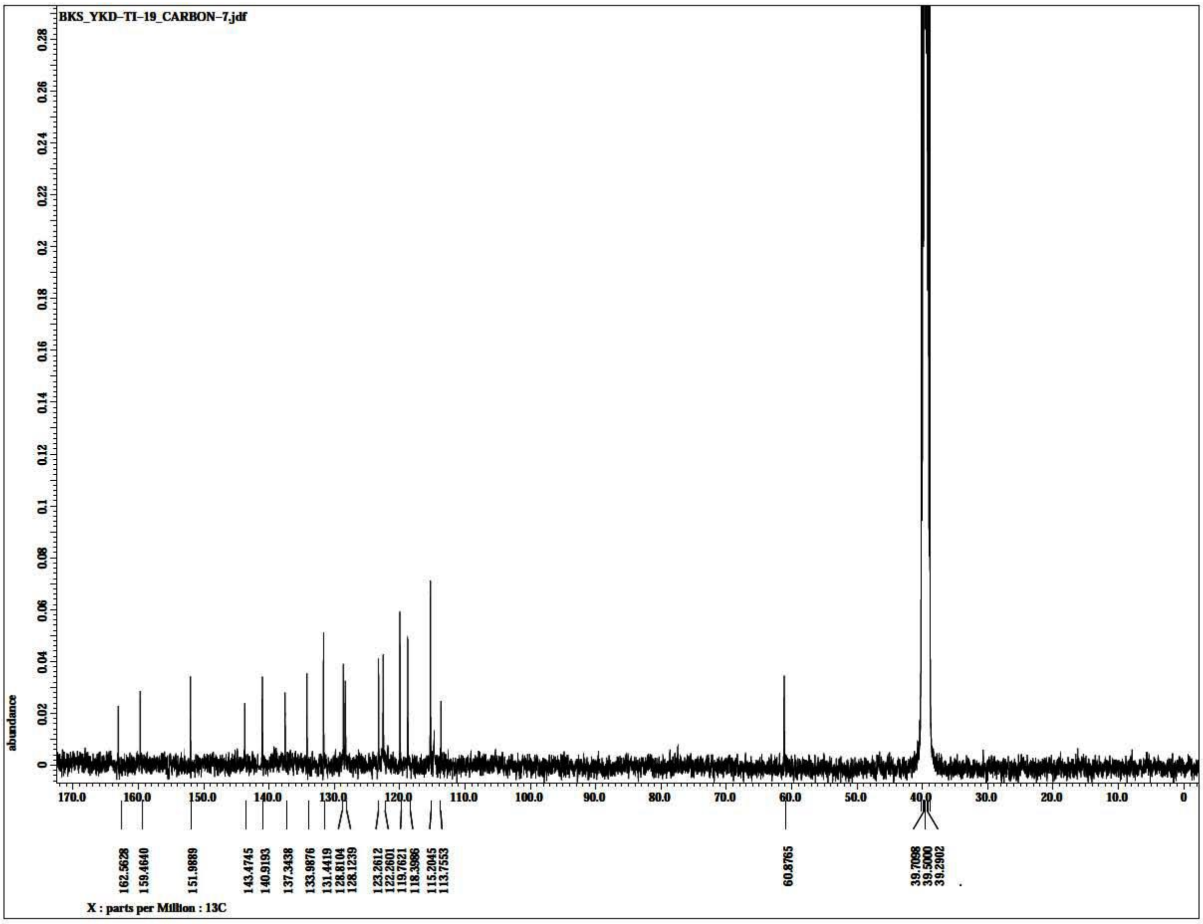
BKS_YKD-TI-19_CARBON-7.jdf
abundance
X : parts per Million : 13C
162.5628
159.4640
151.9889
143.4745
140.9193
137.3438
133.9876
131.4419
128.8104
128.1239
123.2612
122.2601
119.7621
118.3986
115.2045
113.7553
60.8765
39.7098
39.5000
39.2902

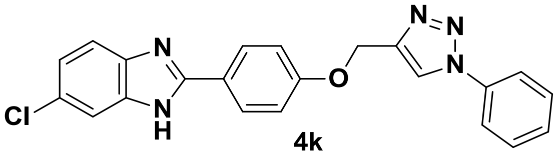

4k

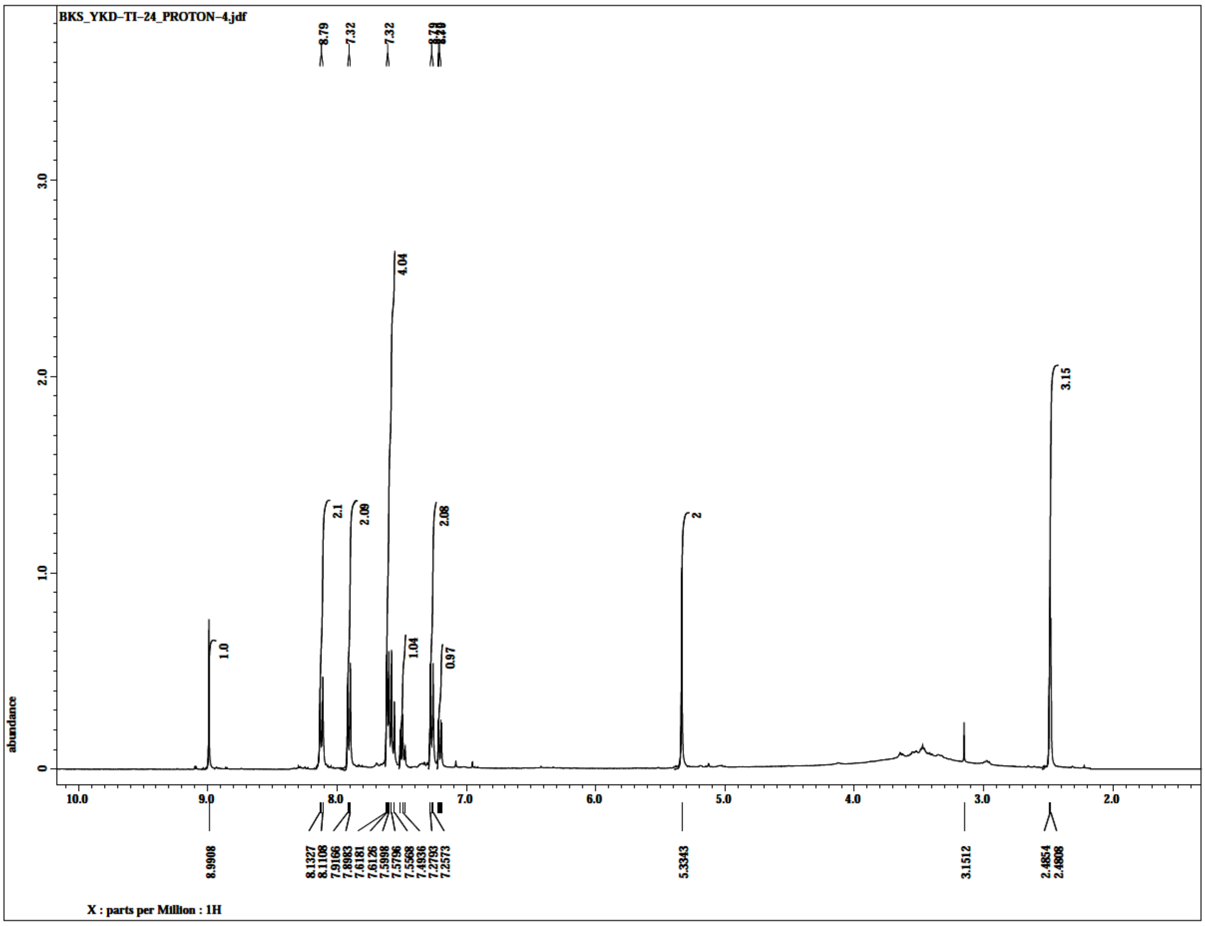
BKS_YKD–TI–24_PROTON–4.jdf
8.79
7.32
7.32
abundance
0
1.0
2.0
3.0
1.0
2.1
2.09
4.04
1.04
2.08
0.97
2
3.15
10.0
9.0
8.0
7.0
6.0
5.0
4.0
3.0
2.0
8.9908
8.1327
8.1108
7.9166
7.8983
7.6181
7.6126
7.5998
7.5796
7.5568
7.4936
7.2793
7.2573
5.3343
3.1512
2.4854
2.4808
X : parts per Million : 1H

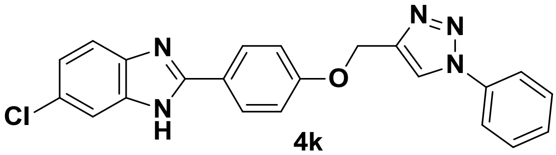

4k

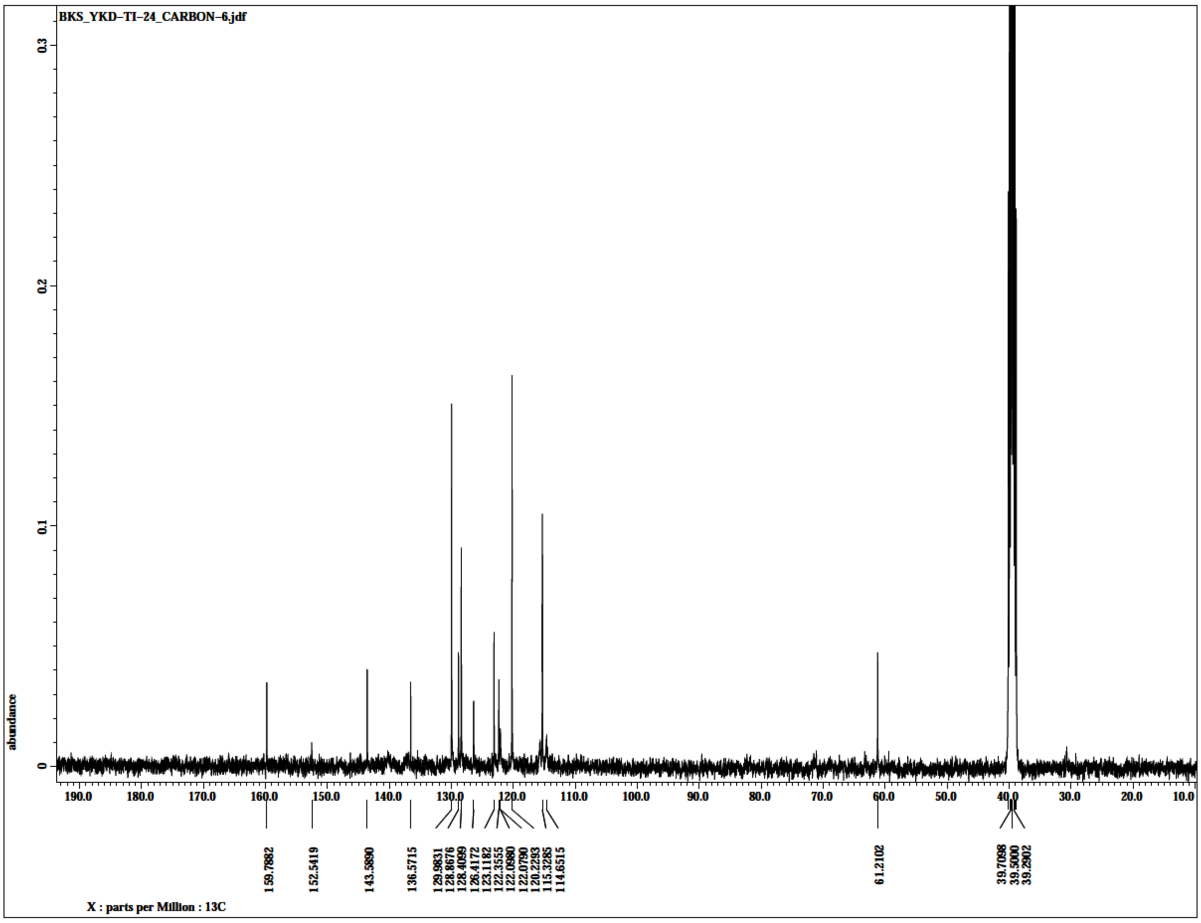
BKS_YKD-TI-24_CARBON-6.jdf
abundance
X : parts per Million : 13C
159.7882
152.5419
143.5890
136.5715
129.9831
128.8676
128.4099
126.4172
123.1182
122.3555
122.0980
122.0790
120.2293
115.3285
114.6515
61.2102
39.7098
39.5000
39.2902

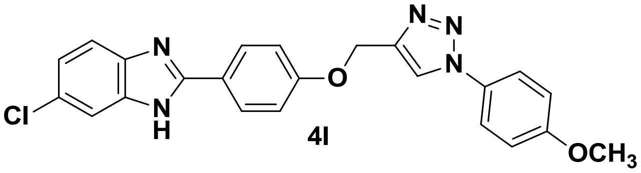

4l

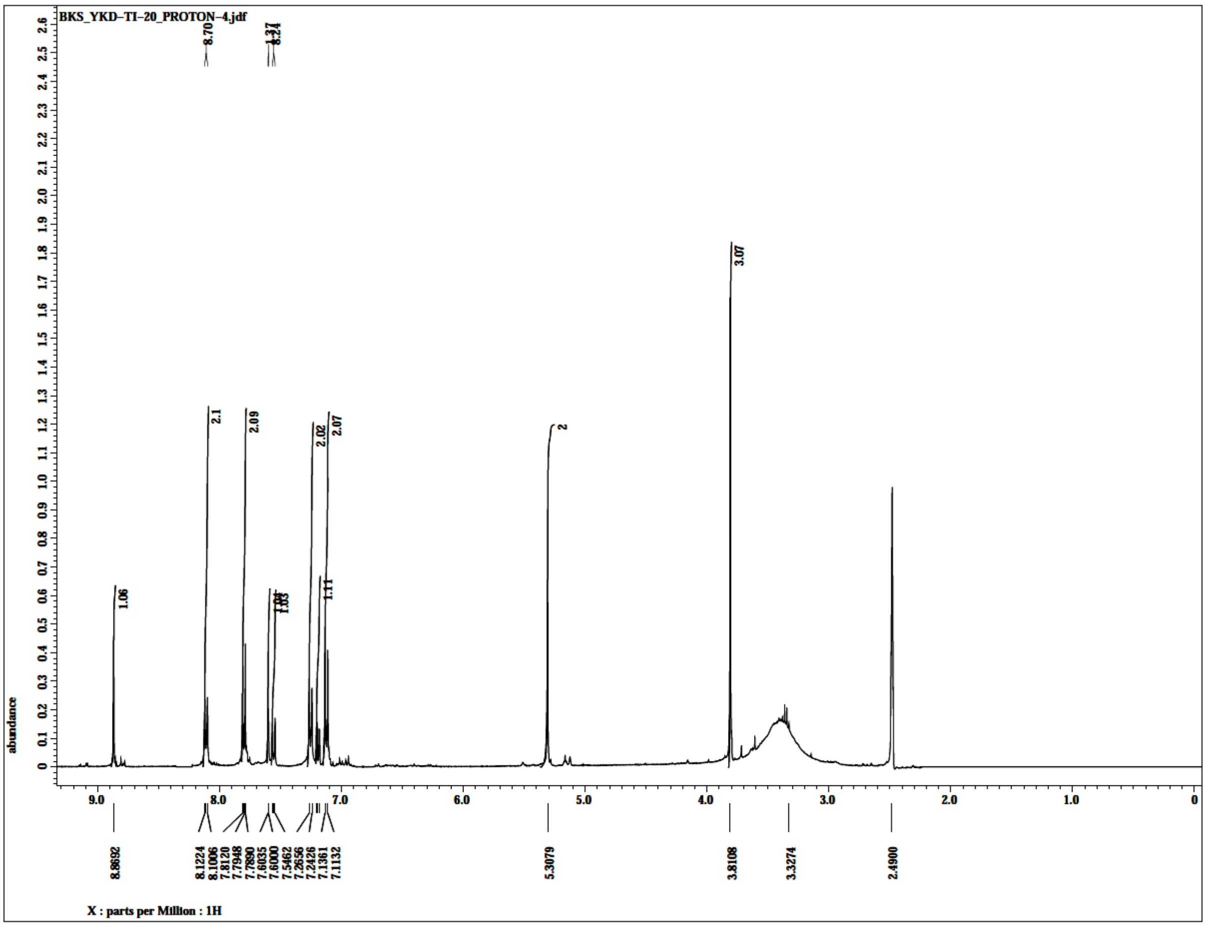

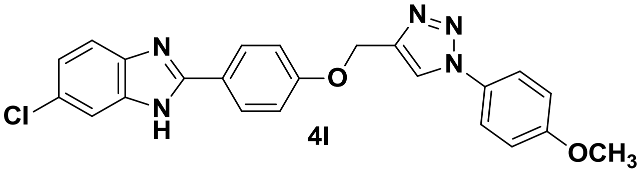
N
N
H
Cl
O
N
N
N
4l
OCH3

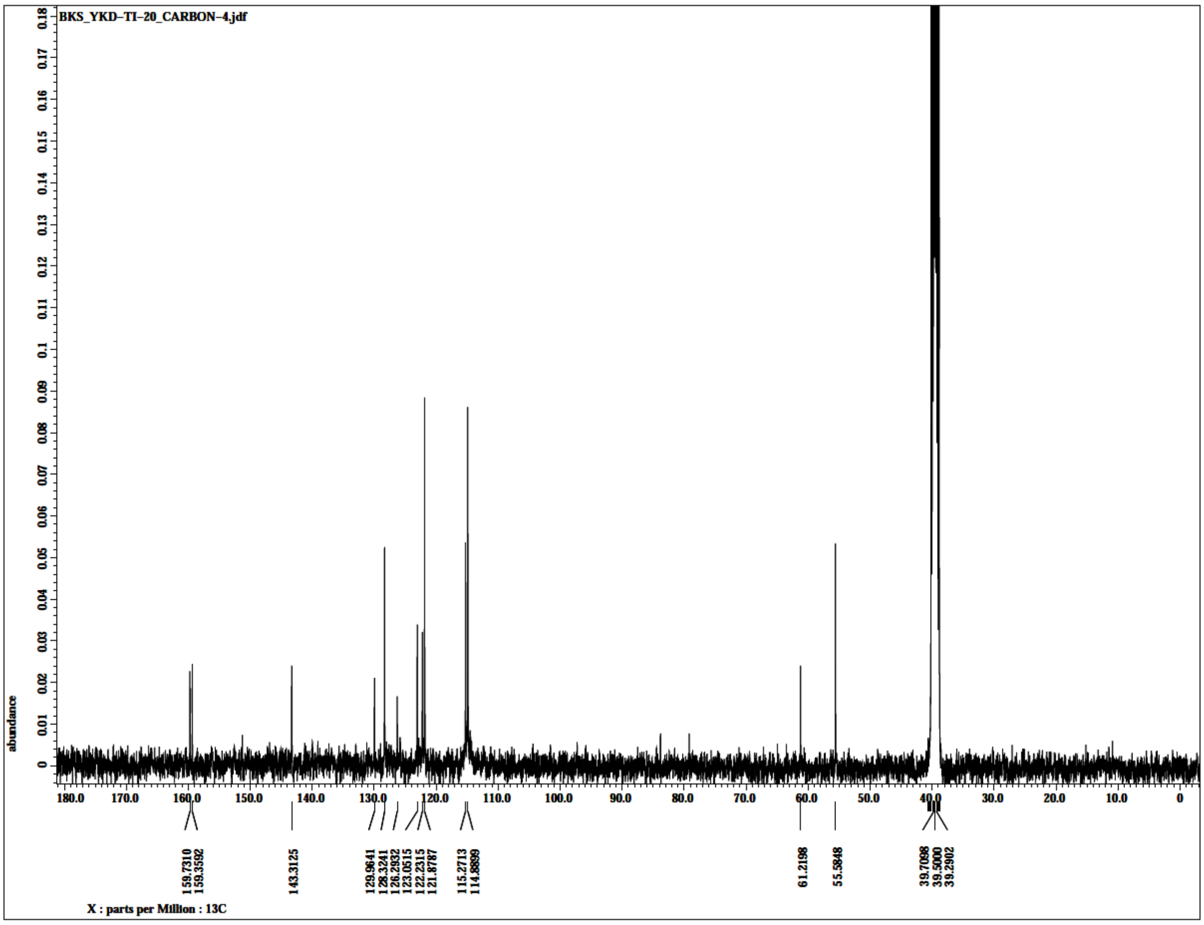
BKS_YKD-TI-20_CARBON-4.jdf
abundance
X : parts per Million : 13C
159.7310
159.3592
143.3125
129.9641
128.3241
126.2932
123.0515
122.2315
121.8787
115.2713
114.8899
61.2198
55.5848
39.7098
39.5000
39.2902

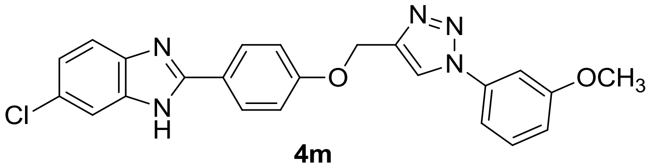

4m

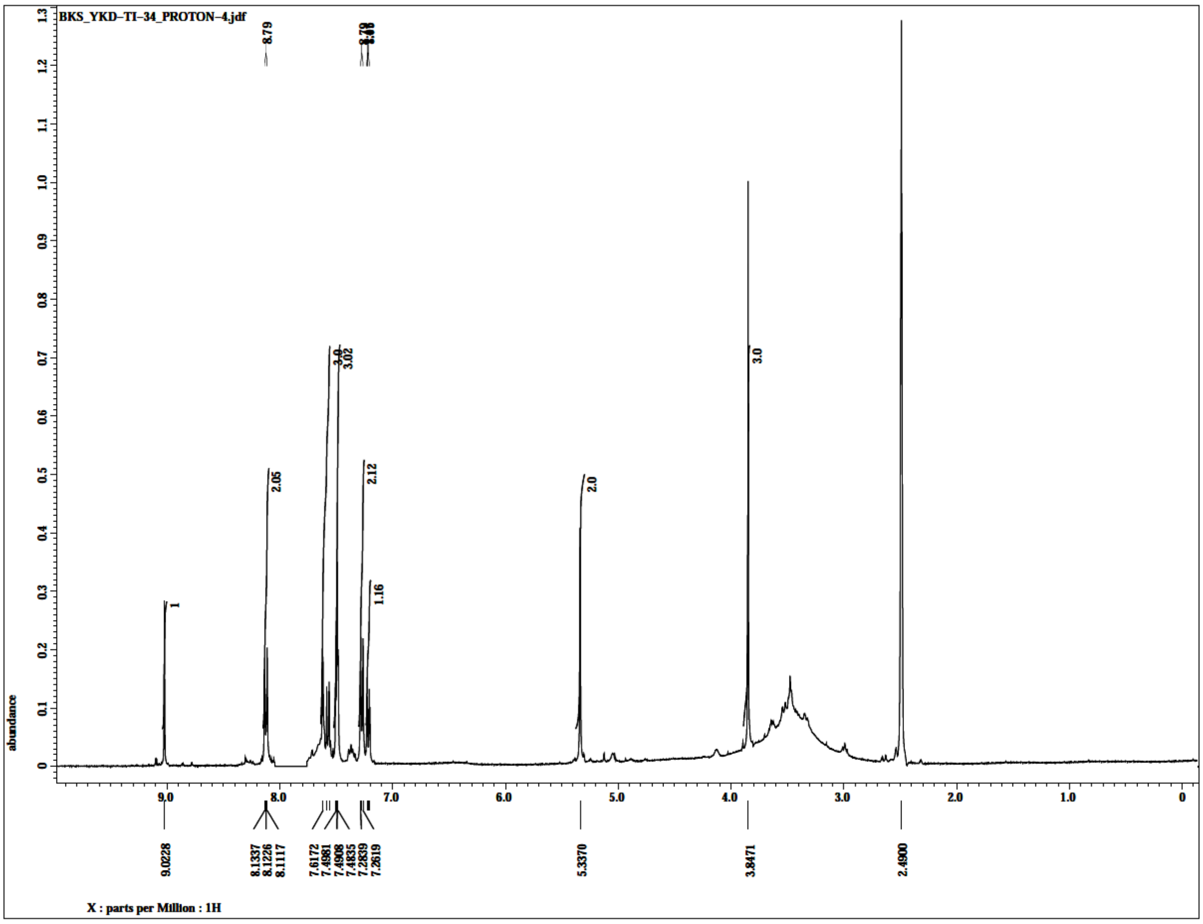
BKS_YKD-TI-34_PROTON-4.jdf
abundance
X : parts per Million : 1H
9.0228
8.1337
8.1226
8.1117
7.6172
7.4981
7.4908
7.4835
7.2839
7.2619
5.3370
3.8471
2.4900

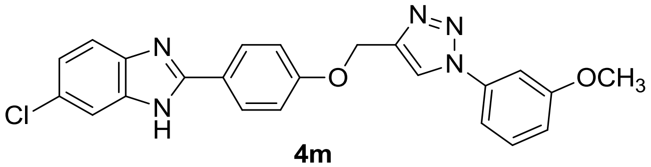

4m

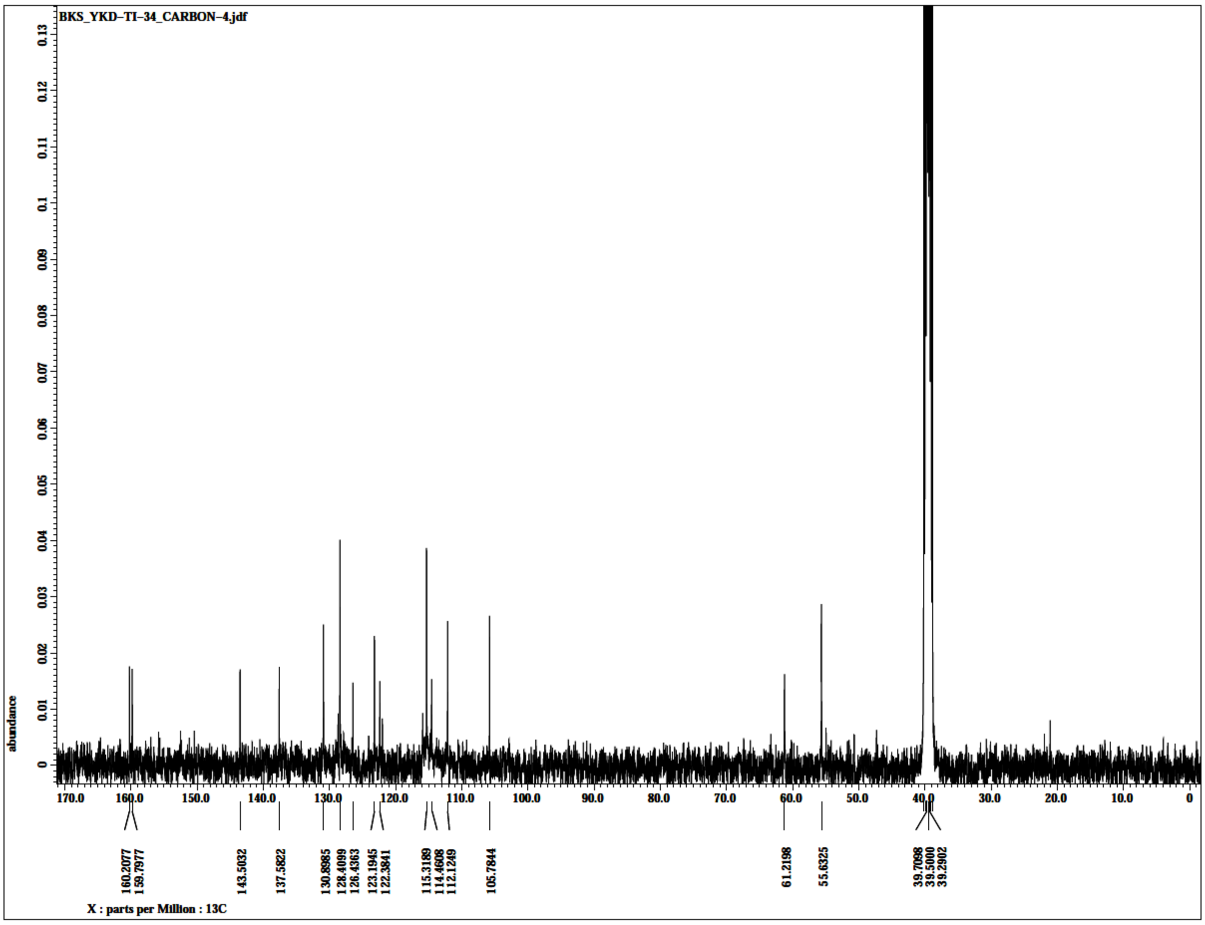
BKS_YKD-TI-34_CARBON-4.jdf
abundance
160.2077
159.7977
143.5032
137.5822
130.8985
128.4099
126.4363
123.1945
122.3841
115.3189
114.4608
112.1249
105.7844
61.2198
55.6325
39.7098
39.5000
39.2902
X : parts per Million : 13C

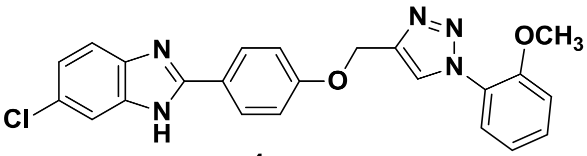

4n

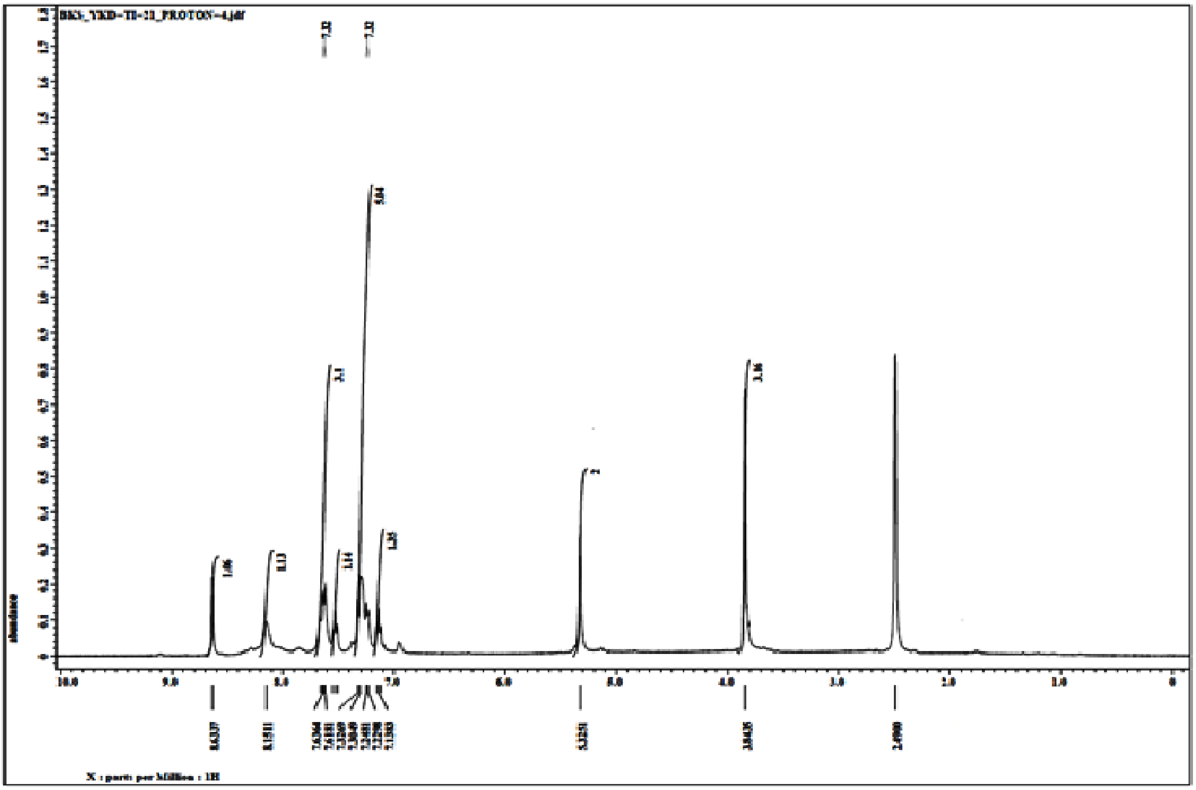

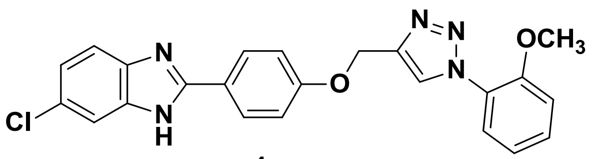

4n

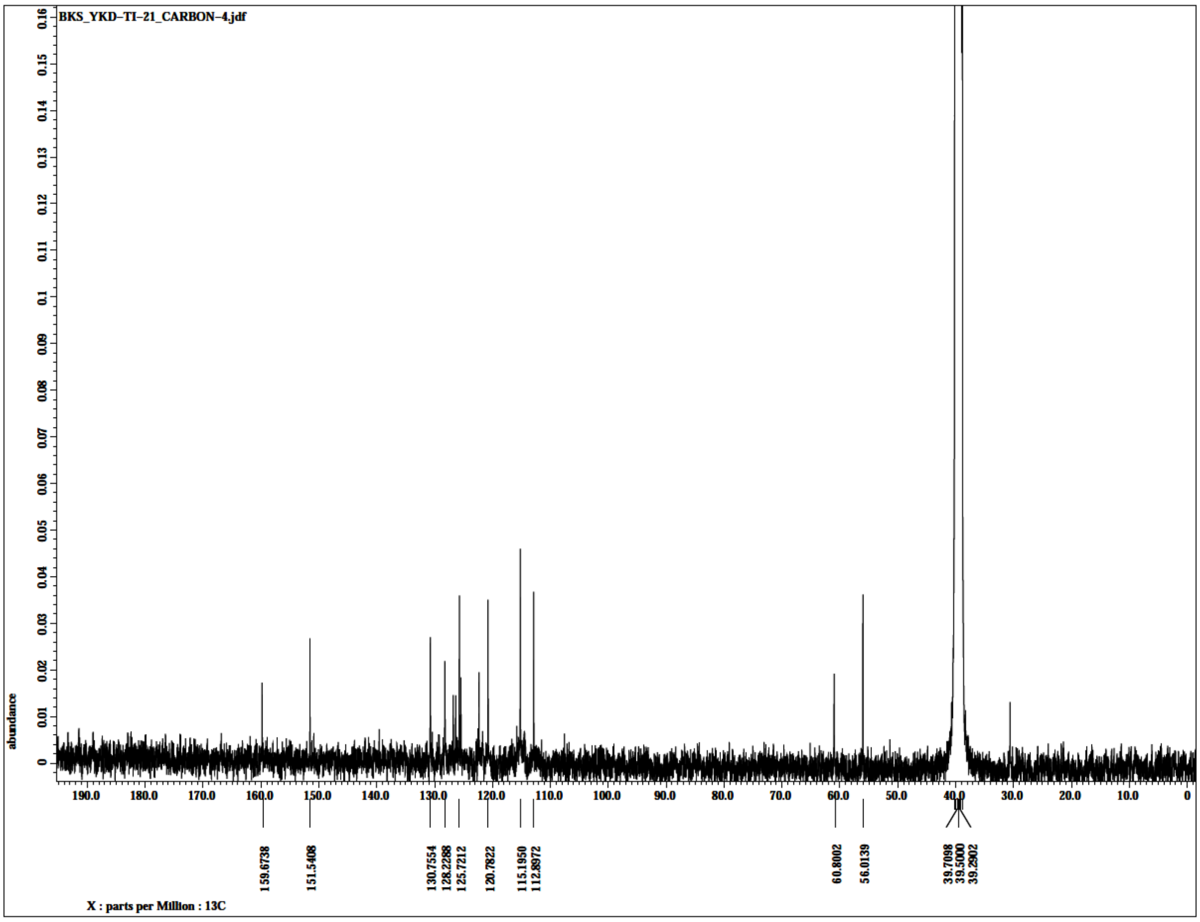
BKS_YKD–TI–21_CARBON–4.jdf
abundance
X : parts per Million : 13C
159.6738
151.5408
130.7554
128.2288
125.7212
120.7822
115.1950
112.8972
60.8002
56.0139
39.7098
39.5000
39.2902

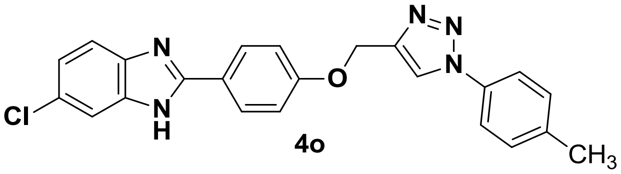
N
N
N
Cl
N
H
N
N
O
CH3

4o

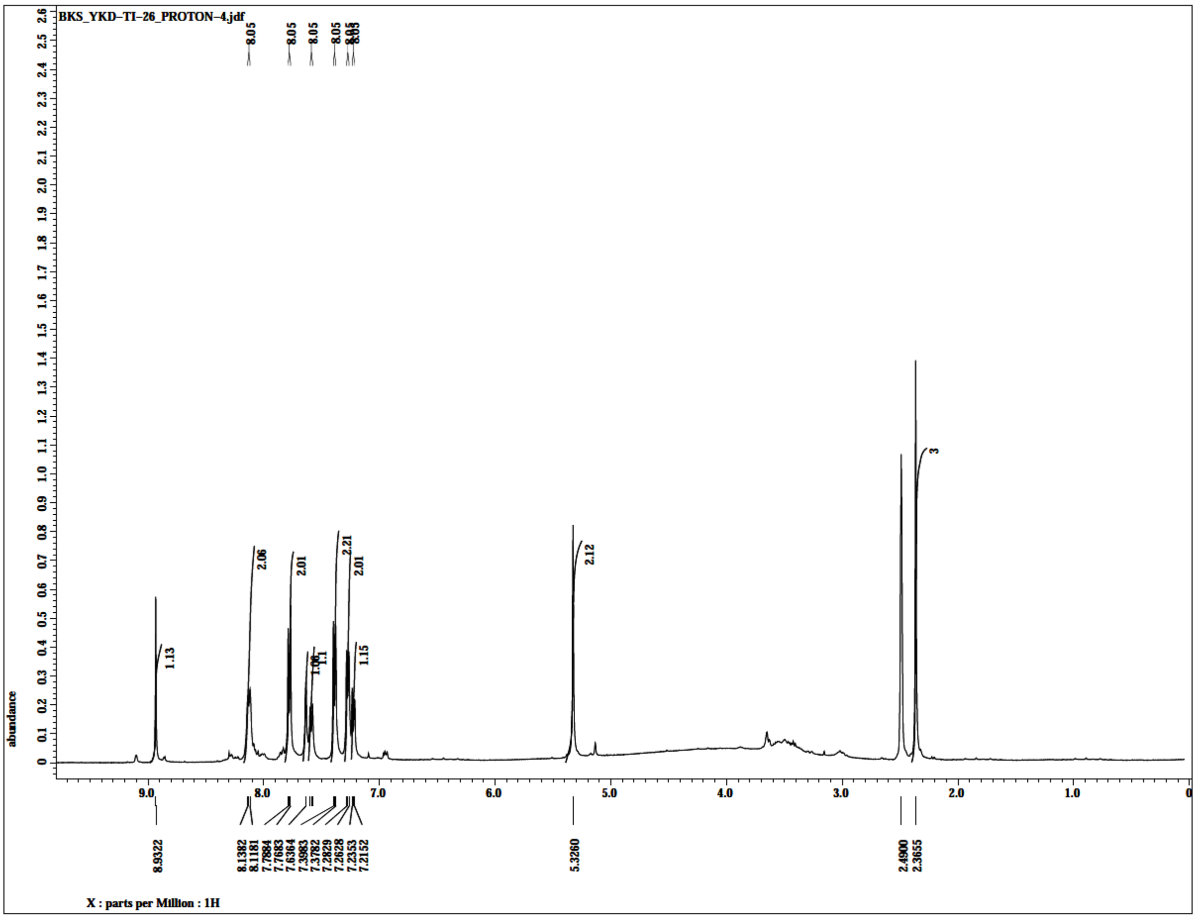
BKS_YKD–TI–26_PROTON–4.jdf
abundance
X : parts per Million : 1H
8.9322
8.1382
8.1181
7.7884
7.7683
7.6364
7.3983
7.3782
7.2829
7.2628
7.2353
7.2152
5.3260
2.4900
2.3655
1.13
2.06
2.01
2.21
2.01
1.15
2.12
3

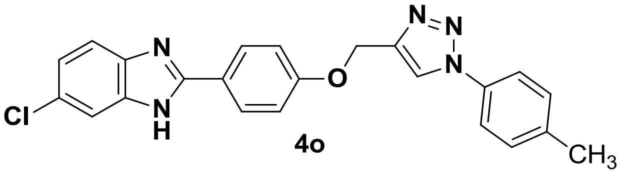
N
N
N
N
Cl
H
O
CH3

4o

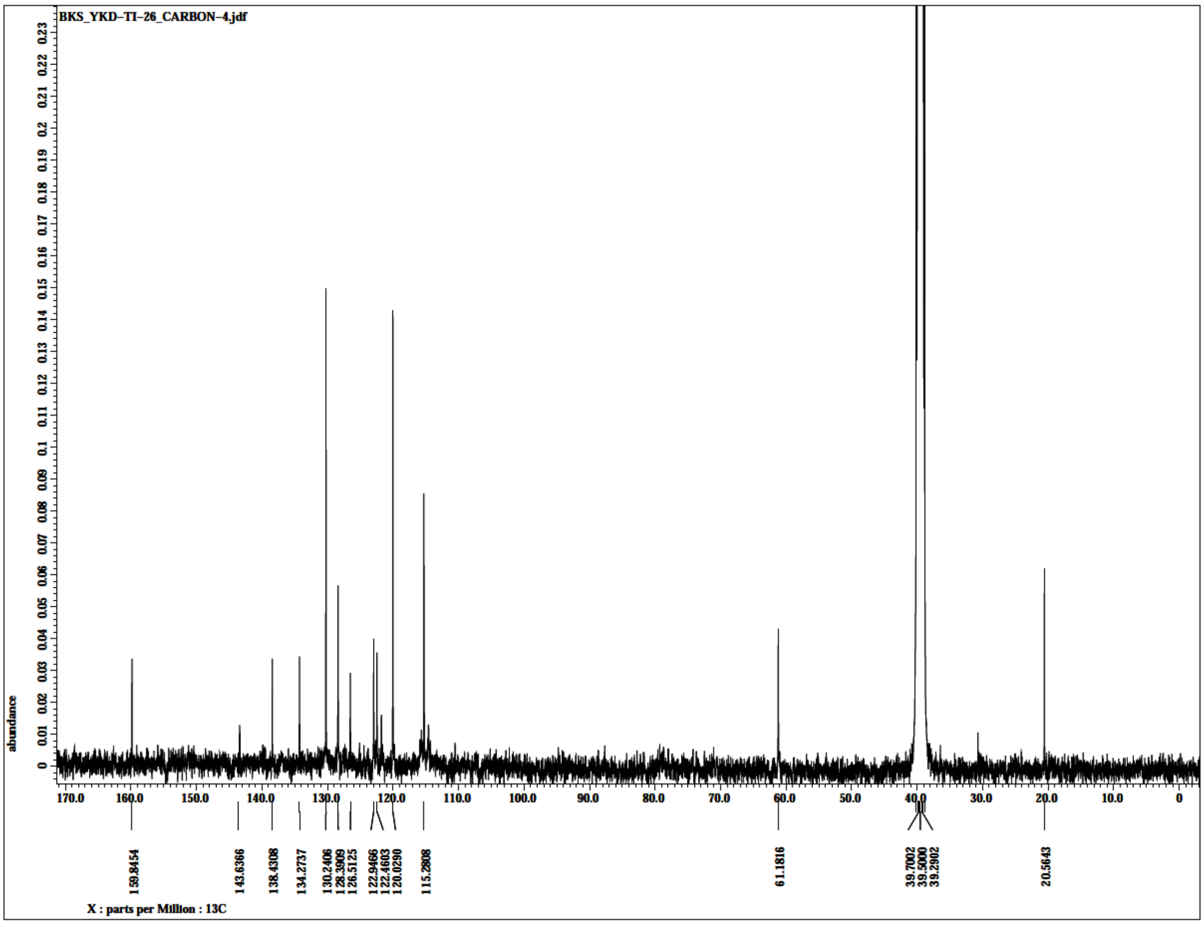
BKS_YKD–TI–26_CARBON–4.jdf
abundance
X : parts per Million : 13C
159.8454
143.6366
138.4308
134.2737
130.2406
128.3909
126.5125
122.9466
122.4603
120.0290
115.2808
61.1816
39.7002
39.5000
39.2902
20.5643

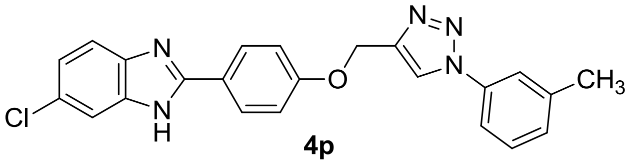
N
N
H
Cl
O
N
N
N
CH3

4p

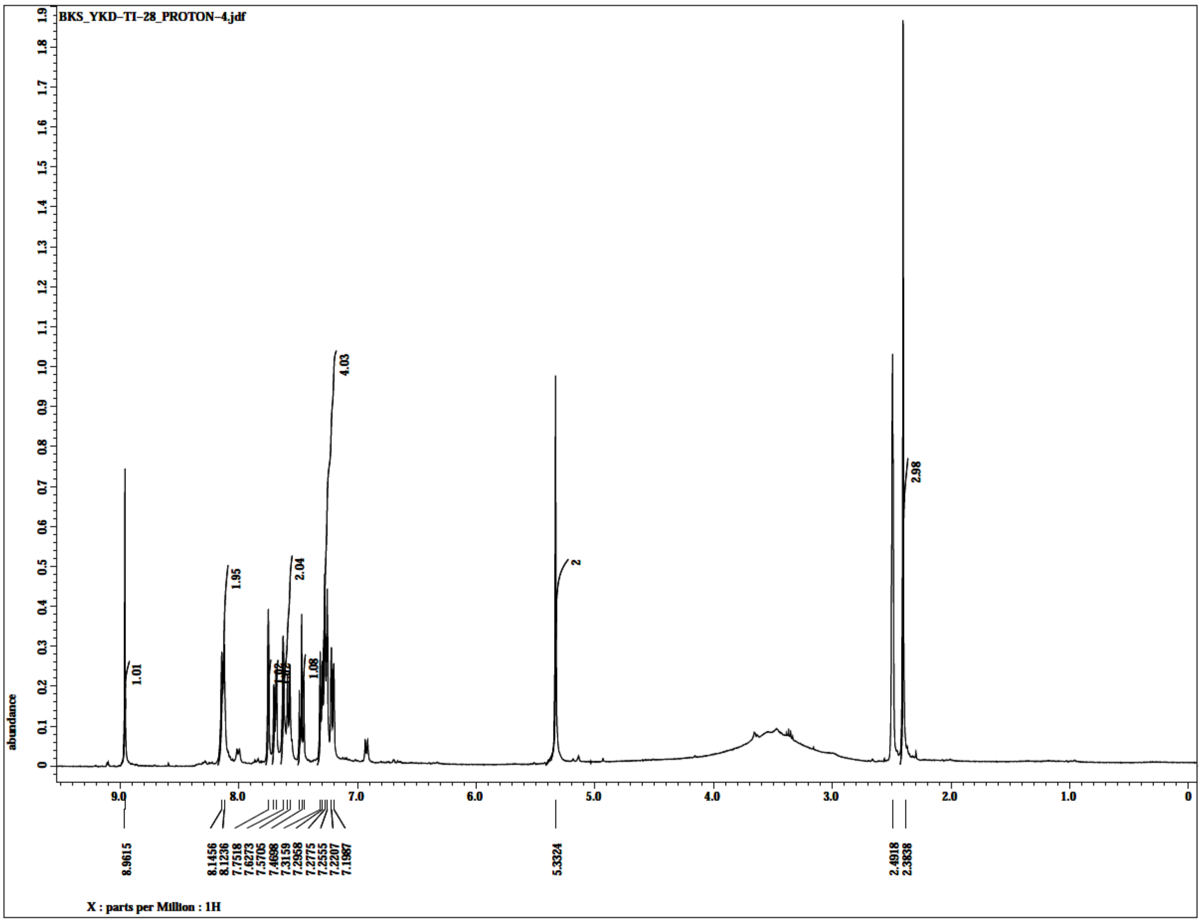
BKS_YKD–TI–28_PROTON–4.jdf
abundance
X : parts per Million : 1H
9.0
8.0
7.0
6.0
5.0
4.0
3.0
2.0
1.0
0
1.01
1.95
2.04
1.08
4.03
2
2.98
8.9615
8.1456
8.1236
7.7518
7.6273
7.5705
7.4698
7.3159
7.2958
7.2775
7.2555
7.2207
7.1987
5.3324
2.4918
2.3838

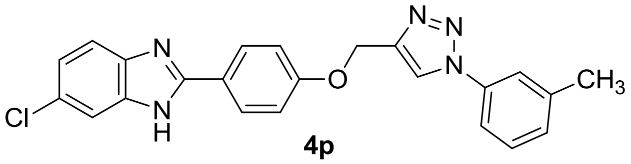

4p

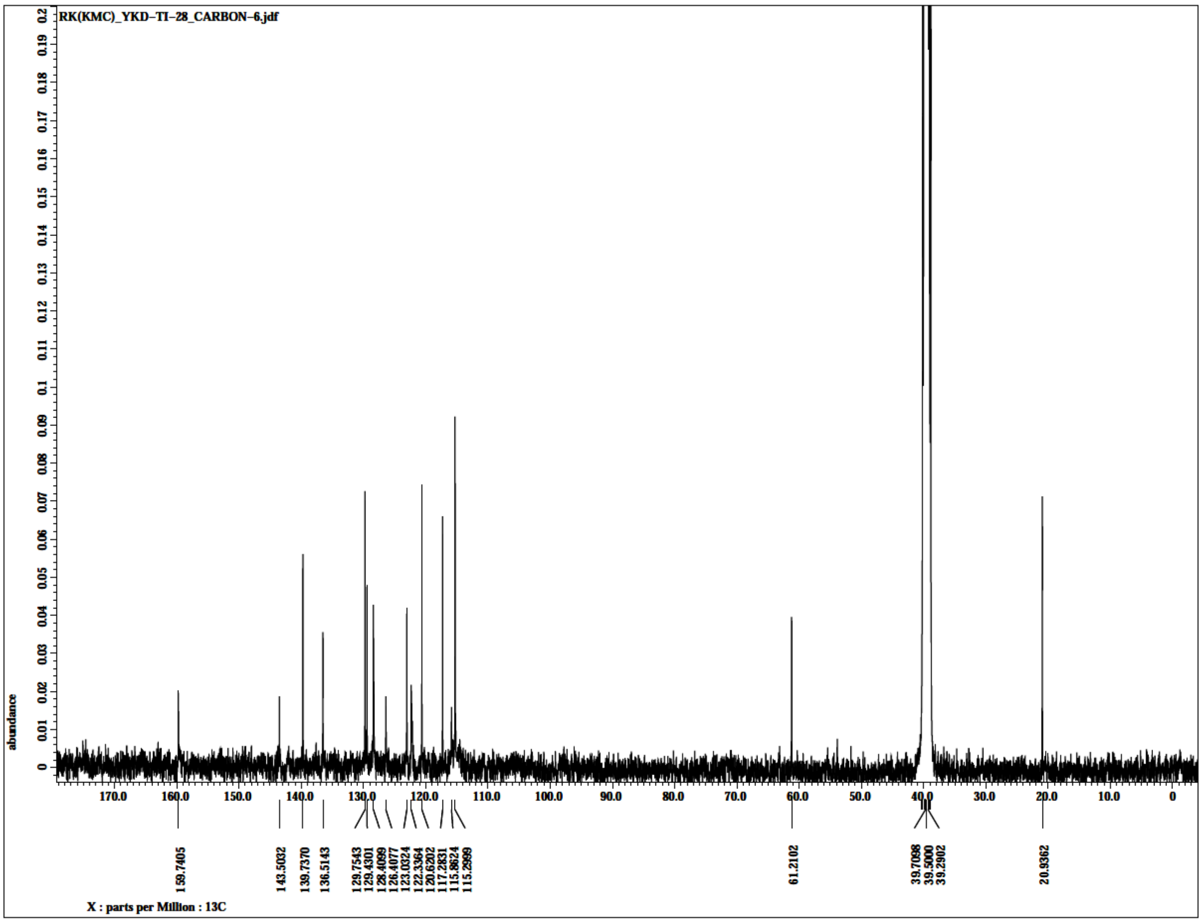

RK(KMC)_YKD–TI–28_CARBON–6.jdf
abundance
X : parts per Million : 13C
159.7405
143.5032
139.7370
136.5143
129.7543
129.4301
128.4099
126.4077
123.0324
122.3364
120.6202
117.2831
115.8624
115.2999
61.2102
39.7098
39.5000
39.2902
20.9362

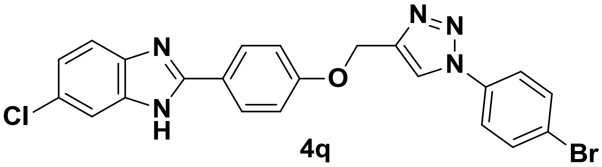

Cl
N
H
O
N
N
N
Br

4q

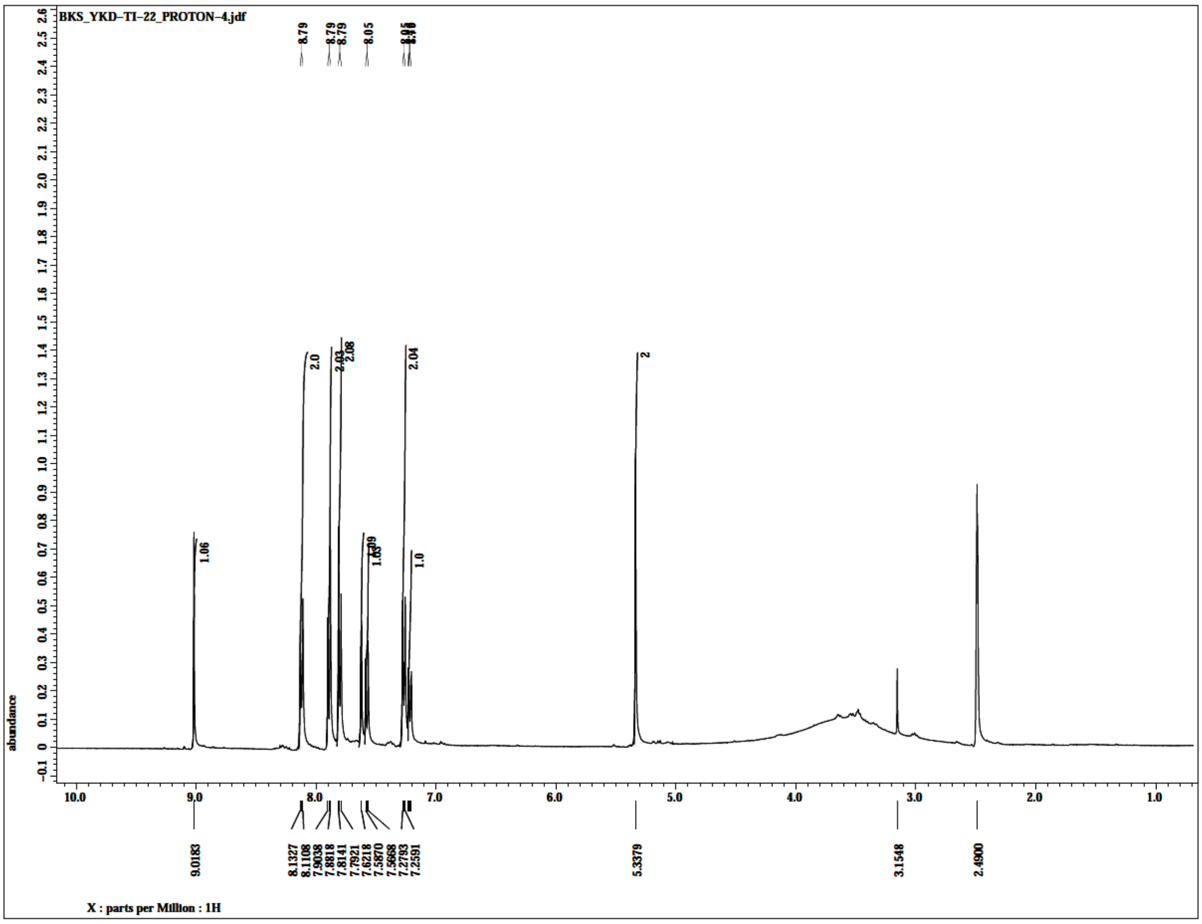

BKS_YKD-TI-22_PROTON-4.jdf
abundance
X : parts per Million : 1H
9.0183
8.1327
8.1108
7.9038
7.8818
7.8141
7.7921
7.6218
7.5870
7.5668
7.2793
7.2591
5.3379
3.1548
2.4900
1.06
2.0
2.03
2.08
1.0
2.04
2

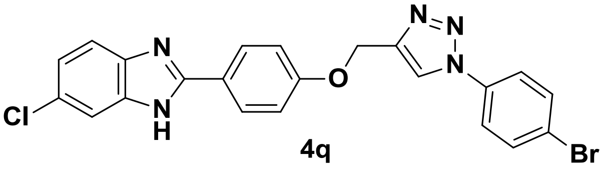
N
N
N
N
O
Cl
N
H
Br

4q

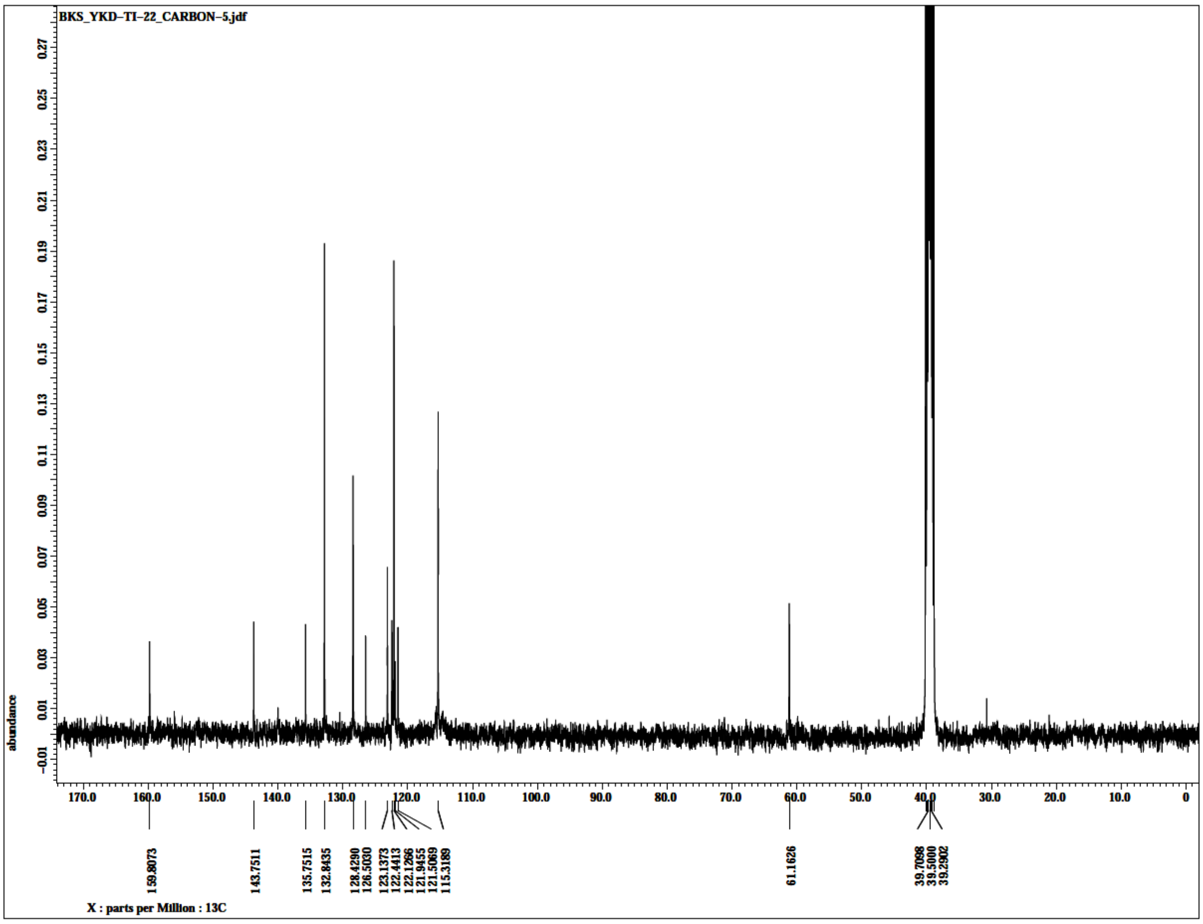
BKS_YKD–TI–22_CARBON–5.jdf
abundance
159.8073
143.7511
135.7515
132.8435
128.4290
126.5030
123.1373
122.4413
122.1266
121.9455
121.5069
115.3189
61.1626
39.7098
39.5000
39.2902
X : parts per Million : 13C

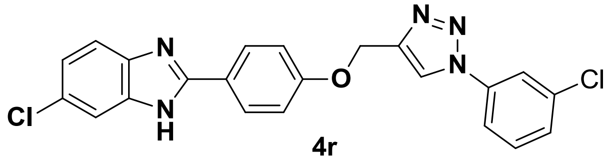

4r

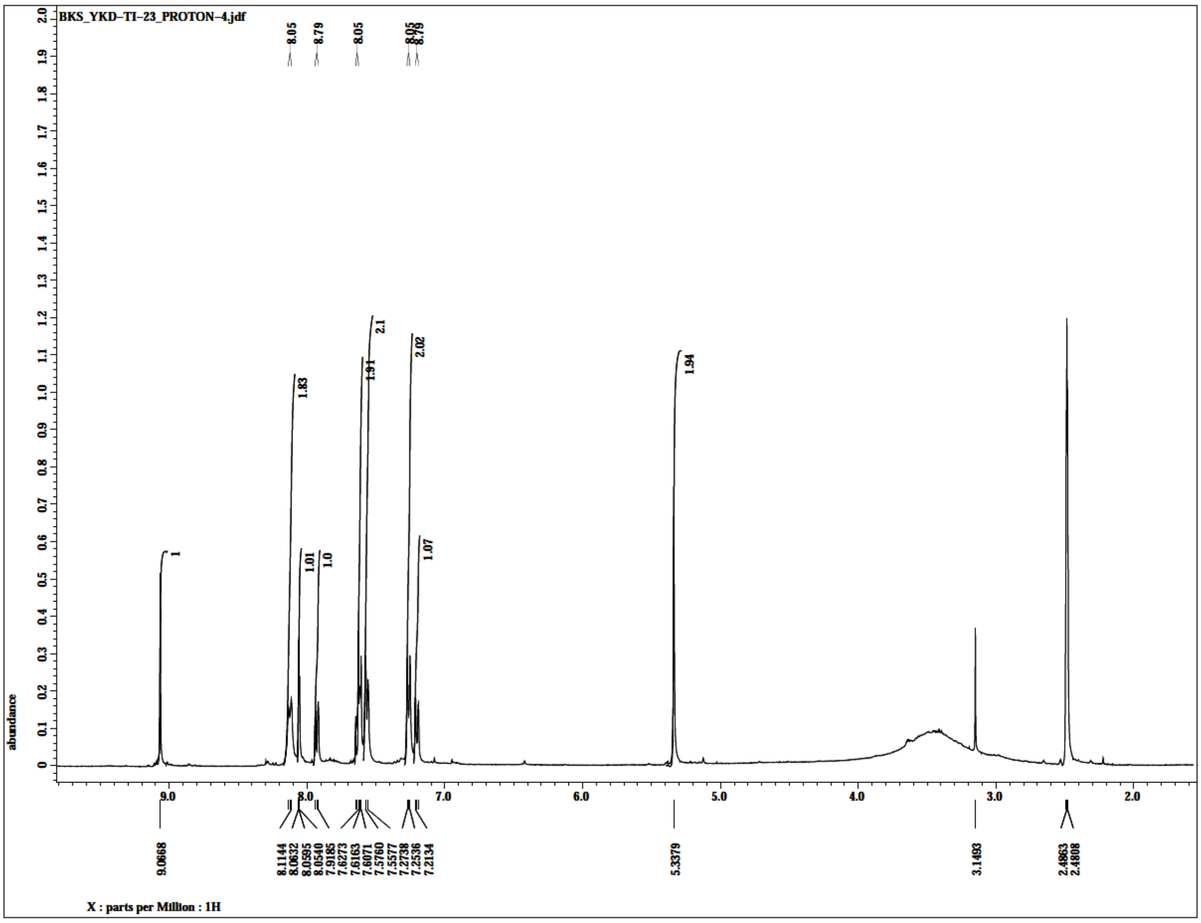

BKS_YKD-TI-23_PROTON-4.jdf
abundance
X : parts per Million : 1H
9.0668
8.1144
8.0632
8.0595
8.0540
7.9185
7.6273
7.6163
7.6071
7.5760
7.5577
7.2738
7.2536
7.2134
5.3379
3.1493
2.4863
2.4808

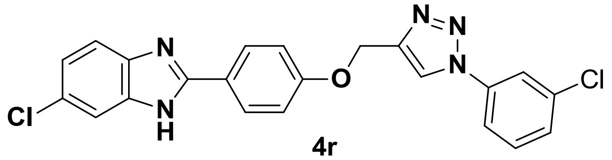

4r

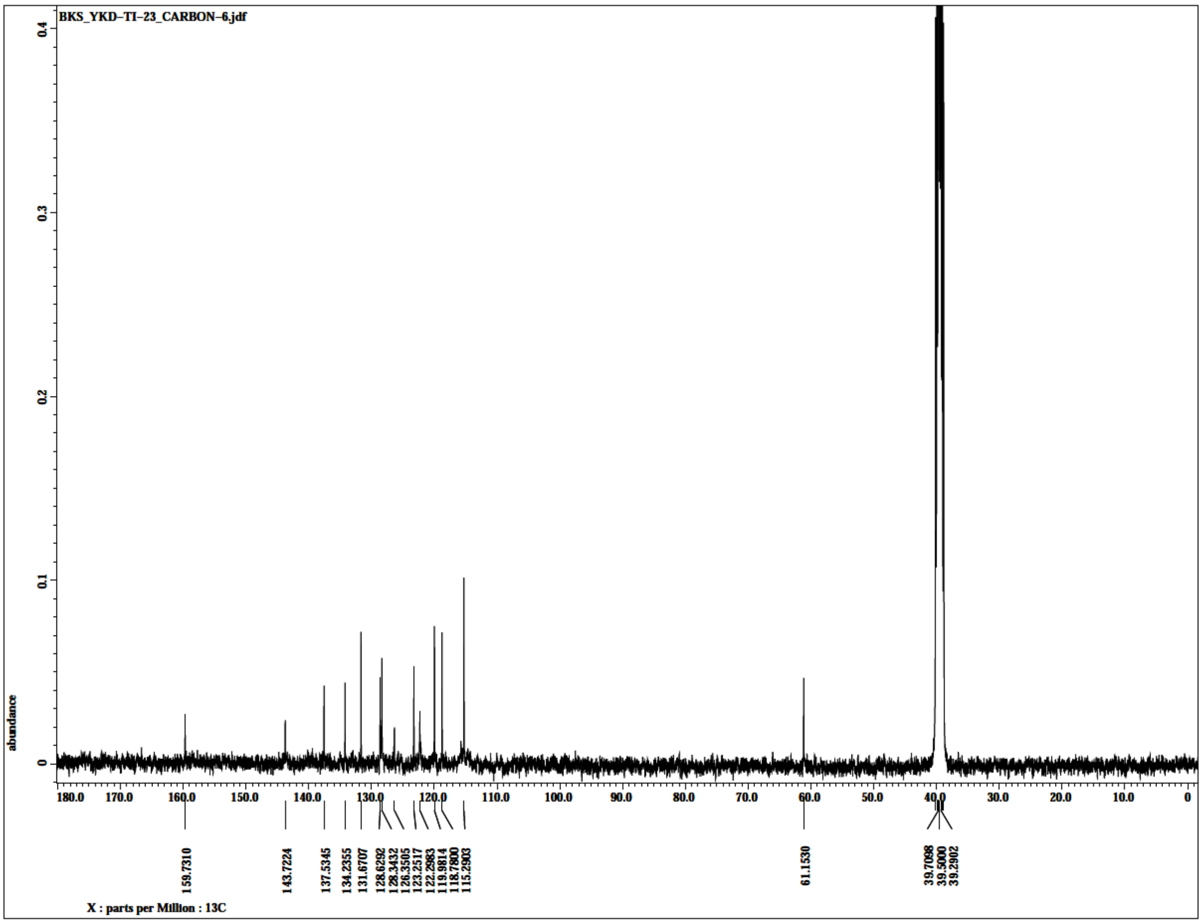
BKS_YKD–TI–23_CARBON–6.jdf
abundance
0.4
0.3
0.2
0.1
0
180.0 170.0 160.0 150.0 140.0 130.0 120.0 110.0 100.0 90.0 80.0 70.0 60.0 50.0 40.0 30.0 20.0 10.0 0
159.7310
143.7224
137.5345
134.2355
131.6707
128.6292
128.3432
126.3505
123.2517
122.2983
119.9814
118.7800
115.2903
61.1530
39.7098
39.5000
39.2902
X : parts per Million : 13C

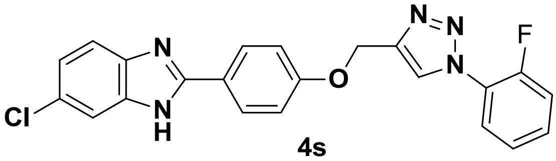
N
N
H
Cl
O
N
N
N
F

4s

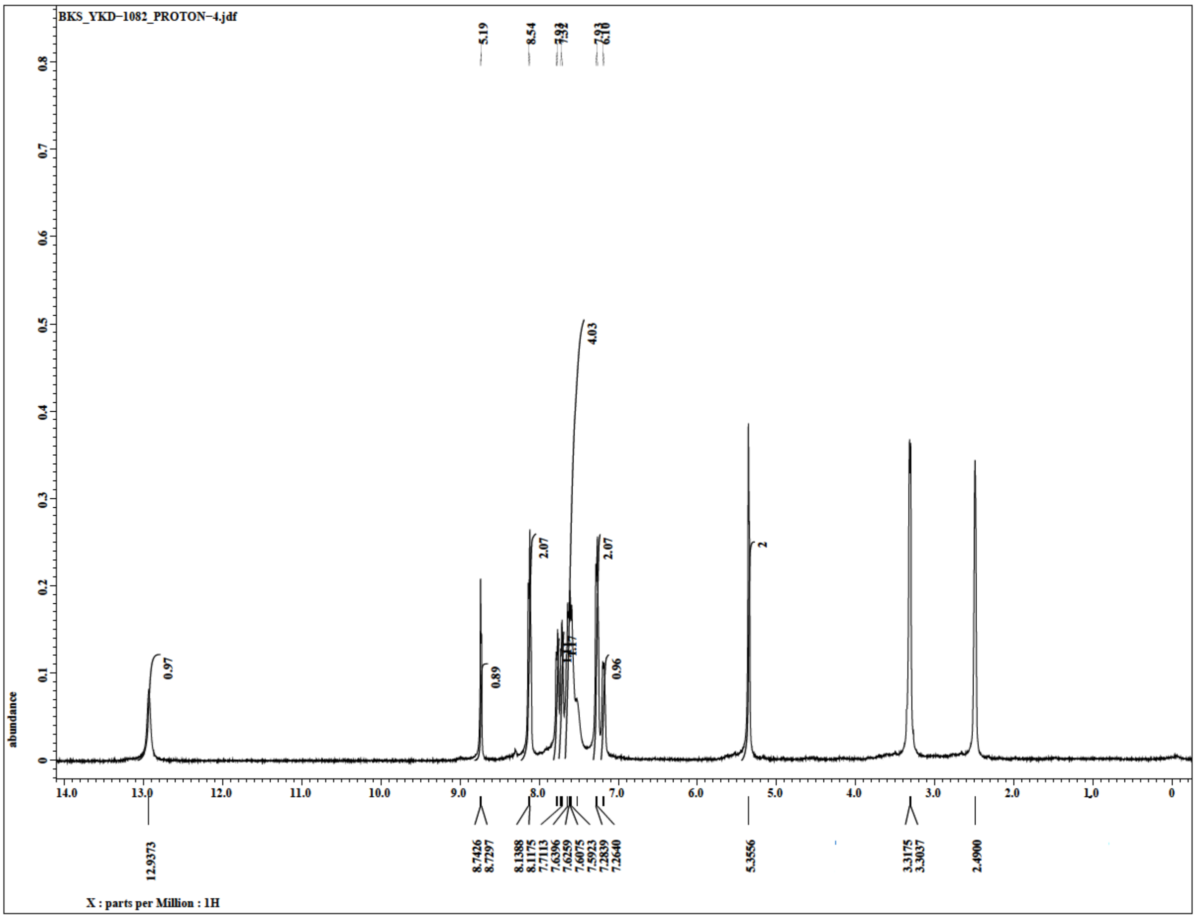
BKS_YKD-1082_PROTON-4.jdf
abundance
X : parts per Million : 1H
12.9373
8.7426
8.7297
8.1388
8.1175
7.7113
7.6396
7.6259
7.6075
7.5923
7.2839
7.2640
5.3556
3.3175
3.3037
2.4900
0.97
0.89
2.07
4.03
2.07
0.96
2

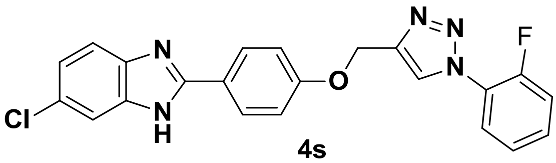

4s

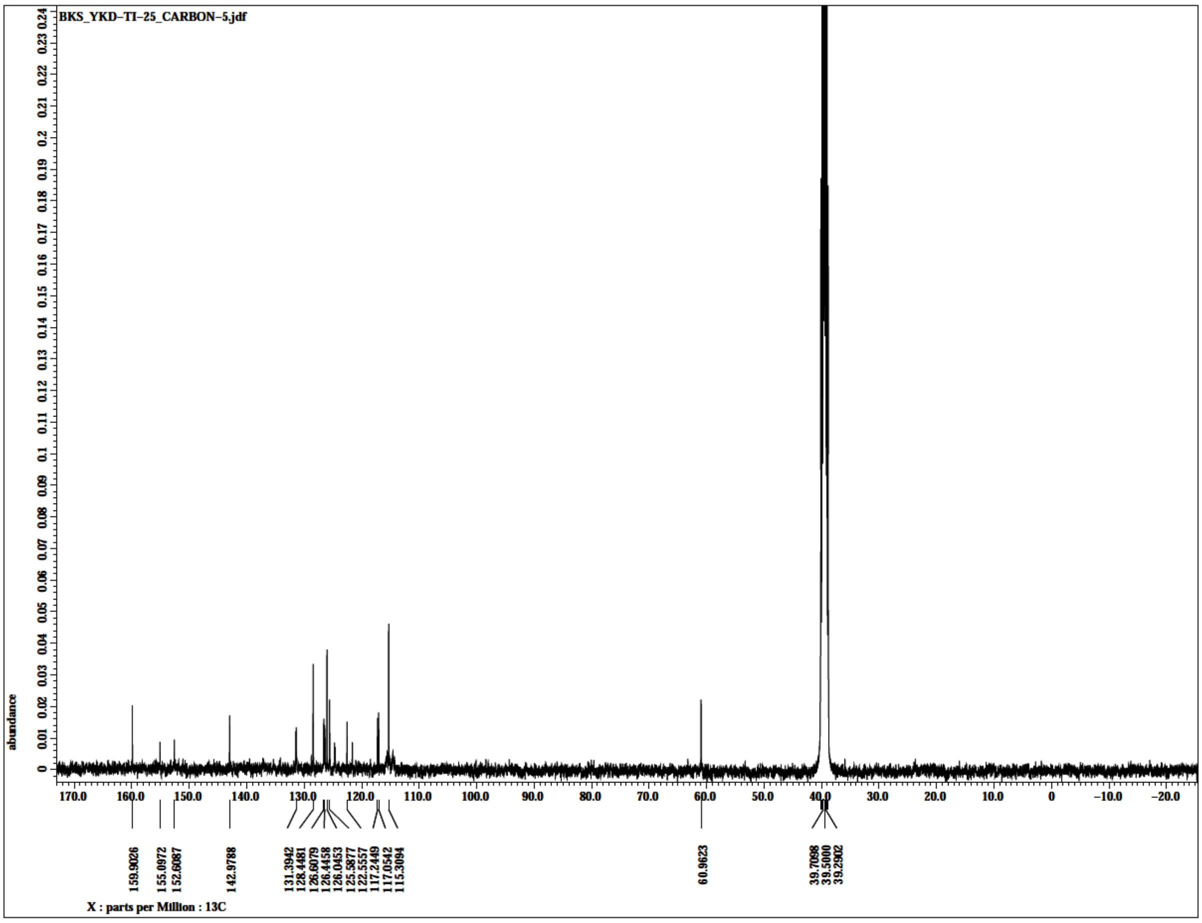

BKS_YKD-TI-25_CARBON-5.jdf
abundance
X : parts per Million : 13C
159.9026
155.0972
152.6087
142.9788
131.3942
128.4481
126.6079
126.4458
126.0453
125.5877
122.5557
117.2449
117.0542
115.3094
60.9623
39.7098
39.5000
39.2902

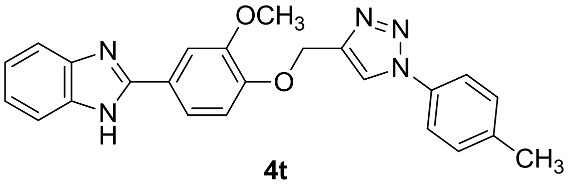

4t

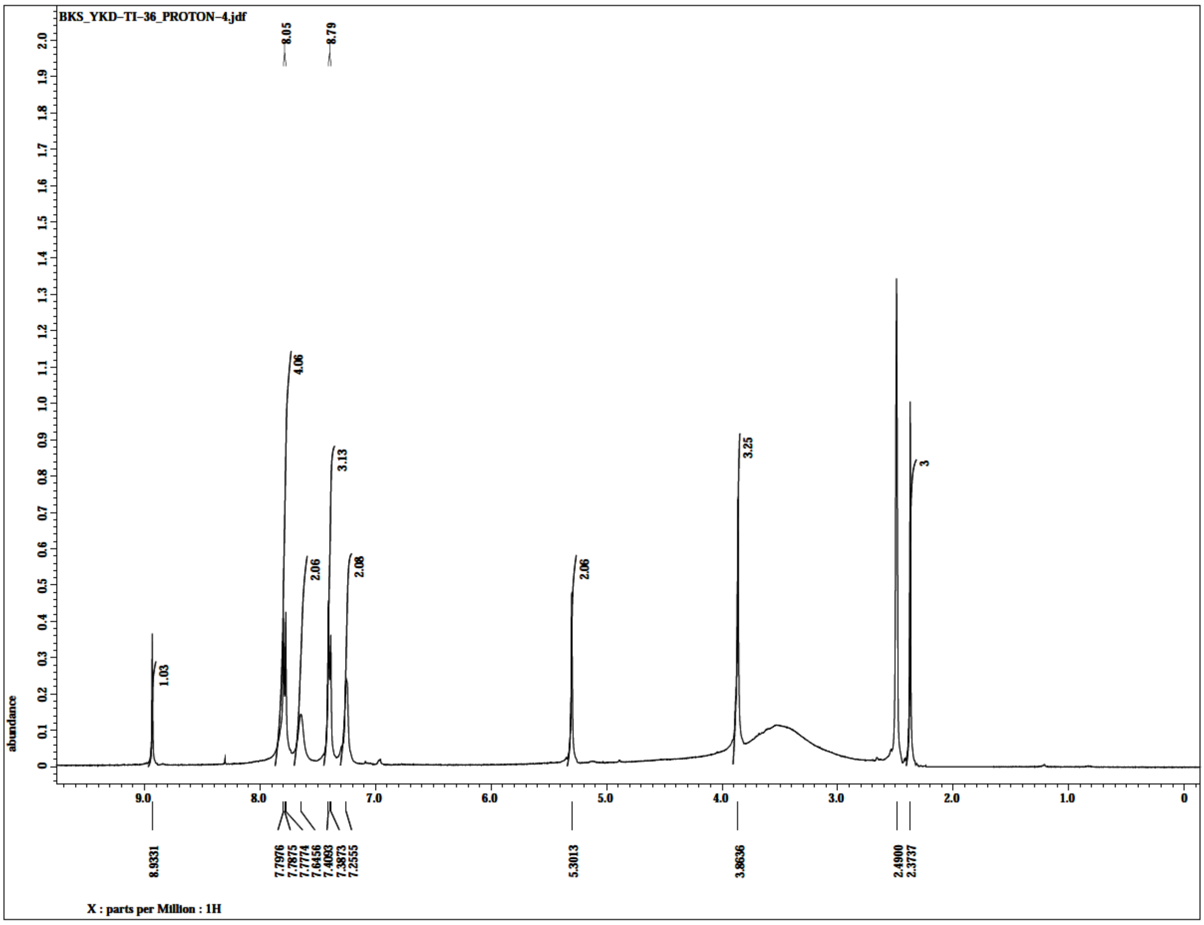

BKS_YKD–TI–36_PROTON–4.jdf
abundance
X : parts per Million : 1H
8.9331
7.7976
7.7875
7.7774
7.6456
7.4093
7.3873
7.2555
5.3013
3.8636
2.4900
2.3737
1.03
4.06
2.06
3.13
2.08
2.06
3.25
3
8.05
8.79

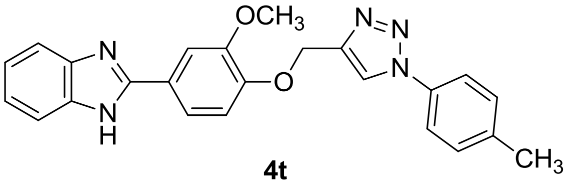
OCH3
N
N
H
O
N
N
N
CH3

4t

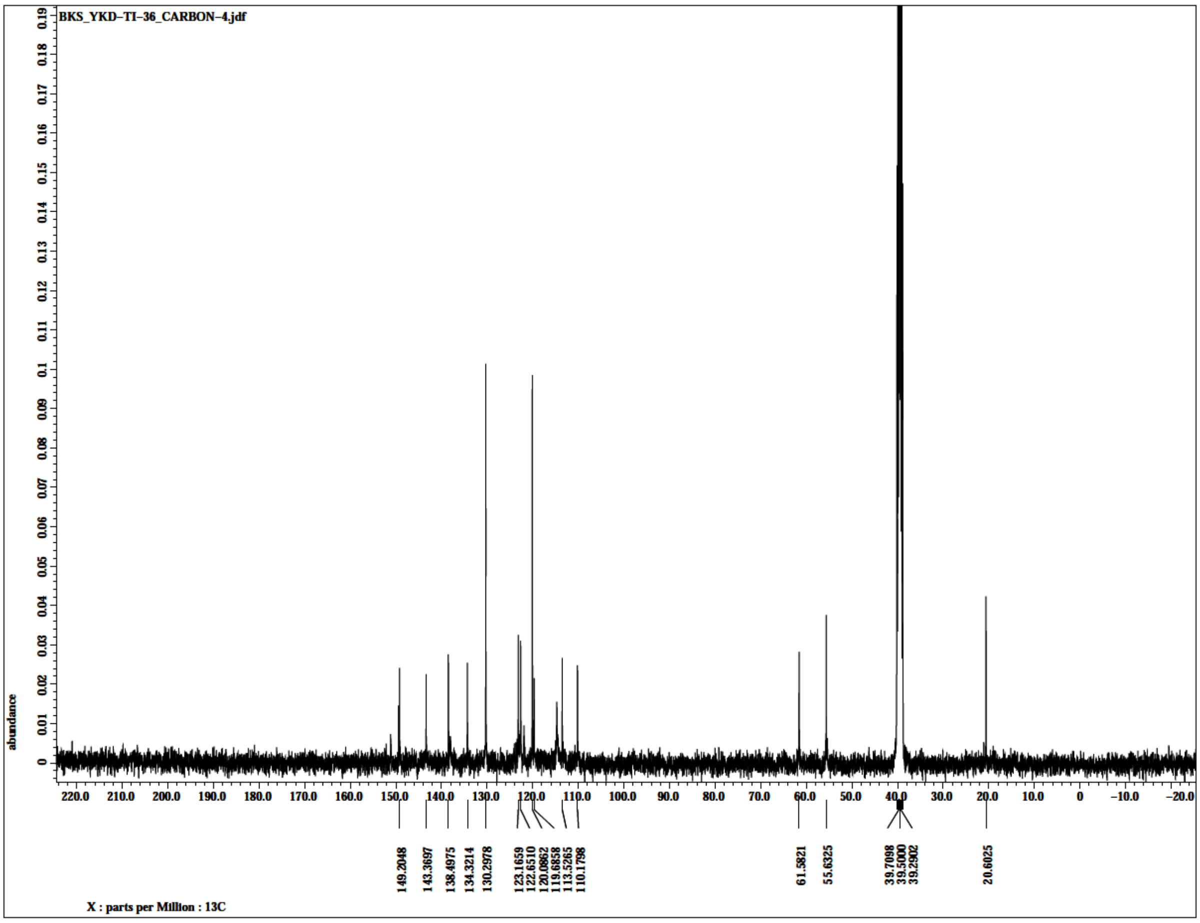
BKS_YKD−TI−36_CARBON−4.jdf
abundance
X : parts per Million : 13C
149.2048
143.3697
138.4975
134.3214
130.2978
123.1659
122.6510
120.0862
119.6858
113.5265
110.1798
61.5821
55.6325
39.7098
39.5000
39.2902
20.6025

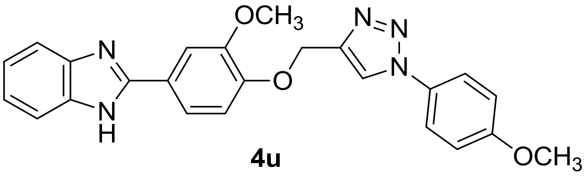

4u

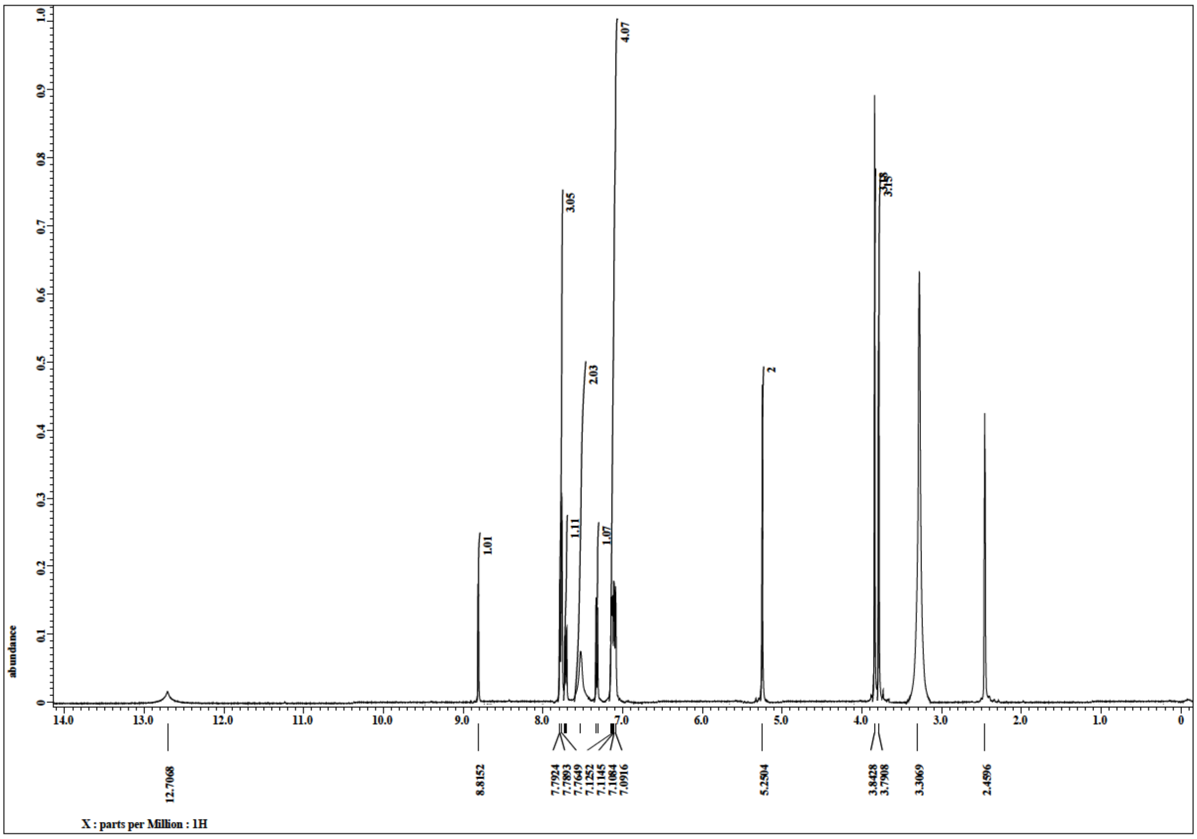

abundance
X : parts per Million : 1H
12.7068
8.8152
7.7924
7.7893
7.7649
7.1252
7.1145
7.1084
7.0916
5.2504
3.8428
3.7908
3.3069
2.4596
1.01
3.05
1.11
2.03
1.07
4.07
2

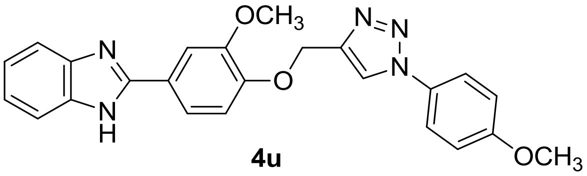
OCH3
N
N=N
N
O
N
H
OCH3

4u

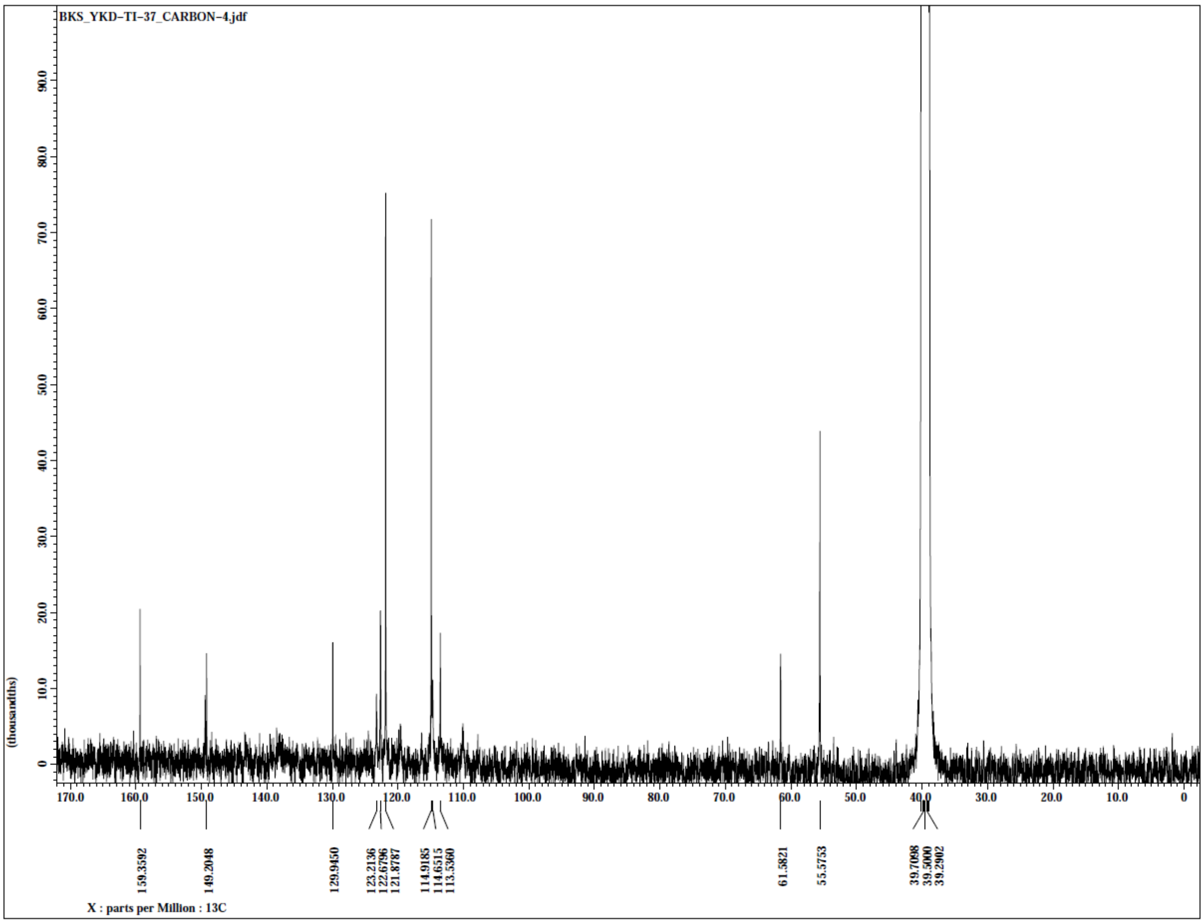
BKS_YKD-TI-37_CARBON-4.jdf
(thousandths)
X : parts per Million : 13C
159.3592
149.2048
129.9450
123.2136
122.6796
121.8787
114.9185
114.6515
113.5360
61.5821
55.5753
39.7098
39.5000
39.2902

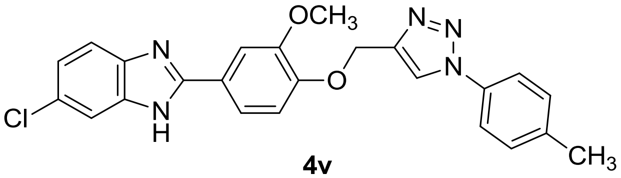
OCH3
N
N
N
N
Cl
H
O
CH3

4v

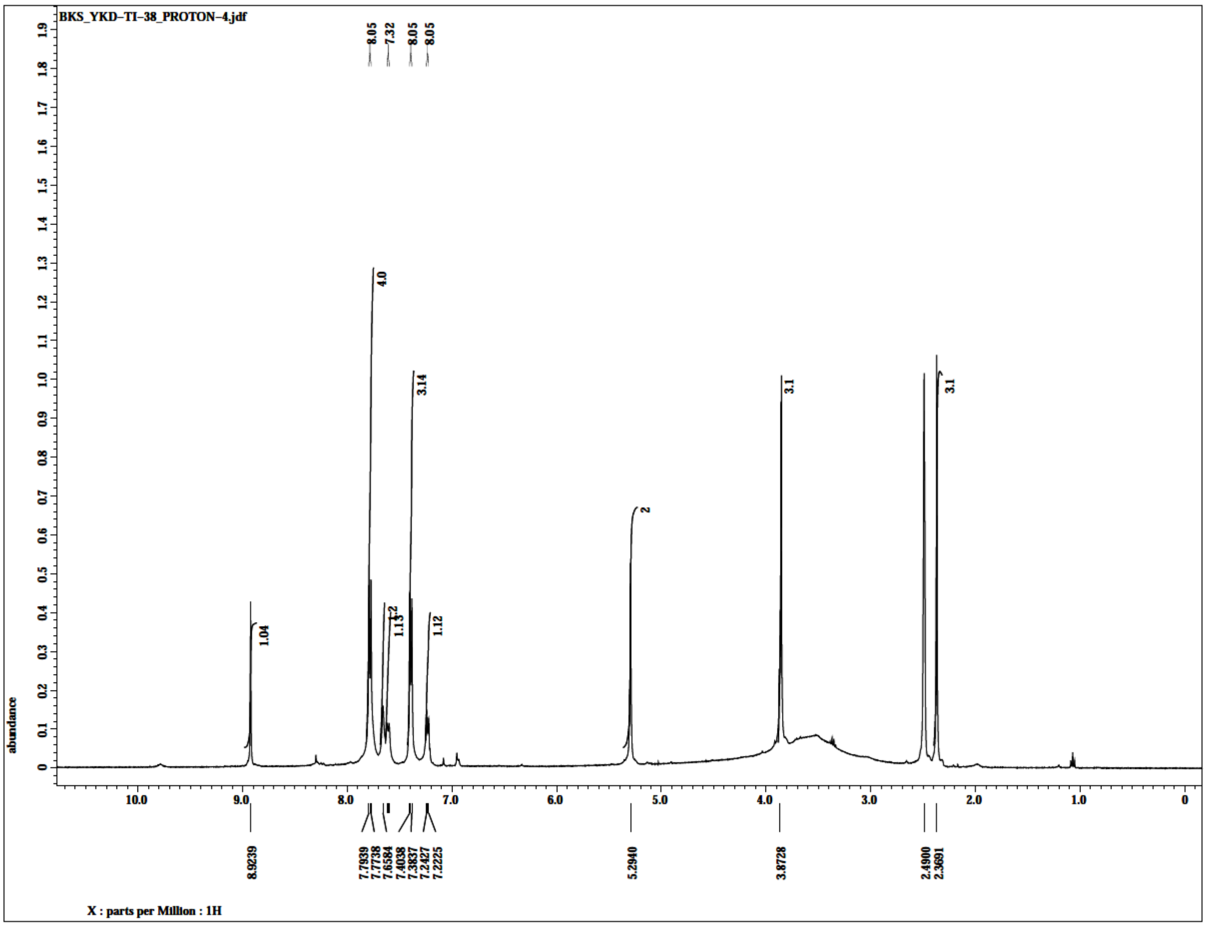
BKS_YKD-TI-38_PROTON-4.jdf
abundance
X : parts per Million : 1H
1.04
4.0
1.13
3.14
1.12
2
3.1
3.1
8.9239
7.7939
7.7738
7.6584
7.4038
7.3837
7.2427
7.2225
5.2940
3.8728
2.4900
2.3691

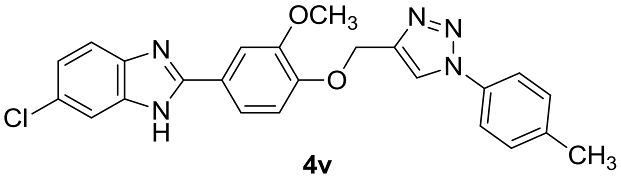

4v

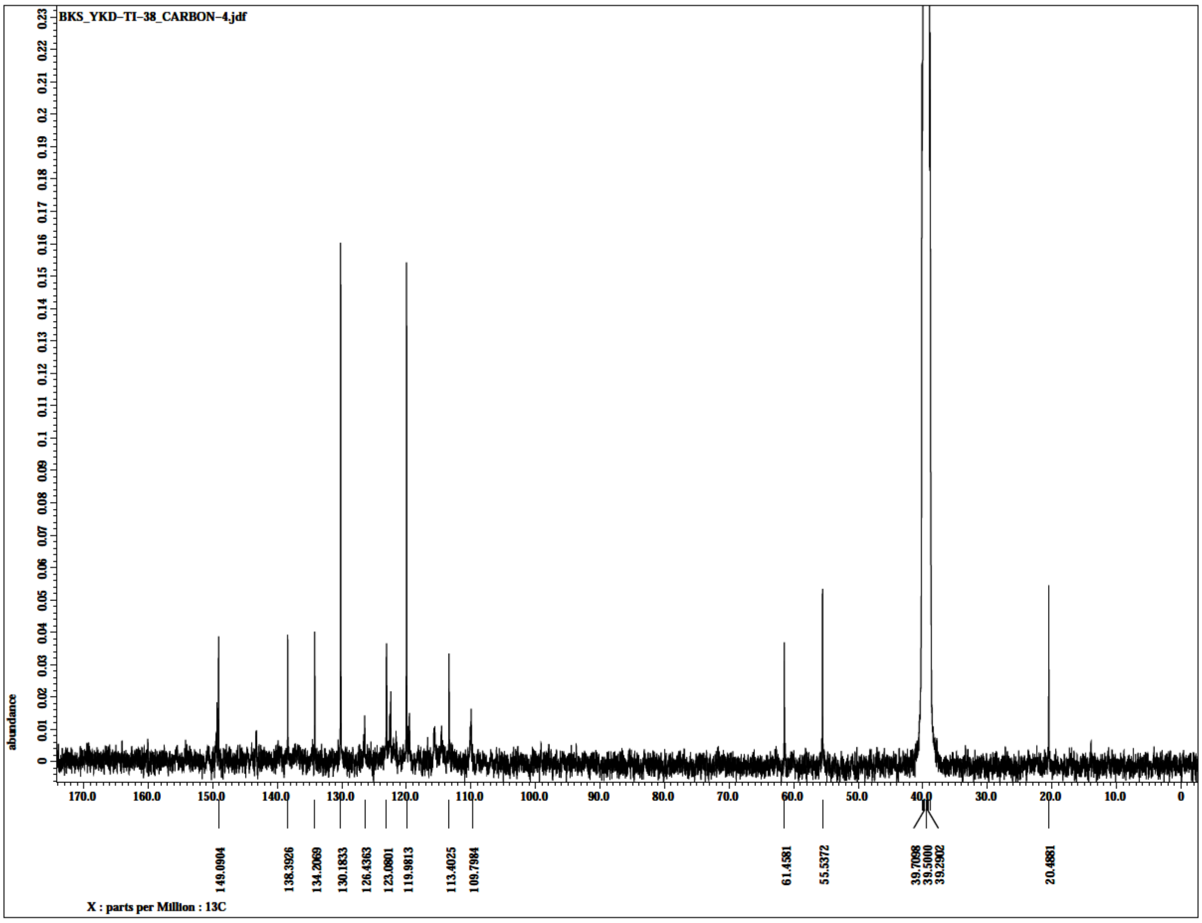
BKS_YKD-TI-38_CARBON-4.jdf
abundance
X : parts per Million : 13C
149.0904
138.3926
134.2069
130.1833
126.4363
123.0801
119.9813
113.4025
109.7984
61.4581
55.5372
39.7098
39.5000
39.2902
20.4881

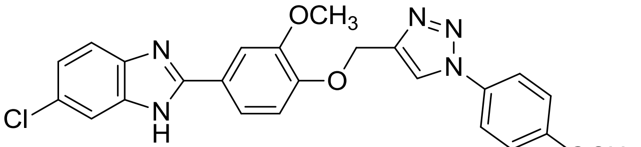
OCH3
N
N
N
N
N
H
Cl
O
OCH3

4w

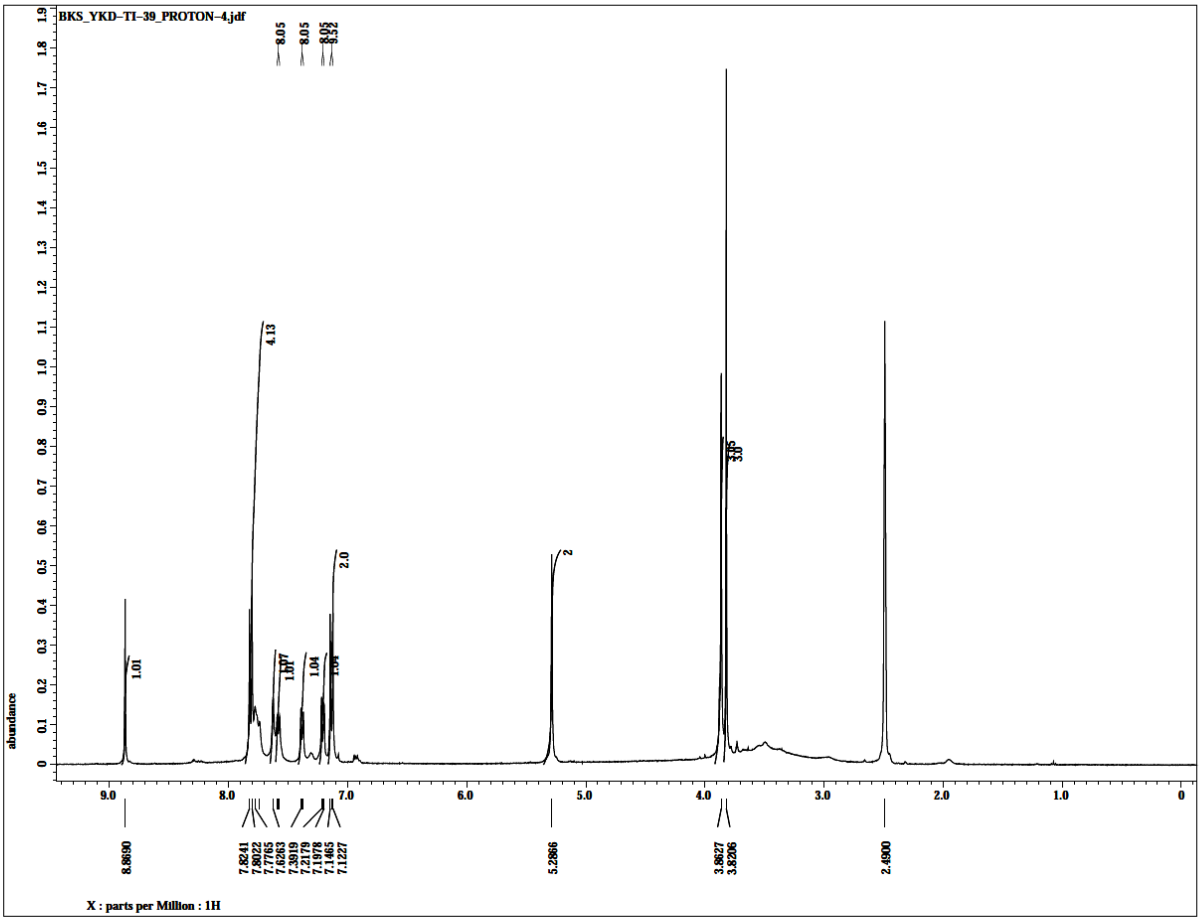
BKS_YKD-TI-39_PROTON-4.jdf
abundance
X : parts per Million : 1H
8.8690
7.8241
7.8022
7.7765
7.6263
7.3919
7.2179
7.1978
7.1465
7.1227
5.2866
3.8627
3.8206
2.4900

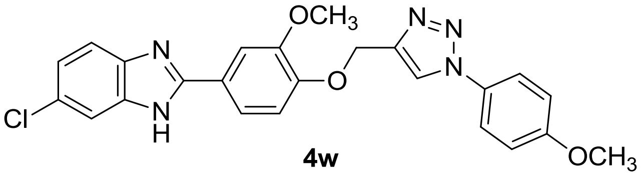
OCH3
N=N
N
O
N
Cl
H
OCH3

4w

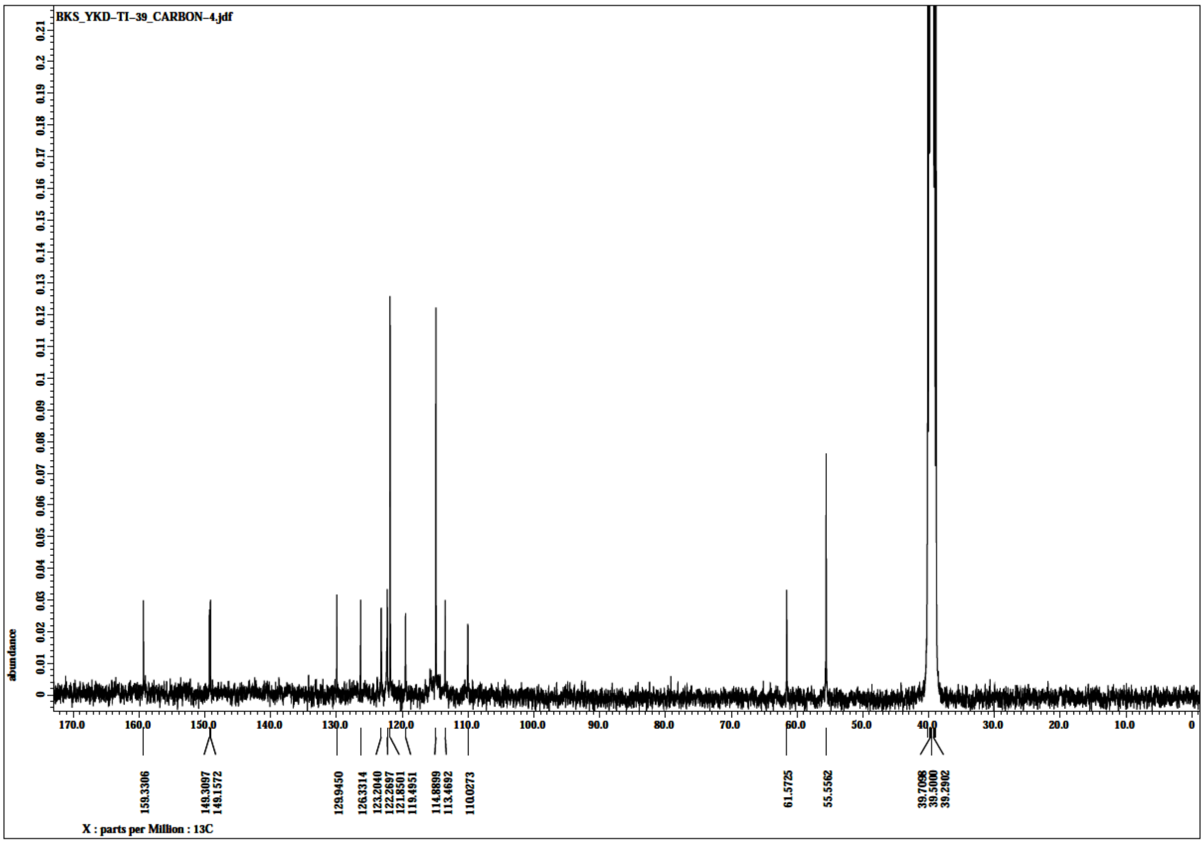
BKS_YKD–TI–39_CARBON–4.jdf
abundance
X : parts per Million : 13C
159.3306
149.3097
149.1572
129.9450
126.3314
123.2040
122.2697
121.8501
119.4951
114.8899
113.4692
110.0273
61.5725
55.5562
39.7098
39.5000
39.2902
